# Discovery of new quinolines as potent colchicine binding site inhibitors: design, synthesis, docking studies, and anti-proliferative evaluation

**DOI:** 10.1080/14756366.2021.1883598

**Published:** 2021-02-15

**Authors:** Mohamed Hagras, Moshira A. El Deeb, Heba S. A. Elzahabi, Eslam B. Elkaeed, Ahmed B. M. Mehany, Ibrahim H. Eissa

**Affiliations:** a Department of Pharmaceutical Organic Chemistry, Faculty of Pharmacy (Boys), Al-Azhar University, Cairo, Egypt; b Department of Pharmaceutical Organic Chemistry, Faculty of Pharmacy (Girls), Al-Azhar University, Cairo, Egypt; c Department of Pharmaceutical Medicinal Chemistry & Drug Design, Faculty of Pharmacy (Girls), Al-Azhar University, Cairo, Egypt; d Department of Pharmaceutical Sciences, College of Pharmacy, AlMaarefa University, Ad Diriyah, Riyadh, Saudi Arabia; e Department of Zoology, Faculty of Science, Al-Azhar University, Cairo, Egypt; f Department of Pharmaceutical Medicinal Chemistry & Drug Design, Faculty of Pharmacy (Boys), Al-Azhar University, Cairo, Egypt

**Keywords:** Cancer, colchicine binding site inhibitors, docking, quinoline, tubulin polymerisation

## Abstract

Discovering of new anticancer agents with potential activity against tubulin polymerisation is still a promising approach. Colchicine binding site inhibitors are the most relevant anti-tubulin polymerisation agents. Thus, new quinoline derivatives have been designed and synthesised to possess the same essential pharmacophoric features of colchicine binding site inhibitors. The synthesised compounds were tested *in vitro* against a panel of three human cancer cell lines (HepG-2, HCT-116, and MCF-7) using colchicine as a positive control. Comparing to colchicine (IC_50_ = 7.40, 9.32, and 10.41 µM against HepG-2, HCT-116, and MCF-7, respectively), compounds **20**, **21**, **22, 23, 24, 25, 26,** and **28** exhibited superior cytotoxic activities with IC_50_ values ranging from 1.78 to 9.19 µM. In order to sightsee the proposed mechanism of anti-proliferative activity, the most active members were further evaluated *in vitro* for their inhibitory activities against tubulin polymerisation. Compounds **21** and **32** exhibited the highest tubulin polymerisation inhibitory effect with IC_50_ values of 9.11 and 10.5 nM, respectively. Such members showed activities higher than that of colchicine (IC_50_ = 10.6 nM) and CA-4 (IC_50_ = 13.2 nM). The impact of the most promising compound **25** on cell cycle distribution was assessed. The results revealed that compound **25** can arrest the cell cycle at G2/M phase. Annexin V and PI double staining assay was carried out to explore the apoptotic effect of the synthesised compounds. Compound **25** induced apoptotic effect on HepG-2 thirteen times more than the control cells. To examine the binding pattern of the target compounds against the tubulin heterodimers active site, molecular docking studies were carried out.

## 1. Introduction

Cancer is the uncontrolled growth of abnormal cells^1^. There are about 100 types of cancer which need diagnosis and treatment^2^. In 2018, according to National Cancer Institute (NIH), approximately 1,735,350 new cases of cancer have been diagnosed in the United States and 609,640 people died from the disease. Cancer of breast, liver, colon, and rectum are common disease with high rate of incidence^3^. The current approaches of cancer treatment include surgery, radiation, chemotherapy; and hormonal treatment^4^.

Microtubules are important cytoskeletal structures that have a crucial role in cell division^5^, which make them attractive targets for design of new anticancer agents^6^^,^^7^. Microtubules are composed of two subunits of *α*- and *β*-tubulin heterodimers. These subunits are arranged in slender-shaped filamentous tubes with many micrometres long^8^. Some natural products that target the tubulin and the microtubule system- also referred to as anti-mitotics–still important counterparts in combination chemotherapy for the treatment of several malignancies^9^^,^^10^.

**Figure 1.**
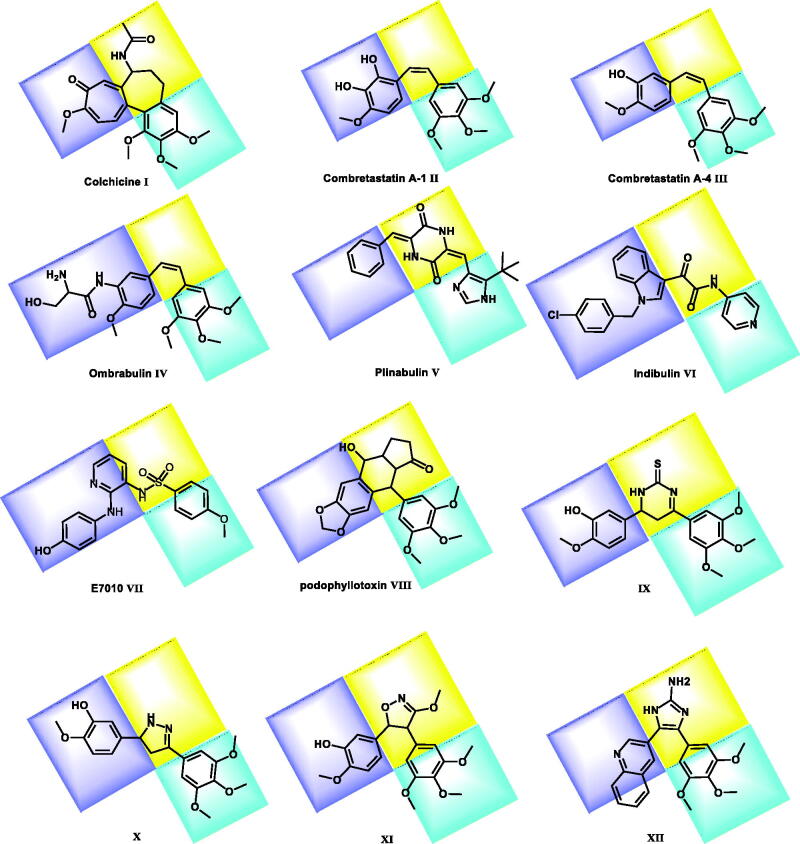
Reported colchicine binding site inhibitors.

The tubulin heterodimer comprises at least three binding sites: the paclitaxel, vinblastine, and colchicine binding sites (Figure 1)^11^. There are many drugs used in clinical oncology acting on the paclitaxel and vinblastine binding sites^12^^,^^13^. These drugs are highly potent but there are some limitation in clinical use for many reasons: development of multi-drug resistance, high lipophilicity, low water solubility, intravenous administration due to poor water solubility^14^. Aforementioned drawbacks can largely overcome by use of tubulin inhibitors that bind to the colchicine binding site. Such inhibitors have therapeutic advantages: high water solubility so that it can be administered orally and there is no multi-drug resistance^15^.

Colchicine binding site inhibitors (CBSI) produce their biological activities by suppressing the vital process of tubulin assembly, and consequently suppressing microtubule formation^14^. Colchicine **I**, the most famous inhibitors in this category, binds to tubulin very tightly, but there is no any compound in this group has a significant use in treatment of cancer^16^. combretastatin A-1 (CA-1) **II** and combretastatin A- 4 (CA-4) **III** are combretastatin analogs having microtubule inhibitory activity with limited value due to their low water solubility^17^. To improve their water solubility, such analogs were designed as prodrugs of monosodium phosphate salt. These prodrugs can be metabolised *in vivo* into CA-1 and CA-4 as active components^18^^,^^19^. CA-4P showed no bone marrow toxicity, stomatitis, and hair loss in phase II clinical trial^20^. One of CA-4 analogs is ombrabulin **IV** which has better water solubility, oral activity, enhanced anti-cancer activity and decreased adverse effects^14^.

Plinabulin **V** is an active molecule against non-small cell lung cancer^21^. It restricts tubulin polymerisation with immune-enhancing effects^22^^,^^23^. Indibulin **VI** has an effective antitumor activity with a lower side effects^24^. The antitumor activity of indibulin is believed to be related to its effects on microtubules^25^.

E7010 **VI**I is an orally bioavailable tubulin-binding agent that was developed for cancer treatment. It is a sulphonamide derivatives having an antimitotic effect. It binds to the colchicine site on β-tubulin subunit, leading to cell cycle arrest at the G2/M phase, resulting in cellular apoptosis^26^. It exhibited a broad spectrum of antitumor activity *in vitro* and *in vivo*^26^. Its clinical trial indicated that it has dose-limiting toxicities included abdominal pain, constipation, and fatigue^14^.

Podophyllotoxin **VIII**, a naturally occurring anticancer agent, binds to the colchicine site of tubulin leading to inhibition of the tubulin assembly into microtubules^27^. It has severe toxicity which limits its clinical application as a cancer therapy. However, podophyllotoxin is still considered an attractive lead compound for the generation of new antitumor agents^28^.

Moreover, several derivatives (e.g. compounds **IX**^29^, **X**^30^, **XI**^31^, and **XII**^32^) has been synthesised and evaluated as tubulin inhibitors targeting the colchicine binding site. These compounds were modified and tested to find highly potent agents for treatment of cancer.

In continuation to our previous efforts of design and synthesis of new anticancer agents^33–42^, a new series of quinoline derivatives were designed and synthesised. The synthesised derivatives have the same pharmacophoric features of CBSI to examine their effect as anticancer agents with potential tubulin inhibitory activities targeting the colchicine binding site.

### 1.1. Rational of molecular design

**Figure 2.**
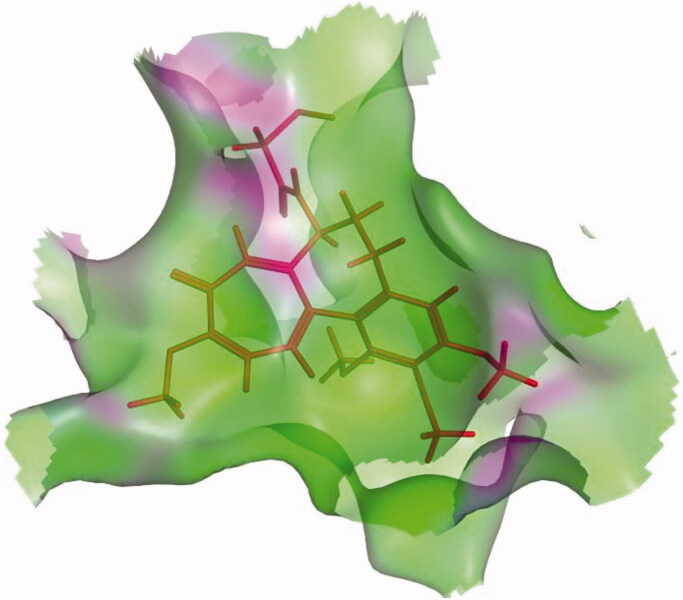
The colchicine binding site having a funnel shape.^39^

The colchicine binding site is a funnel shaped with a volume of about 600 Å. the cavity of the active site is surrounded by Ala180α, Asn101α, Thr179α, Val181α, Thr314β, Asn349β, Lys352β, Asn350β, Tyr202β, Thr239β, Asp251β, Cys241β, Asn258β, Leu242β, Leu248β, Leu252β, Ile378β, Leu255β, Val318β, Ala250β, Lys254β, Ala316β, Met259β, Ala317β, Val238β, Thr353β, and Ala354β residues (Figure 2)^39^^,^^43^.

**Figure 3.**
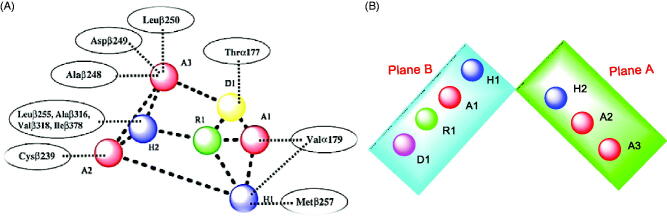
(A) Seven pharmacophoric features of colchicine binding site inhibitors: three hydrogen bond acceptors (A1, A2 & A3), one hydrogen bond donor (D1), two hydrophobic centres (H1 & H2), and one planar group (R1) (based on Ref.^14^^,^^44^). (B) The pharmacophoric model with two planes: plane A (green) points A1, D1, H1 and R1, Plane B (turquoise) consists of points A2, A3, and H2, and (based on Ref.^39^^,^^44^).

It was reported that the colchicine binding site inhibitors possess seven essential pharmacophoric groups; one hydrogen bond donor (D1), three hydrogen bond acceptors (A1, A2, and A3), two hydrophobic centres (H1 and H2), and one planar group (R1)^14^^,^^44^ (Figure 3(A)). The different pharmacophoric features are arranged in two planes. The Features A1, D1, H1, and R1 lie in plane A, and features A2, A3, and H2 lie in plane B. The angle between the two planes is about **45**^39^^,^^44^ (Figure 3(B)).

**Figure 4.**
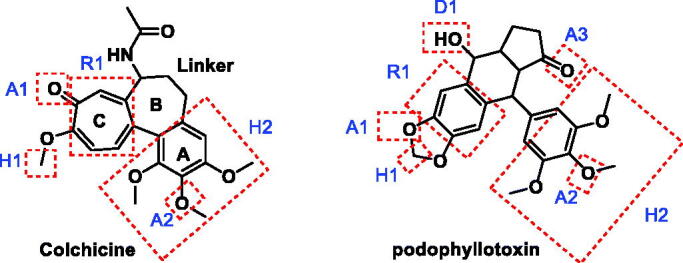
Pharmacophoric points of colchicine and podophyllotoxin as CBSIs.

Colchicine as a prototype of CBSIs is formed of three rings (A, B (linker), and C). some reports revealed that A- and C-ring encompasses the least pharmacophoric features required for binding to tubulin^45^. In addition, it was found that changes in the ring B (linker) could affect the antiproliferative activity the CBSIs^31^. Figure 4 shows the pharmacophoric points on colchicine and podophyllotoxin as representative examples of CBSIs.

**Figure 5.**
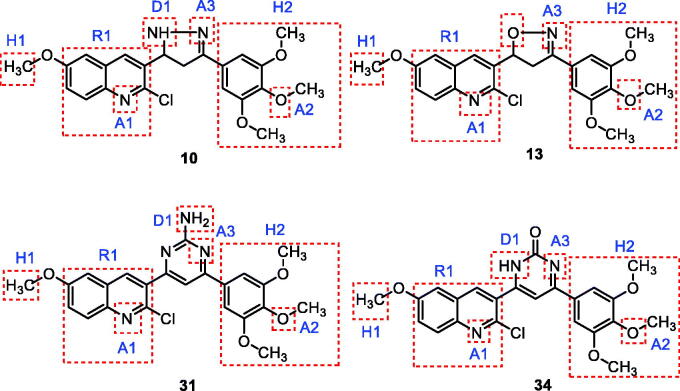
Representative examples of the newly synthesised compounds having the same essential pharmacophoric features of the CBSIs.

**Figure 6.**
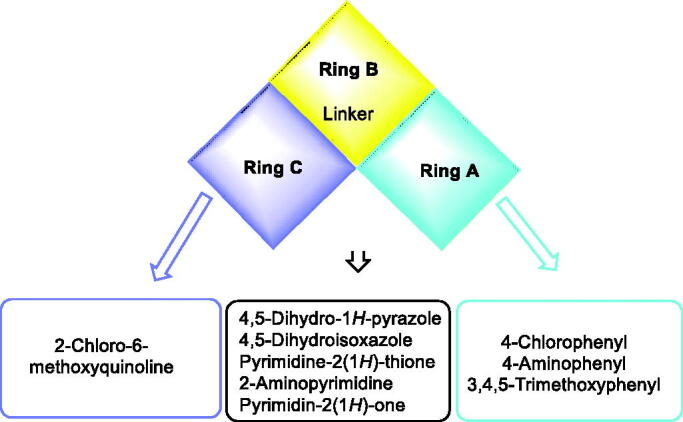
Summary for all chemical modifications.

The main target of this work was the synthesis of new quinoline derivatives having the same essential pharmacophoric features of the reported CBSIs (Figure 5). The core of our molecular design rational comprised bioisosteric modification strategies of CBSIs at three different positions (Figure 6).

The first position was the C-ring, where the 2-chloro-6-methoxyquinoline moiety used as a bioisosteric ring equivalent. The second position was the B-ring (linker region), where different hetero-rings were used including five-membered rings as 4,5-dihydro-1*H*-pyrazole (compounds **8 − 10** and **14 − 25**) and 4,5-dihydroisoxazol (compounds **11 − 13**), or six-membered rings as pyrimidine-2(1*H*)-thione (compounds **26 − 28**), 2-aminopyrimidine (compounds **29 − 31**), and pyrimidin-2(1*H*)-one (compounds **32 − 34**). The third position was the A-ring, where different substituted phenyl rings were used including 4-chlorophenyl, 4-aminophenyl, and 3,4,5-trimethoxyphenyl.

**Figure 7.**
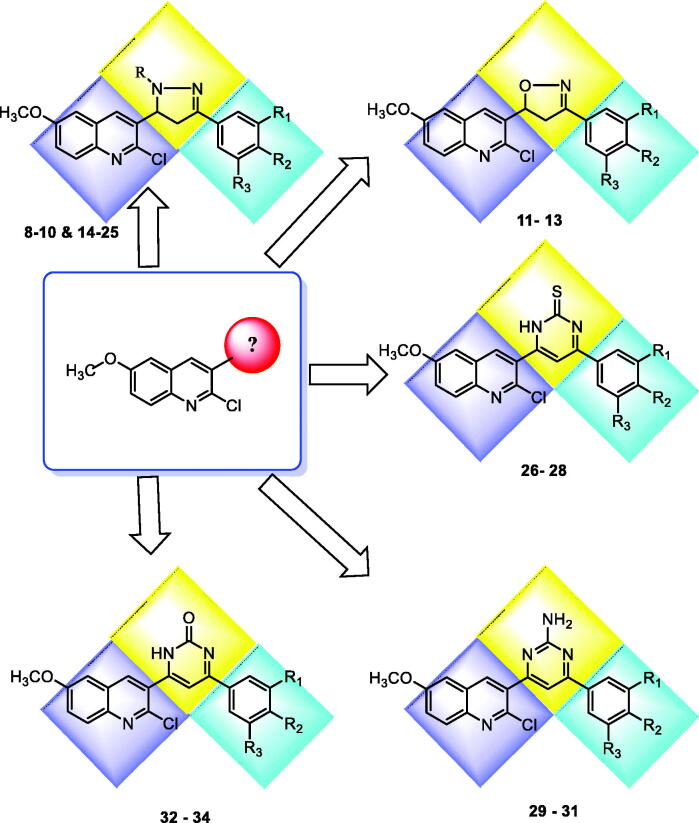
Rational of molecular design of the new proposed CBSIs.

The wide variety of modifications enabled us to study the SAR of these compounds as effective anti-cancer agents with potential tubulin inhibitory activity which is considered as a crucial objective of our work. All modification pathways and molecular design rationale were illustrated and summarised in Figure 7.

## 2. Results and discussion

### 2.1. Chemistry

**Scheme 1. Synthesis of the target compounds 5–7.**
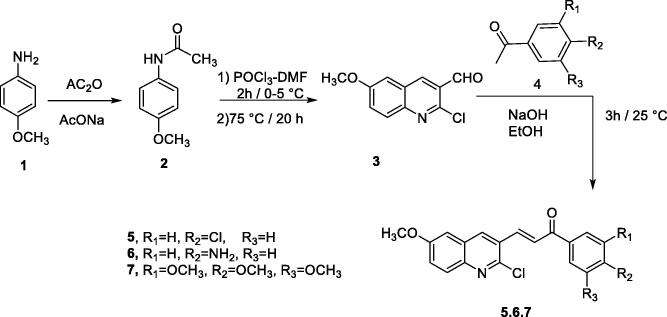


**Scheme 2. Synthesis of the target compounds 8–25.**
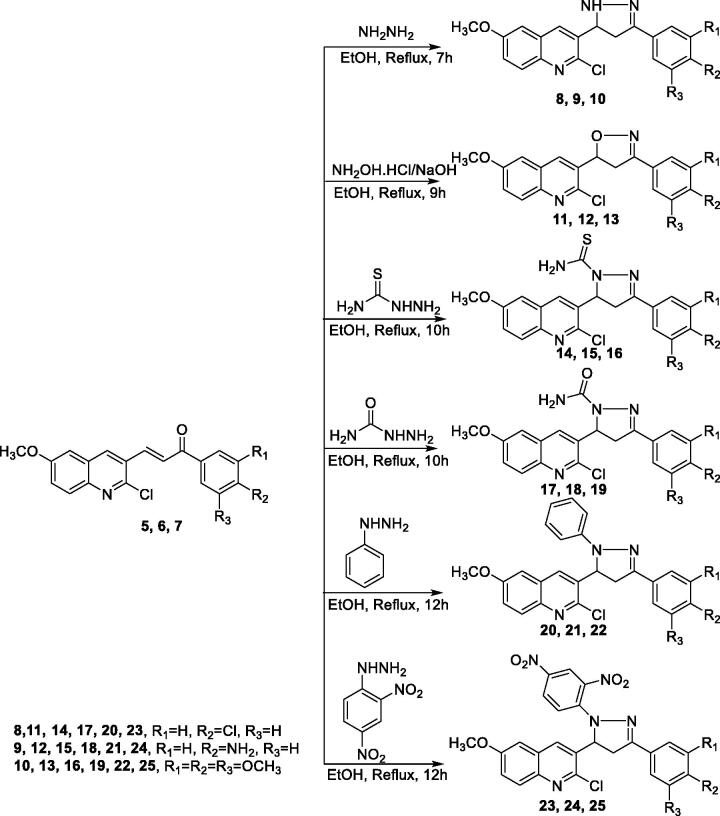


**Scheme 3. Synthesis of the target compounds 26–34.**
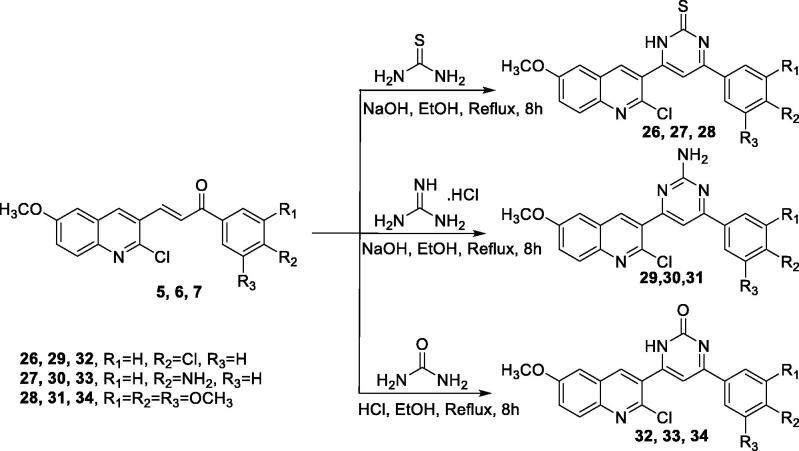


The synthetic pathways employed to prepare the target compounds are outlined in Schemes 1–3. Firstly, *p*-aminoacetophenone **1** was refluxed in acetic anhydride/sodium acetate mixture to produce *N*-(4-methoxyphenyl) acetamide **2.** Boiling compound **2** with POCl_3_ in the presence of catalytic amount of DMF, produced aldehyde derivative **3**. Chalcone derivatives **5, 6,** and **7** were prepared through the reaction of compound **3** with the corresponding acetophenones **4** in the presence of alcoholic NaOH. The reaction occurred by ratio (1:1) to form chalcone which crystallised from ethanol, the structure of compounds **5, 6, and 7** were established on the basis of its elemental and spectral data. The IR spectra of compounds **5, 6,** and **7** were characterised by strong absorption bands around 1655 cm^−1^ due to carbonyl ketone stretching, which appeared at low absorption value because of extended conjugation with the double bond. The absolute geometry of the *α,β*-unsaturated carbonyl linker was assigned to be in *trans* form based on the coupling constant alkene protons. In more details, the ^1^H NMR spectrum of chalcone **5** showed two doublets, each equivalent to one proton, at *δ* 8.1 ppm due to CH alkene (β-proton) and at *δ* 7.9 ppm due to CH alkene (α-proton). Both protons have the same coupling constant value of 15.2 Hz, which confirms the *E*-configuration. Additionally, the ^1^H NMR spectrum of compound **5** displayed two more doublets, each of two protons, at *δ* 8.2 and 7.1 ppm, with coupling constant value of 8.4 Hz which is attributed to the *p*-disubstituted phenyl moiety (Scheme 1, Supplementary data).

The reactivity of chalcone can attributed to the fact that their molecules have two electron poor centres at C-1 and C-3 in addition to one electron rich centre at C-2, so the double bond in chalcone can be looked on as an electron rich bond that may enter in 1,3-dipolar cycloaddition reactions. Chalcone are heavily utilised synthons in 1,3-cycloadditon reactions to build a wide variety of heterocyclic systems such as pyrrolidines, oxazolines and pyrimidines. In this work, nine different 1,3-dipolar cyclo-addition reactions have been achieved. all reactions proceeded smoothly and final products were obtained in relatively good yields as detailed in the experimental part. The subsequent paragraphs point out those reactions in depth (Scheme 2).

First, to build a dihydroprazole ring system, a mixture of the chalcone **(5, 6,** and **7)** with excess hydrazine hydrate in absolute ethanol was heated up at reflux temperature to finally give the desired corresponding compounds **8, 9,** and **10**. The structure of compound **8** was established on the basis of its elemental and spectral data. IR spectrum of compound **8** exhibited an important band at 3295 cm^−1^ due to NH stretching of the newly formed dihydropyrazole ring. The later NH was also appeared on the ^1^H NMR spectrum as a broad deuterium-exchangeable singlet within the aromatic region at *δ* 7.1 ppm. The hydrocarbon backbone of the newly built dihydropyrazole ring revealed three more signals; i.e. a triplet, of one proton at *δ* 5.1 ppm due to pyrazole-H5 that appeared downfield, as expected, because of the neighbouring nitrogen atom; on the other hand, there were two doublet of doublets, each of one proton at *δ* 3.7 and 2.8 ppm with coupling constants of 5.6 and 16.8 Hz assignable for pyrazole-H4 axial proton, 9.6 and 16.4 Hz of pyrazol-H4 equatorial proton. In addition, mass spectrum of compound **8** showed a strong base peak with the proper chlorine-isotope distribution pattern (see experimental part). Formation of dihydropyrazole derivatives were postulated to pass through two steps. The first step involves Michael-type addition on carbonyl *β*-carbon, followed by protonation to afford *β*-hydrazinylpropanone intermediate. The terminal primary amine attacks the carbonyl group to finally give the dihydropyrazole final product after loss a molecule of water as detailed below (Supplementary data).

Next, to synthesise dihydroisoxazole derivatives **11, 12,** and **13**, a mixture of chalcone (**5,6,7**), hydroxylamine hydrochloride, and NaOH in ethanol was heated up till reflux to give the corresponding target compounds **11,12,** and **13**. The IR spectra of compound **11** characterised by a band at 1590 cm^−1^ due to C=N, ^1^H NMR spectra of compound **11** showed, a doublet of doublet of one proton at *δ* 6 ppm due to isoxazole-H-5 which appeared downfield as expected because C-5 of isoxazole attached to oxygen atom, a two doublet of doublet, each equivalent for one proton at *δ* 4.0 and 3.5 ppm which attributed to isoxazole-H4 axial proton and isoxazole-H4 equatorial proton with coupling constant (11.2, 16.8 Hz) of axial proton and (6.8, 17.2 Hz) of equatorial proton, respectively, the most characteristic feature of ^1^H NMR spectrum of compound **11** is the disappearance of the olefinic protons (Supplementary data).

Next, to prepare pyrimidine-2(1*H*)-thione derivatives **26,27,** and **28**, a mixture of chalcone, thiourea, and NaOH in ethanol was heated to reflux to give the corresponding target derivatives. Taking compound **26** as an example, the IR spectrum was characterised by a band at 3308 cm^−1^ assignable to one NH stretching, the ^1^H NMR spectrum shows, a singlet of one proton at 14.1 ppm due to NH which is D_2_O exchangeable, a singlet for one proton at *δ* 8.6 ppm due to quinoline-H4, a doublet for one proton at *δ* 8.3 ppm due to quinoline-H-8 with coupling constant 8.8 Hz, a doublet of two protons at *δ* 8.1 ppm due to phenyl-H2,H6 with coupling constant 8.4 Hz, a doublet for one proton at 7.8 ppm due to quinoline-H7 with coupling constant 8.8 Hz, a doublet of two protons at *δ* 7.5 ppm due to phenyl-H3,H5 with coupling constant 8.4 Hz, a singlet for one proton at 7.4 ppm due pyrimidine-H5, a singlet of one proton at *δ* 7.3 ppm due to quinoline-H5, a singlet of three protons at *δ* 3.8 ppm due to OCH_3_ of quinoline (Scheme 3).

Similarly, pyrimidin-2-amine was synthesised. Briefly, a mixture of chalcone, guanidine hydrochloride, and NaOH in ethanol was heated to reflux to give the corresponding compounds **29, 30,** and **31**. The structure of the isolated product was confirmed by spectral data, taking compound **29** as a representative example, the IR spectrum characterised by a band at 3320 and 3284 cm^−1^ due to NH_2_ stretching, the ^1^H NMR spectrum revealed a singlet for one proton at *δ* 8.4 ppm due to quinoline-H4, a doublet of one proton at *δ* 8.3 ppm due to quinoline-H8 with coupling constant 8.8 Hz, a doublet for two protons at *δ* 7.9 ppm due to phenyl-H2,H6 with coupling constant 8.4 Hz, a singlet of one proton at *δ* 7.5 ppm due to pyrimidine-H5, a singlet of one proton at 7.1 ppm due to quinoline-H5, a doublet of two protons at 7 ppm due to phenyl-H3,H5, a doublet for one proton at *δ* 6.7 ppm due to quinoline-H7 with coupling constant 8.8 Hz, a singlet for two protons at 5.4 ppm which attributed to NH_2_, D_2_O exchangable, a singlet of three protons at 3.7 ppm due to OCH_3_ of quinoline.

Unlike other reactions in this series, reaction of chalcone with urea was preceded first under basic conditions, but the yield was poor, therefore we shifted towards the acidic medium. In brief, compounds **32, 33,** and **34** were prepared, in a good yield, by allowing the corresponding chalcone to react with urea in the presence of conc. hydrochloric acid at a reflux temperature. Under strong acidic media, an alternative product was also anticipated, where the chlorine atom was expected to be hydrolysed to afford the amide-containing structure. Later structure was excluded mainly base on the absence its molecular ion peak from the MS. Other spectral and elemental data confirm this assumption and confirm the structure of compounds **32, 33,** and **34** (Supplementary data).

### 2.2. Biological evaluation

#### 2.2.1. *In vitro* anti-proliferative activity

*In vitro* cytotoxic activities of the synthesised compounds were assessed using standard MTT method^46–48^. a panel of human cancer cell lines namely; hepatocellular carcinoma (HepG-2), colorectal carcinoma (HCT-116), and breast cancer (MCF-7) were used in this test utilising colchicine as a reference standard. The antiproliferative activity was expressed in IC_50_ values as reported in Table 1.

**Table 1.** *In vitro* anti-proliferative activities of the tested compounds and colchicine

Comp.	IC_50_ ( µM )^a^		
	HepG-2	HCT-116	MCF-7
**5**	29.17 ± 1.9	28.92 ± 1.7	32.19 ± 2.0
**6**	32.91 ± 2.1	28.63 ± 1.7	37.31 ± 2.1
**7**	23.63 ± 1.3	25.08 ± 1.2	16.87 ± 1.2
**8**	30.92 ± 1.2	22.43 ± 1.1	33.53 ± 2.4
**9**	16.64 ± 0.8	17.40 ± 1.0	20.49 ± 1.1
**10**	15.38 ± 0.9	17.41 ± 1.1	25.12 ± 1.2
**11**	20.52 ± 1.0	19.72 ± 1.3	22.77 ± 1.4
**12**	35.44 ± 1.8	40.47 ± 2.4	45.53 ± 2.8
**13**	12.87 ± 0.6	9.96 ± 0.3	17.04 ± 1.2
**14**	8.92 ± 0.4	**7.62 ± 0.4**	**10.04 ± 0.4**
**15**	10.25 ± 0.4	11.56 ± 0.9	13.60 ± 0.7
**16**	9.30 ± 0.3	8.87 ± 0.3	13.89 ± 0.3
**17**	12.73 ± 0.5	11.42 ± 0.4	15.94 ± 0.6
**18**	10.18 ± 0.4	10.41 ± 0.3	13.70 ± 0.8
**19**	10.94 ± 0.5	**7.38 ± 0.4**	**9.04 ± 0.5**
**20**	**2.60 ± 0.1**	**1.81 ± 0.1**	**4.51 ± 0.2**
**21**	**7.32 ± 0.2**	**8.91 ± 0.3**	**9.19 ± 0.3**
**22**	**4.76 ± 0.2**	**3.04 ± 0.3**	**6.45 ± 0.2**
**23**	**2.64 ± 0.1**	**1.78 ± 0.1**	**4.48 ± 0.1**
**24**	**3.14 ± 0.1**	**4.18 ± 0.2**	**4.97 ± 0.1**
**25**	**1.89 ± 0.2**	**1.43 ± 0.1**	**4.21 ± 0.1**
**26**	**3.62 ± 0.1**	**2.24 ± 0.2**	**5.55 ± 0.2**
**27**	16.26 ± 0.4	22.36 ± 0.5	24.34 ± 0.9
**28**	**5.47 ± 0.2**	**5.85 ± 0.3**	**7.34 ± 0.2**
**29**	16.49 ± 0.8	13.04 ± 0.6	17.62 ± 0.8
**30**	28.53 ± 1.3	32.74 ± 0.9	33.75 ± 1.5
**31**	9.45 ± 0.5	10.49 ± 0.4	15.19 ± 0.7
**32**	8.16 ± 0.2	**6.48 ± 0.2**	**9.89 ± 0.4**
**33**	9.37 ± 0.4	**8.50 ± 0.2**	**10.27 ± 0.5**
**34**	15.27 ± 0.4	11.92 ± 0.4	15.44 ± 0.8
**Colchicine**	7.40 ± 0.2	9.32 ± 0.2	10.41 ± 0.3

^a^ IC_50_ values are the mean ± SD of three separate experiments. Bold figures indicate superior potency than colchicine and CA-4.

From the cytotoxic screening, the tested compounds showed different degrees of activities against the tested cells. In general, some compounds were proven to be efficient anticancer candidates with promising activities against the tested cells. Comparing to colchicine (IC_50_ = 7.40, 9.32, and 10.41 µM against HepG-2, HCT-116, and MCF-7, respectively), compounds **20**, **21**, **22**, **23**, **24**, **25**, **26,** and **28** exhibited superior cytotoxic activities with IC_50_ values ranging from 1.78 to 9.19 µM. Taking compound **25** as demonstrative example, it showed 3.91, 6.51 and 2.47 times of colchicine cytotoxic activity against HepG-2, HCT-116, and MCF-7, respectively. Also, compound **20** exhibited 2.84, 5.15 and 2.30 times of colchicine activity against HCT-116, HepG-2, and MCF-7, respectively. In addition, Compounds **14, 19, 32,** and **33** exhibited superior activities against HCT-116 and MCF-7 with IC_50_ values ranging from 6.48 to 10.27 µM.

Additionally, compounds **15**, **17**, **18**, **29,** and **34** displayed moderate anti-proliferative activities against all the tested cell lines with IC_50_ values ranging from 10.18 to 17.62 µM. On the other hand, compounds **5**, **6**, **8**, **12**, and **30** showed weak anti-proliferative activities against all cell lines with IC_50_ values ranging from 22.43 to 45.53 µM.

Finally, compound **16** showed strong activity against HCT-116, HepG-2 cell lines (IC_50_ = 9.30 and 8.87 µM, respectively) and moderated activity against MCF-7 cells (IC_50_ = 13.89 µM). Compound **13** showed strong activity against HepG-2 cell line (IC_50_ = 9.96 µM) and moderated activity against HCT-116 and MCF-7 cells (IC_50_ = 12.87 and 17.04 µM, respectively). Compounds **9** and **10** showed moderate activity against HCT-116 (IC_50_ = 16.64 and 15.38 µM, respectively), HepG-2 (IC_50_ = 17.40 and 17.41 µM, respectively) cell lines, and showed weak activity against MCF-7 cells (IC_50_ = 20.49 and 25.12 µM, respectively).

#### 2.2.2. Tubulin polymerisation assay

The most active antiproliferative members were further evaluated for their inhibitory effect against tubulin polymerisation. The inhibition assay on microtubule polymerisation was evaluated turbidimetrically using a fluorescent plate reader^49^. Colchicine and CA-4 were used as positive controls (Table 2).

**Table 2.** *In vitro* tubulin polymerisation inhibition.

Comp.	IC_50_ (nM)^a^ Tubulin polymerisation inhibition	Comp.	IC_50_ (nM)^a^ Tubulin polymerisation inhibition
**14**	50.1	**26**	34.5
**20**	36.8	**28**	37.4
**21**	9.11	**32**	10.5
**22**	19.1	**33**	47.2
**24**	146.2	Colchicine	10.6
**25**	41.3	CA-4	13.2

^a^ IC_50_ values are the mean ± SD of three separate experiments.

Compounds **21** and **32** exhibited the highest tubulin polymerisation inhibitory effect with IC_50_ values of 9.11 and 10.5 nM, respectively. Such members showed activities higher than that of colchicine (IC_50_ = 10.6 nM) and CA-4 (IC_50_ = 13.2 nM). Moreover, compounds **20, 22, 25, 26, 28,** and **33** showed promising activities with IC_50_ values of 36.8, 19.1, 41.3, 34.5, 37.4 and 47.2 nM, respectively. Compounds **14** showed moderate activity with IC_50_ value of 50.1 nM. Finally, Compound **24** exhibited weak tubulin polymerisation inhibitory activity with IC_50_ value of 146.2 nM.

#### 2.2.3. Cell cycle analysis

The impact of the most promising compound **25** on cell cycle distribution was assessed. Such analysis gave a better insight into the effect of the synthesised compounds on cancer cell growth inhibition. HepG-2 cells were utilised in this test. The reported method described by Wand et al.^50^ was followed. HepG-2 cells were treated with compound **25** at a concentration equals its IC_50_ value (1.89 µM) for 24 h.

**Figure 8.**
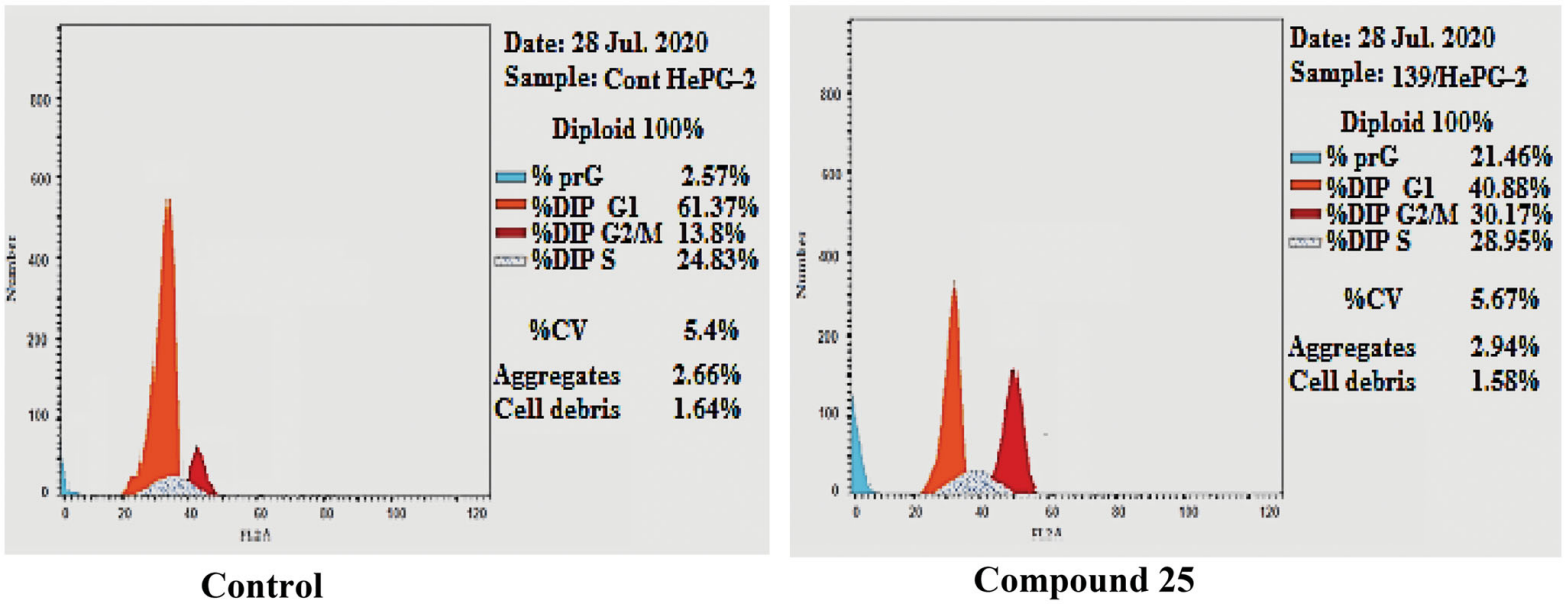
HepG-2 cells distribution upon treatment with compound **25**.

The results revelled that compound **25** induced an increase in the percentage of HepG-2 cells from 24.83% to 28.95% at S phase. It exhibited marked increase in the percentage of HepG-2 cells from 13.8% to 30.17% at G2/M phase. Also, it increased the percentage of HepG-2 cells from 2.57% to 21.46% at Pre-G1 phase. On the other hand, it decreased the percentage of HepG-2 cells from 61.37% to 40.88% at G0-G1phase. These findings indicated that compound **25** can arrest the cell cycle at G2/M phase, and can cause apoptosis at pre-G1 phase (Table 3 and Figure 8).

**Table 3.** Effect of compound **25** on cell cycle progression in HepG-2 cells.

Sample	Cell cycle distribution (%)			
	%G0-G1	%S	%G2-M	%Pre-G1
**25** / HepG-2	40.88	28.95	30.17	21.46
Cont. HepG-2	61.37	24.83	13.8	2.57

#### 2.2.4. Annexin V-FITC apoptosis assay

Annexin V and PI double staining assay^51^ was carried out to explore the proposed apoptotic effect of the synthesised compounds. Compound 25 as a representative example was tested against HepG-2 cells. In this test, HepG-2 cells were incubated with compound **25** at a concentration of 1.89 µM for 24 h.

**Figure 9.**
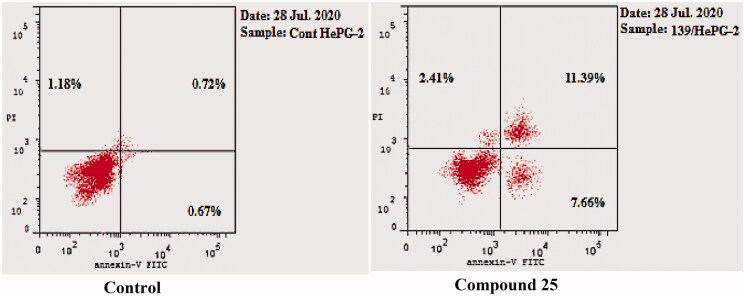
Induced apoptosis on HepG-2 cells by compound **25**.

As shown in Table 4 and Figure 9, compound **25** induced apoptotic effect equal 19.05% (7.66% and 11.39 at early and late apoptosis, respectively), which was thirteen times more than the control (1.39%).

**Table 4.** Apoptosis and necrosis percent induced by compound **25** in HepG-2 cells.

Sample	Apoptosis			Necrosis
	Total	Early	Late	
**25** / HepG-2	21.46	7.66	11.39	2.41
Cont. HepG-2	2.57	0.67	0.72	1.18

### 2.3. Docking studies

The synthesised compounds were docked against tubulin heterodimers using MOE2014 software to determine the binding free energy and binding mode (Table 5). Molecular docking studies gives perception about the binding mode and the degree of affinity between the docked compounds and the prospective target. good biological effect is shown by lower binding free energy and similar binding mode to that of the reference co-crystallised ligand.^52^

**Table 5.** The docking scores of the synthesised compounds, colchicine, and co-crystallised ligand (DAMA-colchicine) against tubulin

Comp.	Binding free energy (kcal/mol)	Comp.	Binding free energy (kcal/mol)
**5**	– 9.95	**21**	–11.67
**6**	–10.04	**22**	–14.72
**7**	–13.48	**23**	–13.42
**8**	–10.23	**24**	–14.35
**9**	–10.71	**25**	–17.12
**10**	–14.02	**26**	–11.23
**11**	–10.20	**27**	–11.25
**12**	–10.47	**28**	–12.74
**13**	–13.73	**29**	–11.18
**14**	–13.28	**30**	–11.11
**15**	–11.63	**31**	–14.65
**16**	–14.62	**32**	–11.05
**17**	–12.07	**33**	–11.02
**18**	–11.89	**34**	–13.14
**19**	–16.51	DAMA–colchicine	–12.88
**20**	–12.35	Colchicine	–13.45

**Figure 10.**
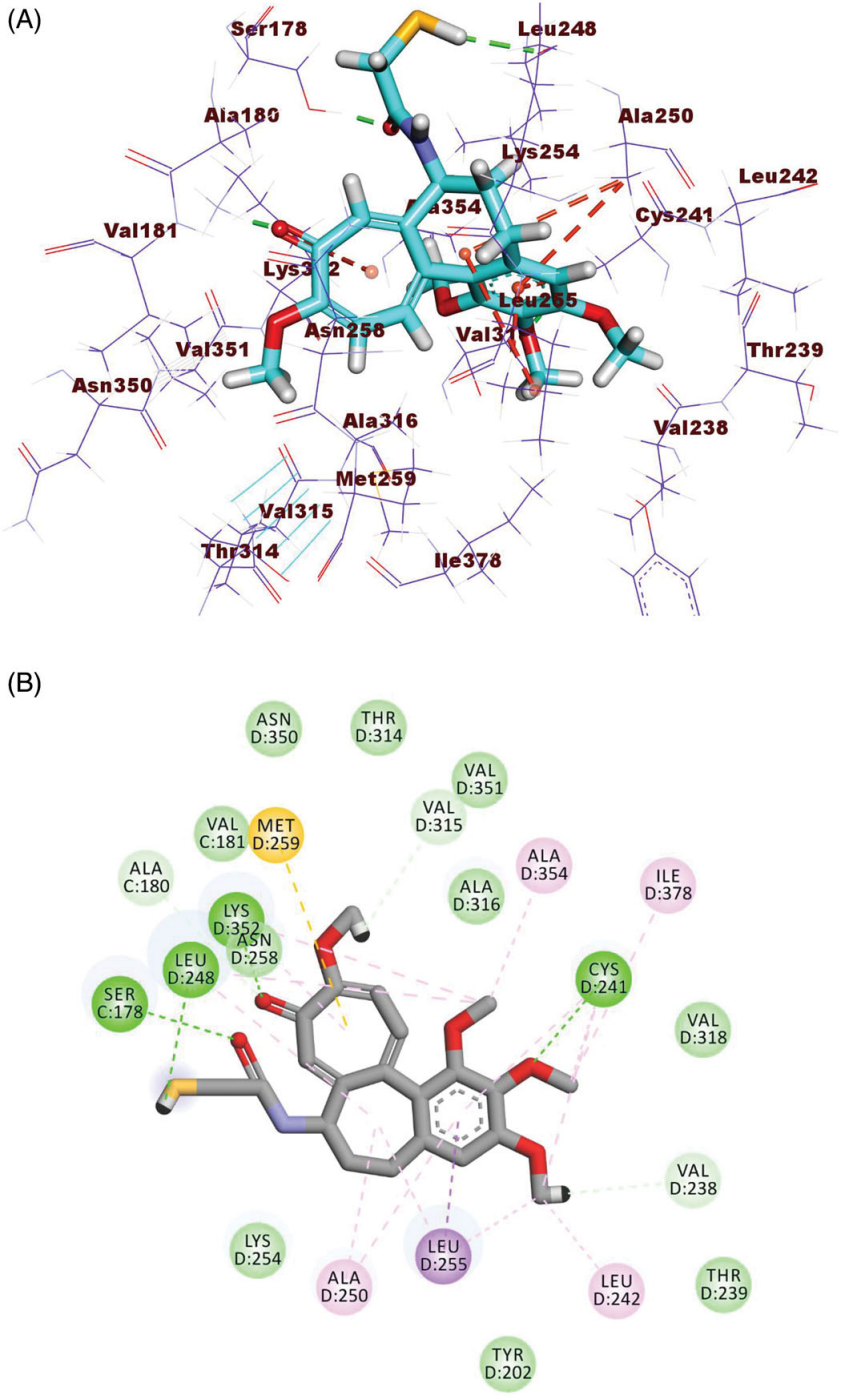
(A) 3D structure of co-crystallised ligand, DAMA-colchicine, docked into the colchicine binding site (B) 2D structure of co-crystallised ligand, DAMA-colchicine, docked into the colchicine binding site.

A docking study of the co-crystallised ligand, DAMA-colchicine provides a binding energy value of −12.88 Kcal/mol with four hydrogen bonds and six hydrophobic interactions. The trimethoxy phenyl (A-ring) ring occupied the first pocket of the colchicine binding site forming one hydrogen bond with Cys241. Other interactions were observed between the ring-A and different essential amino acid residues as Cys241, Leu255, and Ala250. Additionally, the side chain of the B-ring (2-mercaptoacetamide moiety) occupied the second cavity of the colchicine binding site forming two hydrogen bonds with Ser178 and Leu248. Furthermore, the methoxytropone moiety (C-ring) occupied the third pocket of the receptor forming one hydrogen and one hydrophobic bonds with Lys352 (Figure 10).

**Figure 11.**
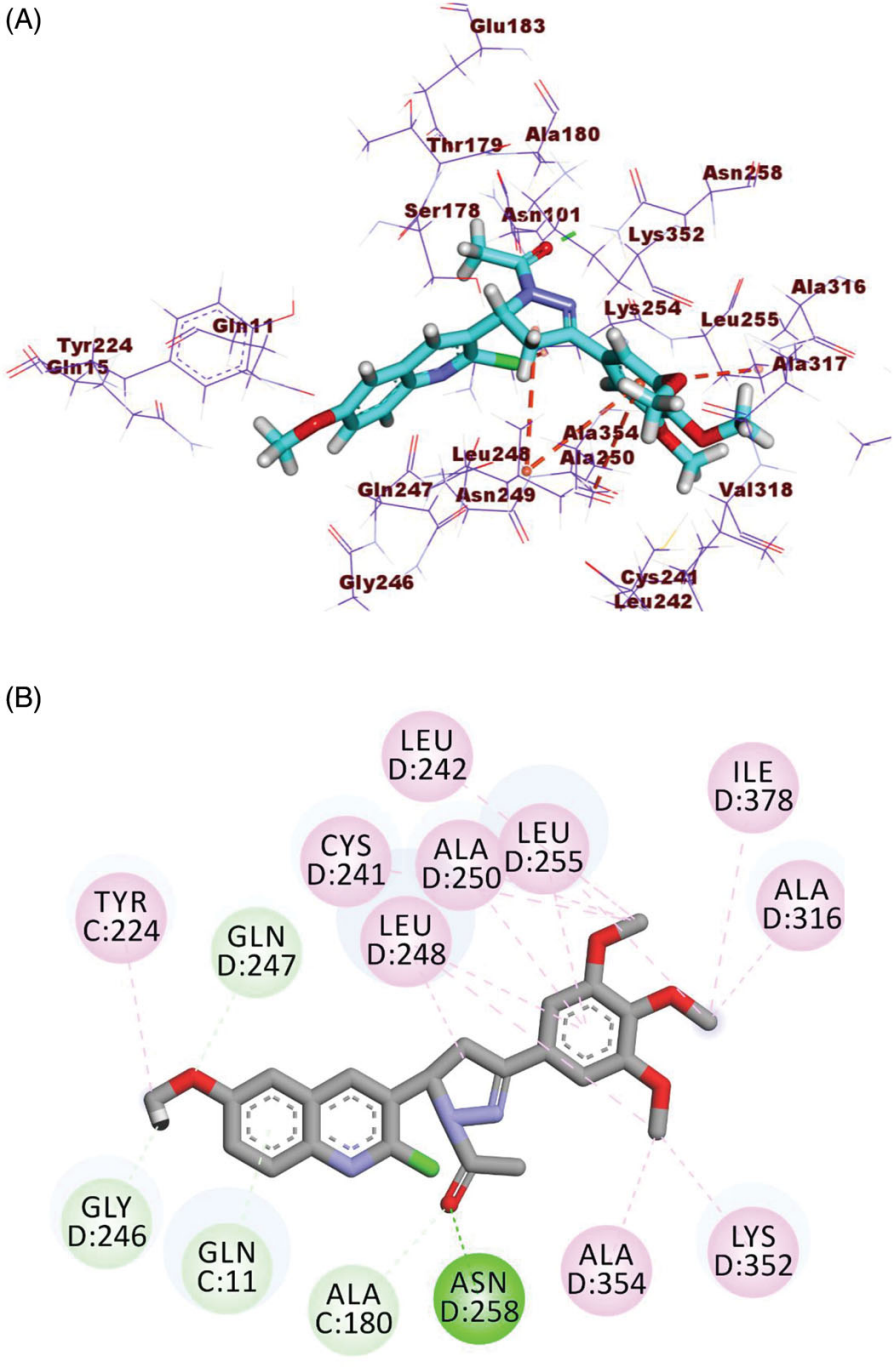
(A) 3D structure of compound **19** docked into the colchicine binding site (B) 2D structure of compound **19** docked into the colchicine binding site.

Compound **19** as a representative example showed a binding mode like that of DAMA-colchicine, with affinity value of −16.51 kcal/mol. The 3,4,5-trimethoxyphenyl moiety (A-ring) occupied the first cavity of the colchicine binding site forming three hydrophobic interactions with Ala250, Leu255, and Leu248. Additionally, the 2-acetyl-4,5-dihydro-1*H*-pyrazol moiety (B-ring) occupied the second cavity of the colchicine binding site forming one hydrogen bond with Asn258 and one hydrophobic interaction with Leu248. Furthermore, the 2-chloro-6-methoxyquinoline moiety (C-ring) occupied the third pocket of the colchicine binding site (Figure 11).

**Figure 12.**
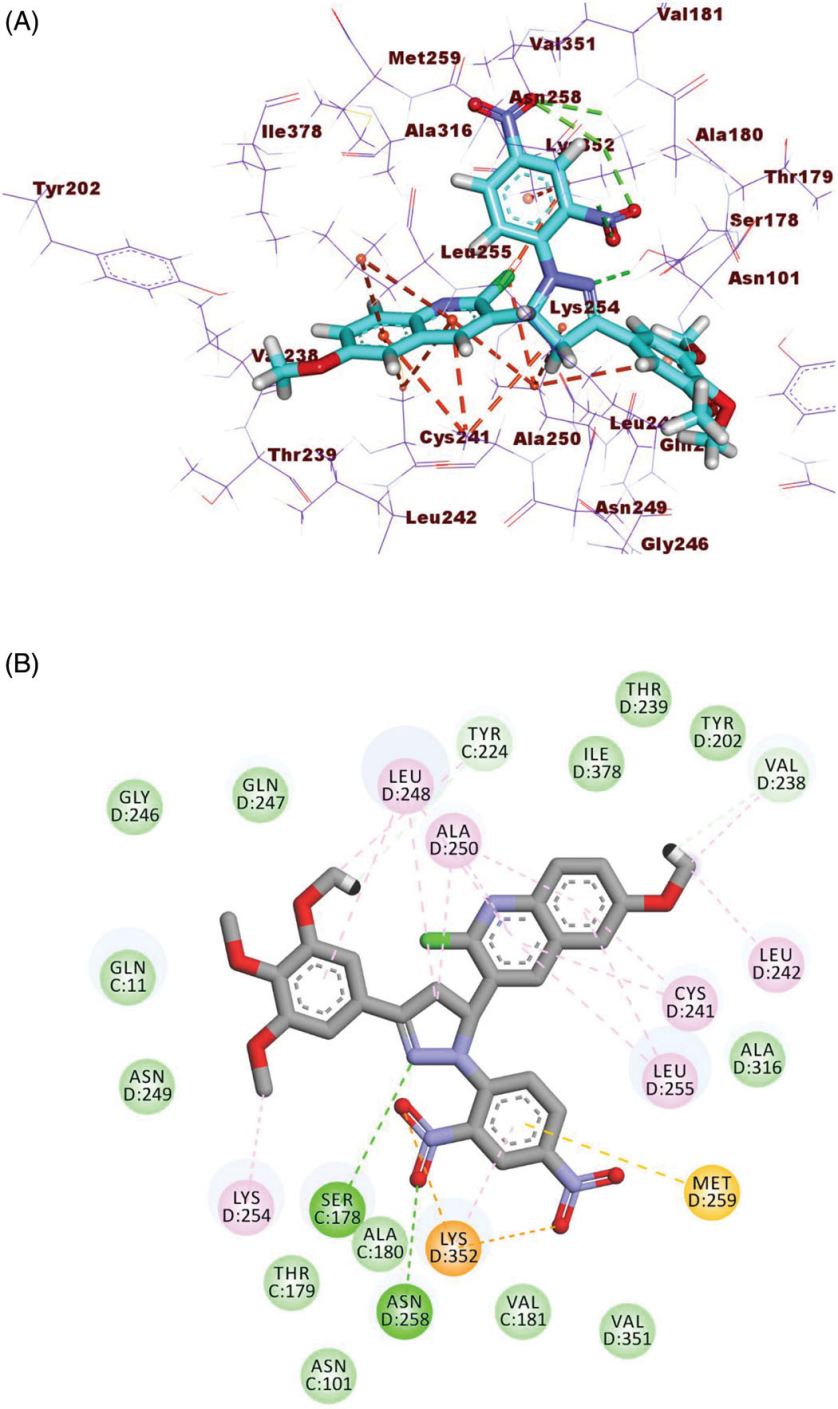
(A) 3D structure of compound **25** docked into the colchicine binding site (B) 2D structure of compound **25** docked into the colchicine binding site.

The binding mode of compound **25** exhibited an affinity value of −17.12 kcal/mol. The 3,4,5-trimethoxyphenyl moiety (A-ring) occupied the first cavity of the colchicine binding site forming one hydrophobic interaction with Leu248. The linker moiety (1–(2,4-dinitrophenyl)-4,5-dihydro-1*H*-pyrazole) occupied the second cavity of the colchicine binding site forming five hydrogen bonds with Lys352, Ser178, and Asn258. Also, it formed three hydrophobic interactions with Lys352, Ala250 and Leu248. Furthermore, the 2-chloro-6-methoxyquinoline moiety (C-ring) occupied the third pocket of the colchicine binding site forming eight hydrophobic interactions with Leu255, Cys241, and Leu248 (Figure 12).

**Figure 13.**
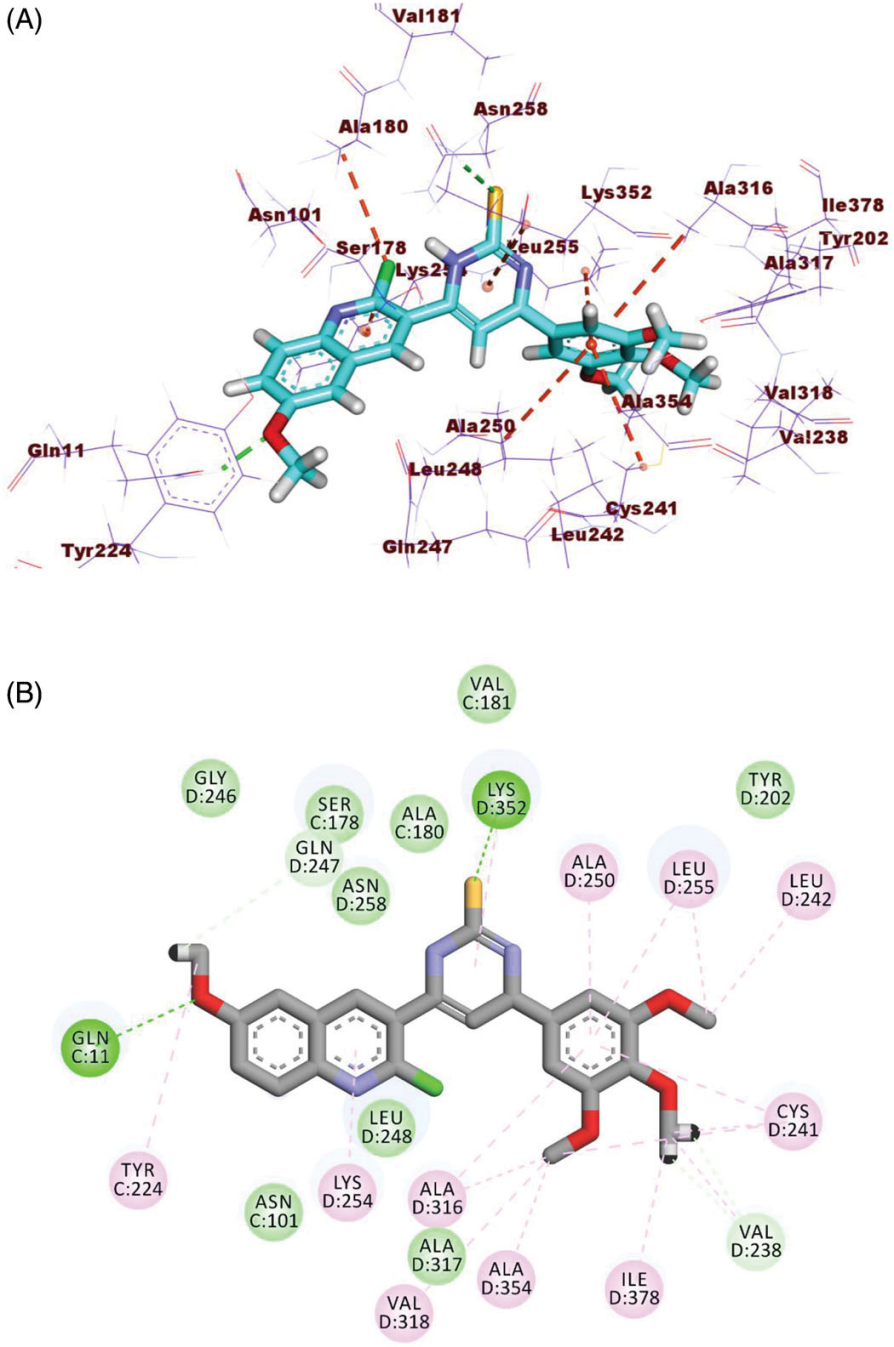
(A) 3D structure of compound **28** docked into the colchicine binding site (B) 2D structure of compound **28** docked into the colchicine binding site.

The binding mode of compound **28** exhibited an affinity value of −12.74 kcal/mol. The 3,4,5-trimethoxyphenyl moiety (A-ring) occupied the first cavity of the colchicine binding site forming four hydrophobic interactions with Ala316, Ala250, Cys241, and Leu255. The pyrimidine-2(1*H*)-thione (B-ring) occupied the second cavity of the colchicine binding site forming one hydrogen one hydrophobic bonds with Lys352. Furthermore, the 2-chloro-6-methoxyquinoline moiety (C-ring) occupied the third pocket of the colchicine binding site forming three hydrophobic interactions with Lys254 and Ala180. The methoxy group formed one hydrogen bond with Gln11 (Figure 13).

### 2.4. Structure-activity relationship (SAR)

SAR as an aim of our work was based on the results of *in vitro* cytotoxic activities of the synthesised compounds. Initially, the effect of the A-ring on the activity was explored. Comparing the cytotoxic activity of compounds **22, 25, 28, 31,** and **34** incorporating 4-methoxyphenyl as an A-ring with compounds **20, 23, 26, 29,** and **32** incorporating 4-chlorophenyl as an A-ring, and compound **21, 24, 27, 30,** and **32** incorporating 4-aminophenyl as an A-ring indicated that the cytotoxic activities decreased in the order of 4-methoxyphenyl > 4-chlorophenyl > 4-aminophenyl.

Then, the impact of the B-ring (linker region) was investigated. The increased IC_50_ values of compounds **5, 6,** and **7** incorporated open structure at the linker region than those of their corresponding members incorporating cyclic structure, indicated that cyclisation of the linker region (B-ring) is advantageous. For the B-ring, the cytotoxic activities decreased in the order of substituted five-membered ring (compounds **14–25**) > non-substituted six-membered ring (compounds **26–34**) > non-substituted five-membered ring (compounds **8–13**). With regard to the substituted five-membered ring, it was found that the cytotoxicity decrease in the order of 2,4-dinitrophenyl (compounds **23–25**) > phenyl (compounds **20–22**) > ethane-1-thione (compounds **14–16**) > ethan-1-one (compounds **17–19**).

**Figure 14.**
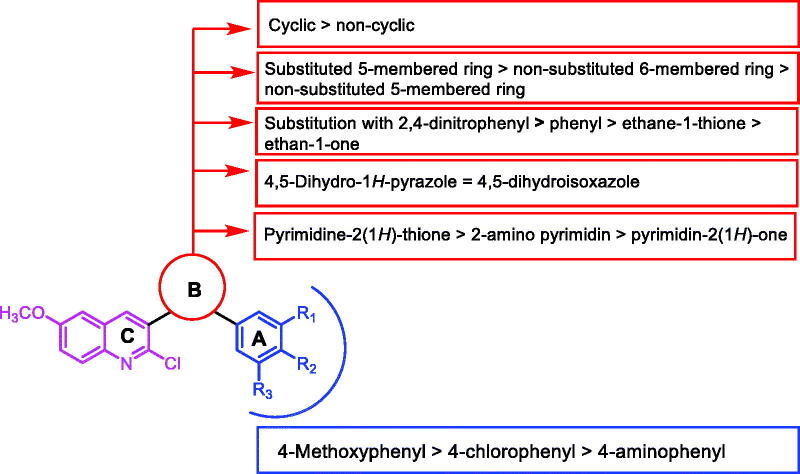
Structure-activity relationship of the synthesised compounds as anti-proliferative agents.

For the non-substituted five-membered ring, it was found 4,5-dihydro-1*H*-pyrazole derivatives (compounds **8–9**) are almost equipotent with 4,5-dihydroisoxazole ones (compounds **11–13**). For the non-substituted six-membered ring, it was found pyrimidine-2(1*H*)-thione derivatives (compounds **26–28**) are more active than 2-amino pyrimidine derivatives (compounds **29–31**), which were more active than pyrimidin-2(1H)-one derivatives (compounds **32–34**) (Figure 14).

## 3. Conclusion

In summary, twenty-nine of quinoline derivatives were designed and synthesised. The synthesised compounds were evaluated for their anti-proliferative activities against a group of three human cancer cell lines including; colorectal carcinoma (HCT-116), hepatocellular carcinoma (HepG-2), and breast cancer (MCF-7). Compounds **20**, **21**, **22, 23, 24, 25, 26,** and **28** exhibited superior cytotoxic activities with IC_50_ values ranging from 1.78 to 9.19 µM. Additionally, the most promising members were tested for their tubulin polymerisation inhibitory effect. Compounds **21** and **32** exhibited the highest tubulin polymerisation inhibitory effect with IC_50_ values of 9.11 and 10.5 nM, respectively. Structure-activity relationship of the synthesised compounds revealed that 4-methoxyphenyl > 4-chlorophenyl > 4-aminophenyl as an A-ring. In the same time. The it was found that substituted five-membered ring > non-substituted six-membered ring > non-substituted five-membered ring as a B-ring. Moreover, compound **25** was approved to arrested the cell cycle at the G2/M phase and induced apoptosis in HepG-2 cells. Docking experiments assisted these findings by anticipating potential binding interactions between the target compounds and the active sites of tubulin heterodimers. The most effective candidates in the quest for strong and selective antineoplastic agents will serve as valuable lead compounds and merit further investigations.

## 4. Experimental

### 4.1. Chemistry

^1^H NMR spectra were run at 400 MHz and ^13 ^C spectra were determined at 100 MHz in deuterated chloroform (CDCl_3_), or dimethyl sulfoxide (DMSO-*d_6_*) on a Varian Mercury VX-400 NMR spectrometer. Chemical shifts are given in parts per million (ppm) on the delta (δ) scale. Chemical shifts were calibrated relative to those of the solvents. Flash chromatography was performed on 230–400 mesh silica. The progress of reactions was monitored with Merck silica gel IB2-F plates (0.25 mm thickness). The infra-red spectra were recorded in potassium bromide discs on pye Unicam SP 3300 and Shimadzu FT IR 8101 PC infra-red spectrophotometer. Mass spectra were recorded at 70 eV. High resolution mass spectra for all ionisation techniques were obtained from a Finnigan MAT XL95. Melting points were determined using capillary tubes with a Stuart SMP30 apparatus and are uncorrected. All yields reported refer to isolated yields.

#### 4.1.1. General procedure for synthesis of compounds (5, 6, and 7)

To a stirred and ice-cooled aqueous solution of sodium hydroxide (10 mmole, 50% w/w) and absolute ethanol (12.5 mL), substituted acetophenone (10 mmole) namely, 4-chloroacetophenoe, 4-aminoacetophenone, and 3,4,5-trimethoxyacetophenone was added followed by 2-chloro-6-methoxyquinoline-3-carbadehyde **4** (10 mmole). The reaction mixture was vigorously stirred for 3–6 h while temperature was maintained below 25 °C till the reaction mixture became thick. The reaction mixture was left in the refrigerator overnight. The formed precipitate was filtered off under vaccum and washed with copious amount of water until the filtrates became neutral to litmus paper, washed with ice-cold ethanol (20 mL), and then recrystallized from ethanol to give the titled compound **5, 6,** and **7,** respectively.

##### 4.1.1.1. (E)-3–(2-Chloro-6-methoxyquinolin-3-yl)-1–(4-chlorophenyl)prop-2-en-1-one (5)

Yellow solid (93%) mp = 135–136 °C; IR (KBr) cm^−1^: 3080 (CH aromatic), 2900 (CH aliphatic), 1655 (C=O); ^1^H NMR (DMSO-*d_6_*) δ: 9.10 (s, 1H, quinoline-H4), 8.20 (d, *J* = 8.4 Hz, 2H, phenyl-H2, H6 protons), 8.13 (d, *J* = 15.2, 1H, CH alkene β proton), 7.96 (d, *J* = 15.2 Hz, 1H, CH alkene α proton), 7.92 (d, *J* = 9.2, 1H, quinoline-H8), 7.50 (d, *J* = 9.2 Hz, 1H, quinoline-H7), 7.39 (s, 1H, quinoline-H5), 7.14 (d, *J* = 8.4, 2H, phenyl-H3, H5 protons), 3.87 (s, 3H, quinoline-OCH_3_); MS (*m/z*) 361 (0.8%, M + 4), 359 (4.3%, M + 2), 357 (6.7%, M^+^), 246 (61%), 138 (100%); Anal. Calc. for: (C_19_H_13_Cl_2_NO_2_): C, 63.71; H, 3.66; N, 3.91%; Found: C, 63.74; H, 3.71; N, 3.97%.

##### 4.1.1.2. (E)-1–(4-Aminophenyl)-3–(2-chloro-6-methoxyquinolin-3-yl)prop-2-en-1-one (6)

Orange solid (89%) mp = 120–121 °C; IR (KBr) cm^−1^: 3310, 3295 (NH_2_), 3054 (CH aromatic), 2962 (CH aliphatic), 1653 (C=O); ^1^H NMR (DMSO-*d_6_*) δ: 9.08 (s, 1H, quinoline-H4), 8.07 (d, *J* = 15.2 Hz, 1H, CH alkene β proton), 7.96 (d, *J* = 8.8, 2H, phenyl-H2, H6 protons), 7.92 (d, *J* = 15.2 Hz, 1H, CH alkene α proton), 7.88 (d, *J* = 9.2, 1H, quinoline-H8), 7.49 (d, *J* = 9.2 Hz, 1H, quinoline-H7), 7.38 (s, 1H quinoline-H5), 6.64 (d, *J* = 8.8, 2H, phenyl-H3, H5 protons), 6.25 (brs, 2H, NH_2_, D_2_O-exchangeable), 3.91 (s, 3H, quinoline-OCH_3_); MS (*m/z*) 340 (1.96%, M^+^ +2), 338 (5.8%, M^+^), 246 (66%), 120 (100%); Anal. Calc. for: (C_19_H_15_ClN_2_O_2_): C, 67.36; H, 4.46; N, 8.27%; Found: C, 67.41; H, 4.49; N, 8.31%.

##### 4.1.1.3. (E)-3–(2-Chloro-6-methoxyquinolin-3-yl)-1–(3,4,5-trimethoxyphenyl)prop-2-en-1-one (7)

Yellow solid (91%) mp = 127–128 °C; IR (KBr) cm^−1^: 3064 (CH aromatic), 2910 (CH aliphatic), 1655 (C=O); ^1^H NMR (DMSO-*d_6_*) δ: 9.12 (s, 1H, quinoline-H4), 8.16 (d, *J* = 15.3 Hz, 1H, CH alkene β proton), 8.02 (d, *J* = 15.3, 1H, CH alkene α proton), 7.91 (d, *J* = 9.3 Hz, 1H, quinoline-H8), 7.52 (d, *J* = 9.3 Hz, 1H, quinoline-H7), 7.41 (s, 1H, quinoline-H5), 7.14 (s, 2H, phenyl-H2, H6 protons), 3.94 (s, 6H, 2OCH_3_), 3.89 (s, 6H, 2OCH_3_); MS (*m/z*) 415 (2.7%, M^+^ +2), 413 (7.8%, M^+^), 246 (76%), 195 (100%); Anal. Calc. for: (C_22_H_20_ClNO_5_): C, 63.85; H, 4.87; N, 3.38%; Found: C, 63.92; H, 4.92; N, 3.40%.

#### 4.1.2. General procedure for synthesis of compounds (8–25)

A mixture of chalcone **5–7** (10 mmole) and the appropriate amine derivatives namely, hydrazine hydrate, hydroxylamine hydrochloride, thiosemicarbazide, semicarbazide, phenylhydrazine, 2,4-dinitrophenylhydrazine (10 mmole) were stirred in ethanol (20 mL), and then sodium hydroxide (0.8 g, 20 mmole), in case of hydroxylamine hydrochloride, was added. The reaction mixture was heated to reflux for 7–12 h, and then the solvent was evaporated under reduced pressure and poured into ice water. The obtained precipitate was filtered off, washed with copious amount of water and recrystallized from ethanol to afford the corresponding compounds **8–25**.

##### 4.1.2.1. 2-Chloro-3-[3–(4-chlorophenyl)-4,5-dihydro-1H-pyrazol-5-yl]-6-methoxyquinoline (8)

White solid (76%) mp = 118–119 °C; IR (KBr) cm^−1^: 3295 (NH), 3071 (CH aromatic), 2945 (CH aliphatic); ^1^H NMR (DMSO-*d_6_*) δ: 8.40 (s, 1H, quinoline-H4), 7.84 (d, *J* = 8.8 Hz, 2H, phenyl-H2, H6 protons), 7.62 (d, *J* = 9.6, 1H, quinoline-H8), 7.46 (s, 1H, quinoline-H5), 7.41 (d, *J* = 9.6 Hz, 1H, quinoline-H7), 7.01 (brs, 1H, NH, D_2_O-exchangeable), 6.94 (d, *J* = 8.8, 2H phenyl-H3, H5 protons), 5.13 (t, *J* = 10 Hz, 1H, pyrazole-H5), 3.75 (s, 3H, quinoline-OCH_3_), 3.70 (dd, *J* = 5.6, *J* = 16.8, 1H, pyrazole- H4 axial proton), 2.89 (dd, *J* = 9.6, *J* = 16.4, 1H, pyrazole-H4 equatorial proton); ^13 ^C NMR (DMSO-*d_6_*) δ: 159.4, 153.4, 147.9, 144.4, 142.3, 134.2, 133.2, 130.5, 129.3, 129.1, 128.1, 126.4, 123.8, 106.5, 55.9, 50.6, 46.1; MS (*m/z*) 375 (0.8%, M^+^ +4), 373 (4.3%, M + 2), 371 (6.7%, M^+^), 179 (61%), 192 (100%); Anal. Calc. for: (C_19_H_15_Cl_2_N_3_O): C, 61.31; H, 4.06; N, 11.29%; Found: C, 61.35; H, 4.11; N, 11.35%.

##### 4.1.2.2. 4-[5–(2-Chloro-6-methoxyquinolin-3-yl)-4,5-dihydro-1H-pyrazol-3-yl]aniline (9)

Yellow solid (81%) mp = 122–123 °C; 3305, 2984 (NH_2_), 3045 (CH aromatic), 2935 (CH aliphatic); ^1^H NMR (DMSO-*d_6_*) δ: 10.02 (brs, 1H, NH, D_2_O-exchangeable), 8.39 (s, 1H, quinoline-H4), 7.84 (d, *J* = 9.2 Hz, 1H, quinoline-H8), 7.48 (s, 1H, quinoline-H5), 7.45 (d, *J* = 9.2, 1H, quinoline-H7), 7.32 (d, *J* = 8.8 Hz, 2H, phenyl-H2, H6 protons), 6.55 (d, *J* = 8.8 Hz, 2H, phenyl-H3, H5 protons), 5.36 (brs, 2H, NH_2_, D_2_O-exchangeable), 5.04 (t, *J* = 10 Hz, 1H, pyrazole-H5), 3.85 (s, 3H, quinoline-OCH_3_), 3.57 (dd, *J* = 10.8 Hz, *J* = 16.4 Hz, 1H, pyrazole- H4 axial proton), 2.77 (dd, *J* = 10 Hz, *J* = 16.4 Hz, 1H, pyrazole-H4 equatorial proton); ^13 ^C NMR (DMSO-*d_6_*) δ: 159.4, 153.4, 150.3, 148.9, 142.3, 139.2, 135.7, 131.9, 129.4, 127.7, 127.1, 121.8, 116.6, 106.9, 55.2, 43.7, 36.7; MS (*m/z*) 354 (5.9%, M + 2), 352 (18%, M^+^), 260 (46%), 192 (100%); Anal. Calc. for: (C_19_H_17_ClN_4_O): C, 64.68; H, 4.86; N, 15.88%; Found: C, 64.73; H, 4.91; N, 15.92%.

##### 4.1.2.3. 2-Chloro-6-methoxy-3-[3–(3,4,5-trimethoxyphenyl)-4,5-dihydro-1H-pyrazol-5-yl]quinoline (10)

Yellow solid (77%) mp = 114–115 °C; 3295 (NH), 3047 (CH aromatic), 2944 (CH aliphatic); ^1^H NMR (DMSO-*d_6_*) δ: 8.41 (s, 1H, quinoline-H4), 7.86 (d, *J* = 9.2 Hz, 1H, quinoline-H8), 7.55 (s, 2H, phenyl-H2, H6 protons), 7.54 (brs, 1H, NH, D_2_O-exchangeable), 7.48 (s, 1H, quinoline-H5), 7.43 (d, *J* = 9.2 Hz, 1H, quinoline-H7), 5.14 (t, *J* = 9.2 Hz, 1H, pyrazole-H5), 3.87 (s, 6H, 2OCH_3_), 3.76 (s, 6H, 2OCH_3_), 3.66 (dd, *J* = 11.2 Hz, *J* = 16.4 Hz, 1H, pyrazole- H4 axial proton), 2.87 (dd, *J* = 9.2 Hz, *J* = 16.4 Hz, 1H, pyrazole-H4 equatorial proton); ^13 ^C NMR (DMSO-*d_6_*) δ: 157.9, 153.8, 153.4, 149.6, 144.7, 142.4, 136.8, 133.0, 128.1, 127.7, 126.4, 123.6, 115.3, 106.5, 56.9, 55.5, 55.1, 37.8, 29.9; MS (*m/z*) 429 (15%, M + 2), 427 (46%, M^+^), 192 (100%); Anal. Calc. for: (C_22_H_22_ClN_3_O_4_): C, 61.76; H, 5.18; N, 9.82%; Found: C, 61.82; H, 5.22; N, 9.84%.

##### 4.1.2.4. 5–(2-Chloro-6-methoxyquinolin-3-yl)-3–(4-chlorophenyl)-4,5-dihydroisoxazole (11)

Yellow solid (64%) mp = 145–146 °C; 3031 (CH aromatic), 2957 (CH aliphatic), 1590 (C = N); ^1^H NMR (DMSO-*d_6_*) δ: 8.31 (s, 1H, quinoline-H4), 7.88 (d, *J* = 8.8 Hz, 2H, phenyl-H2, H6 protons), 7.65 (d, *J* = 9.6, 1H, quinoline-H8), 7.51 (s, 1H, quinoline-H5), 7.43 (d, *J* = 9.6 Hz, 1H, quinoline-H7), 6.99 (d, *J* = 8.8, 2H, phenyl-H3, H5 protons), 6.02 (dd, *J* = 6.8 Hz, *J* = 11.2 Hz, 1H, isoxazole-H5), 4.08 (dd, *J* = 11.2, *J* = 16.8, 1H, isoxazole-H4 axial proton), 3.78 (s, 3H, quinoline OCH_3_), 3.51 (dd, *J* = 6.8, *J* = 17.2, 1H, isoxazole-H4 equatorial proton); ^13 ^C NMR (DMSO-*d_6_*) δ: 159.7, 153.4, 149.3, 145.1, 142.3, 134.3, 133.3, 130.5, 129.1, 128.1, 127.4, 126.0, 123.6, 106.2, 59.0, 55.5, 45.4; MS (*m/z*) 377 (1.8%, M + 4), 375 (12.3%, M + 2), 373 (18.9%, M^+^), 180 (100%); Anal. Calc. for: (C_19_H_14_Cl_2_N_2_O_2_): C, 61.14; H, 3.78; N, 7.51%; Found: C, 61.16; H, 3.81; N, 7.54%.

##### 4.1.2.5. 4-[5–(2-Chloro-6-methoxyquinolin-3-yl)-4,5-dihydroisoxazol-3-yl]aniline (12)

Orange solid (61%) mp = 127–128 °C; 3300, 3104 (NH_2_), 3041 (CH aromatic), 2959 (CH aliphatic); ^1^H NMR (DMSO-*d_6_*) δ: 8.26 (s, 1H, quinoline-H4), 7.84 (d, *J* = 9.2 Hz, 1H, quinoline-H8), 7.53 (s, 1H quinoline-H5), 7.42 (d, *J* = 9.2, 1H, quinoline-H7), 7.36 (d, *J* = 8.8 Hz, 2H, phenyl-H2, H6 protons), 6.55 (d, *J* = 8.8 Hz, 2H, phenyl-H3, H5 protons), 5.93 (t, *J* = 6 Hz, 1H, isoxazole-H5), 5.62 (brs, 2H, NH_2_, D_2_O-exchangeable), 3.96 (dd, *J* = 10.8 Hz, *J* = 17.2 Hz, 1H, isoxazole-H4 axial proton), 3.85 (s, 3H, quinoline OCH_3_), 3.38 (dd, *J* = 6.8 Hz, *J* = 17.2 Hz, 1H, isoxazole-H4 equatorial proton); ^13 ^C NMR (DMSO-*d_6_*) δ: 162.4, 159.0, 150.6, 148.6, 142.7, 139.5, 136.1, 129.4, 128.4, 127.7, 127.1, 121.8, 116.6, 106.5, 59.0, 55.5, 38.5; MS (*m/z*) 355 (7.3%, M^+^ +2), 353 (23.1%, M^+^), 161 (37%), 192 (100%); Anal. Calc. for: (C_19_H_16_ClN_3_O_2_): C, 64.50; H, 4.56; N, 11.88%; Found: C, 64.54; H, 4.59; N, 11.91%.

##### 4.1.2.5. 5–(2-Chloro-6-methoxyquinolin-3-yl)-3–(3,4,5-trimethoxyphenyl)-4,5-dihydroisoxazole (13)

Red solid (59%) mp = 133–134 °C; 3062 (CH aromatic), 2978 (CH aliphatic); ^1^H NMR (DMSO-*d_6_*) δ: 8.32 (s, 1H, quinoline-H4), 7.87 (d, *J* = 9 Hz, 1H, quinoline-H8), 7.67 (s, 2H, phenyl-H2, H6 protons), 7.53 (s, 1H, quinoline-H5), 7.44 (d, *J* = 9 Hz, 1H, quinoline-H8), 6.02 (t, *J* = 6.9 Hz, 1H, isoxazole-H5), 4.05 (dd, *J* = 5.2 Hz, *J* = 11.1 Hz, 1H, isoxazole-H4 axial proton), 3.87 (s, 6H, 2OCH_3_), 3.79 (s, 6H, 2OCH_3_), 3.52 (dd, *J* = 4.8 Hz, *J* = 17.4 Hz, 1H, isoxazole-H4 equatorial proton); ^13 ^C NMR (DMSO-*d_6_*) δ: 162.1, 157.9, 153.4, 149.3, 144.4, 142.3, 136.5, 133.0, 128.1, 127.7, 126.4, 123.2, 115.6, 106.5, 61.8, 56.9, 55.2, 54.8, 31.2; MS (*m/z*) 430 (12%, M^+^ +2), 428 (35%, M^+^), 192 (100%); Anal. Calc. for: (C_22_H_21_ClN_2_O_5_): C, 61.61; H, 4.94; N, 6.53%; Found: C, 61.66; H, 5.01; N, 6.54%.

##### 4.1.2.6. 5–(2-Chloro-6-methoxyquinolin-3-yl)-3–(4-chlorophenyl)-4,5-dihydro-1H-pyrazole-1-carbothioamide (14)

Yellow solid (65%) mp = 136–137 °C; 3310, 3249 (NH_2_), 3031 (CH aromatic), 2974 (CH aliphatic); ^1^H NMR (DMSO-*d_6_*) δ: 8.56 (s, 1H, quinoline-H4), 8.40 (d, *J* = 8.4 Hz, 2H, phenyl-H2, H6 protons), 8.10 (d, *J* = 9.4, 1H, quinoline-H8), 8.05 (s, 1H, quinoline-H5), 8.0 (brs, 2H, NH_2_, D_2_O-exchangeable), 7.95 (d, *J* = 9.2 Hz, 1H, quinoline-H7), 6.96 (d, *J* = 8.4, 2H, phenyl-H2, H6 protons), 4.57 (t, *J* = 7.6 Hz, 1H, pyrazole-H5), 3.75 (s, 3H, quinoline OCH_3_), 3.58 (dd, *J* = 12, *J* = 16.8, 1H, pyrazole- H4 axial proton), 2.93 (dd, *J* = 12, *J* = 18.2, 1H, pyrazole-H4 equatorial proton); ^13 ^C NMR (DMSO-*d_6_*) δ: 179.8, 159.4, 153.8, 147.9, 144.7, 142.7, 134.3, 133.0, 130.5, 129.2, 128.1, 127.4, 126.4, 123.6, 106.5, 62.4, 55.9, 37.1; MS (*m/z*) 435 (1.2%, M + 4), 433 (9%, M^+^ +2), 431 (13.2%, M^+^), 192 (100%); Anal. Calc. for: (C_20_H_16_Cl_2_N_4_OS): C, 55.69; H, 3.74; N, 12.99%; Found: C, 55.76; H, 3.82; N, 13.03%.

##### 4.1.2.7. 3–(4-Aminophenyl)-5–(2-chloro-6-methoxyquinolin-3-yl)-4,5-dihydro-1H-pyrazole-1-carbothioamide (15)

Orange solid (66%) mp = 123–124 °C; 3300, 3251 (NH_2_), 3037 (CH aromatic), 2898 (CH aliphatic); ^1^H NMR (DMSO-*d_6_*) δ: 8.22 (s, 1H, quinoline-H4), 7.83 (d, *J* = 9.2 Hz, 1H, quinoline-H8), 7.73 (brs, 2H, NH_2_, D_2_O-exchangeable), 7.54 (d, *J* = 8.8, 2H, phenyl-H2, H6 protons), 7.44 (s, 1H, quinoline-H5), 7.38 (d, *J* = 9.2 Hz, 1H, quinoline-H7), 6.55 (d, *J* = 8.8 Hz, 2H, phenyl-H3, H5 protons), 5.70 (brs, 2H, NH_2_, D_2_O-exchangeable), 5.14 (dd, *J* = 3.2 Hz, *J* = 11.2 Hz, 1H, pyrazole-H5), 3.94 (dd, *J* = 11.2 Hz, *J* = 18.4 Hz, 1H, pyrazole- H4 axial proton), 3.83 (s, 3H, quinoline OCH_3_), 3.19 (dd, *J* = 3.2 Hz, *J* = 17.6 Hz, 1H, pyrazole-H4 equatorial proton); ^13 ^C NMR (DMSO-*d_6_*) δ: 165.9, 159.3, 153.5, 150.6, 148.3, 142.3, 139.2, 137.1, 129.4, 128.1, 127.7, 126.7, 121.8, 116.4, 106.5, 64.9, 55.2, 36.8; MS (*m/z*) 413 (7.3%, M^+^ +2), 411 (22%, M^+^), 219 (33%), 192 (100%); Anal. Calc. for: (C_20_H_18_ClN_5_OS): C, 58.32; H, 4.40; N, 17.0%; Found: C, 58.35; H, 4.44; N, 17.03%.

##### 4.1.2.8. 5–(2-Chloro-6-methoxyquinolin-3-yl)-3–(3,4,5-trimethoxyphenyl)-4,5-dihydro-1H-pyrazole-1-carbothioamide (16)

Yellow solid (69%) mp = 121–122 °C; 3301, 3145 (NH_2_), 3064 (CH aromatic), 2987 (CH aliphatic); ^1^H NMR (DMSO-*d_6_*) δ: 9.44 (brs, 2H, NH_2_, D_2_O-exchangeable), 8.39 (s, 1H, quinoline-H4), 7.57 (d, *J* = 9.2 Hz, 1H, quinoline-H8), 7.57 (s, 2H, phenyl-H2, H6 protons), 7.44 (s, 1H, quinoline-H5), 7.44 (s, 1H, quinoline-H5), 6.94 (d, *J* = 9.2 Hz, 1H, quinoline-H7), 5.12 (t, *J* = 10 Hz, 1H, 1H, pyrazole-H5), 3.86 (s, 6H, 2OCH_3_), 3.75 (s, 6H, 2OCH_3_), 3.66 (dd, *J* = 10.8 Hz, *J* = 16.4 Hz, 1H, pyrazole- H4 axial proton), 2.84 (dd, *J* = 9.6 Hz, *J* = 16.8 Hz, 1H, pyrazole-H4 equatorial proton); ^13 ^C NMR (DMSO-*d_6_*) δ: 169.0, 157.9, 155.5, 153.1, 149.3, 144.1, 142.7, 136.4, 133.0, 127.7, 127.1, 125.6, 123.9, 120.5, 106.2, 62.4, 57.9, 55.2, 54.8, 36.0; MS (*m/z*) 488 (22%, M^+^ +2), 486 (65%, M^+^), 167 (100%); Anal. Calc. for: (C_23_H_23_ClN_4_O_4_S): C, 56.73; H, 4.76; N, 11.51%; Found: C, 56.80; H, 4.78; N, 11.53%.

##### 4.1.2.9. 5–(2-Chloro-6-methoxyquinolin-3-yl)-3–(4-chlorophenyl)-4,5-dihydro-1H-pyrazole-1-carboxamide (17)

White solid (58%) mp = 135–136 °C; IR (KBr) cm^−1^: 3294, 3195 (NH_2_), 3024 (CH aromatic), 2972 (CH aliphatic), 1681 (C=O); ^1^H NMR (DMSO-*d_6_*) δ: 8.57 (s, 1H, quinoline-H4), 8.51 (d, *J* = 5.2 Hz, 2H, phenyl-H2, H6 protons), 8.93–8.04 (m, 3H quinoline-H5, H7and H8) , 7.93 (brs, 2H, NH_2_, D_2_O-exchangeable), 7.19 (d, *J* = 5.2 Hz, 2H, phenyl-H3, H5 protons), 4.61 (t, *J* = 10 Hz, 1H, pyrazole-H5), 3.74 (s, 3H, quinoline OCH_3_), 3.57 (dd, *J* = 11.6, *J* = 19.6, 1H, pyrazole- H4 axial proton), 2.87 (dd, *J* = 8.4, *J* = 17.6, 1H, pyrazole-H4 equatorial proton); ^13 ^C NMR (DMSO-*d_6_*) δ: 166.3, 160.1, 153.5, 146.5, 144.4, 141.2, 134.3, 133.0, 130.5, 129.1, 128.1, 127.4, 126.0, 123.9, 106.2, 59.0, 55.9, 36.0; MS (*m/z*) 419 (2.2%, M + 4), 417 (14%, M^+^ +2), 415 (22.2%, M^+^), 192 (100%); Anal. Calc. for: (C_20_H_16_Cl_2_N_4_O_2_): C, 57.85; H, 3.88; N, 13.49%; Found: C, 57.89; H, 3.94; N, 13.55%.

##### 4.1.2.10. 3–(4-Aminophenyl)-5–(2-chloro-6-methoxyquinolin-3-yl)-4,5-dihydro-1H-pyrazole-1-carboxamide (18)

Yellow solid (73%) mp = 130–131 °C; IR (KBr) cm^−1^: 3298, 3193 (NH_2_), 3056 (CH aromatic), 2983 (CH aliphatic), 1680 (C=O); ^1^H NMR (DMSO-*d_6_*) δ: 8.23 (s, 1H, quinoline-H4), 7.84 (d, *J* = 9.2 Hz, 1H, quinoline-H8), 7.46 (d, *J* = 8.8, 2H, phenyl-H2, H6 protons), 7.41 (s, 1H, quinoline-H5), 7.38 (d, *J* = 9.2 Hz, 1H, quinoline-H7), 6.54 (d, *J* = 8.8 Hz, 2H, phenyl-H3, H5 protons), 6.48 (brs, 2H, NH_2_, D_2_O-exchangeable), 5.55 (brs, 2H, NH_2_, D_2_O-exchangeable), 5.35 (t, *J* = 10.4 Hz, 1H, pyrazole-H5), 3.88 (dd, *J* = 11.2 Hz, *J* = 16.8 Hz, 1H, pyrazole- H4 axial proton), 3.84 (s, 3H, quinoline OCH_3_), 3.14 (dd, *J* = 10 Hz, *J* = 12 Hz, 1H, pyrazole-H4 equatorial proton); ^13 ^C NMR (DMSO-*d_6_*) δ: 165.9, 159.4, 153.4, 151.0, 147.9, 142.7, 139.2, 137.1, 129.4, 128.4, 127.7, 126.7, 121.8, 116.3, 106.5, 64.2, 55.5, 37.1; MS (*m/z*) 397 (5.3%, M^+^ +2), 395 (16.7%, M^+^), 203 (27%), 192 (100%); Anal. Calc. for: (C_20_H_18_ClN_5_O_2_): C, 60.69; H, 4.58; N, 17.69%; Found: C, 60.76; H, 4.63; N, 17.74%.

##### 4.1.2.11. 5–(2-Chloro-6-methoxyquinolin-3-yl)-3–(3,4,5-trimethoxyphenyl)-4,5-dihydro-1H-pyrazole-1-carboxamide (19)

Yellow solid (63%) mp = 117–118 °C; IR (KBr) cm^−1^: 3317, 3290 (NH_2_), 3031 (CH aromatic), 2934 (CH aliphatic), 1678 (C=O); ^1^H NMR (DMSO-*d_6_*) δ: 8.37 (s, 1H, quinoline-H4), 7.83 (d, *J* = 9.3 Hz, 1H, quinoline-H8), 7.77 (s, 2H, phenyl-H2, H6 protons), 7.52 (d, *J* = 9.3 Hz, 1H, quinoline-H7), 7.37 (s, 1H quinoline-H5), 6.52 (brs, 2H, NH_2_, D_2_O-exchangeable), 5.14 (t, *J* = 6.6 Hz, 1H, pyrazole-H5), 3.91 (s, 6H, 2OCH_3_), 3.83 (s, 6H, 2OCH_3_), 3.72 (dd, *J* = 15.4 Hz, *J* = 7.2 Hz, 1H, pyrazole- H4 axial proton), 3.28 (dd, *J* = 13.5 Hz, *J* = 7.5 Hz, 1H, pyrazole-H4 equatorial proton); ^13 ^C NMR (DMSO-*d_6_*) δ: 166.6, 157.9, 153.8, 152.7, 149.3, 144.4, 142.3, 136.8, 133.3, 127.7, 127.1, 126.0, 123.2, 115.6, 106.5, 63.8, 56.9, 54.5, 54.2, 36.8; MS (*m/z*) 472 (25%, M^+^ +2), 470 (73%, M^+^), 192 (100%); Anal. Calc. for: (C_23_H_23_ClN_4_O_5_): C, 58.66; H, 4.92; N, 11.90%; Found: C, 58.69; H, 4.98; N, 11.94%.

##### 4.1.2.12. 2-Chloro-3-[3–(4-chlorophenyl)-1-phenyl-4,5-dihydro-1H-pyrazol-5-yl]-6-methoxyquinoline (20)

Yellow solid (69%) mp = 145–146 °C; IR (KBr) cm^−1^: 3024 (CH aromatic), 2951 (CH aliphatic); ^1^H NMR (DMSO-*d_6_*) δ: 8.57 (s, 1H, quinoline-H4), 8.41 (d, *J* = 8.4 Hz, 2H, chlorophenyl-H2, H6 protons), 8.10–7.94 (m, 4H, quinoline-H5, H8 and phenyl-H2, H6), 7.80 (d, *J* = 8 Hz, 2H, chlorophenyl-H3, H5 protons), 7.58 (t, *J* = 5.6, 1H, phenyl-H4), 7.27 (d, *J* = 7.6 Hz, 1H, quinoline-H7), 6.99 (d, *J* = 5.6 Hz, 2H, phenyl-H3, H5), 5.78 (t, *J* = 6.8, 1H, pyrazole-H5), 3.83 (dd, *J* = 4.4, *J* = 10, 1H, pyrazole- H4 axial proton), 3.76 (s, 3H, quinoline OCH_3_), 2.68 (dd, *J* = 7.2, *J* = 9.2, 1H, pyrazole-H4 equatorial proton); ^13 ^C NMR (DMSO-*d_6_*) δ: 159.4, 158.3, 153.6, 147.5, 134.2, 133.2, 132.3, 130.5, 129.5, 129.4, 129.3, 129.2, 128.2, 126.1, 123.8, 106.6, 57.6, 55.9, 36.9; MS (*m/z*) 452 (1.1%, M + 4), 450 (6.7%, M + 2), 448 (9.6%, M^+^), 255 (100%); Anal. Calc. for: (C_25_H_19_Cl_2_N_3_O): C, 66.97; H, 4.27; N, 9.37%; Found: C, 67.07; H, 4.34; N, 9.42%.

##### 4.1.2.13. 4-[5–(2-Chloro-6-methoxyquinolin-3-yl)-1-phenyl-4,5-dihydro-1H-pyrazol-3-yl]aniline (21)

Orange solid (55%) mp = 136–137 °C; IR (KBr) cm^−1^: 3315, 3297 (NH_2_), 3066 (CH aromatic), 2968 (CH aliphatic); ^1^H NMR (DMSO-*d_6_*) δ: 8.41 (s, 1H, quinoline-H4), 7.85 (d, *J* = 9.2 Hz, 1H, quinoline-H8), 7.54 (s, 1H, quinoline-H5), 7.44–7.37 (m, 3H, quinoline-H7 and aminophenyl-H2, H6 protons) , 7.19–7.12 (m, 3H, phenyl-H3, H5 and H4), 6.90 (d, *J* = 8, 2H, aminophenyl-H3, H5 protons), 6.57 (d, *J* = 8.4 Hz, 2H, phenyl-H3, H5), 5.47 (brs, 2H, NH_2_, D_2_O-exchangeable), 5.16 (t, *J* = 8 Hz, 1H, pyrazole-H5), 3.77 (s, 3H, quinoline OCH_3_), 3.65 (dd, *J* = 9.2 Hz, *J* = 16.8 Hz, 1H, pyrazole- H4 axial proton), 3.17 (dd, *J* = 10.4 Hz, *J* = 16.4 Hz, 1H, pyrazole-H4 equatorial proton); ^13 ^C NMR (DMSO-*d_6_*) δ: 160.1, 153.4, 150.3, 148.3, 142.7, 139.5, 137.8, 134.3, 132.3, 129.1, 128.4, 127.4, 126.7, 122.9, 121.8, 116.6, 105.9, 59.0, 55.2, 37.5; MS (*m/z*) 430 (9.3%, M^+^ +2), 428 (27.9%, M^+^), 236 (47%), 192 (100%); Anal. Calc. for: (C_25_H_21_ClN_4_O): C, 70.01; H, 4.94; N, 13.06%; Found: C, 70.08; H, 4.99; N, 13.12%.

##### 4.1.2.14. 2-Chloro-6-methoxy-3-[1-phenyl-3–(3,4,5-trimethoxyphenyl)-4,5-dihydro-1H-pyrazol-5-yl]quinoline (22)

Brown solid (58%) mp = 136–137 °C; IR (KBr) cm^−1^: 3024 (CH aromatic), 2941 (CH aliphatic); ^1^H NMR (DMSO-*d_6_*) δ: 8.46 (s, 1H, quinoline-H4), 8.00 (d, *J* = 9.2 Hz, 1H, quinoline-H8), 7.88 (d, *J* = 9.6 Hz, 1H, quinoline-H7), 7.73(d, *J* = 8 Hz, 2H, methoxyphenyl-H2, H6 protons), 7.58–7.46 (m, 6H, aromatic protons), 5.14 (t, *J* = 9.2 Hz, 1H, pyrazole-H5), 3.87 (s, 6H, 2OCH_3_), 3.76 (s, 6H, 2OCH_3_), 3.66 (dd, *J* = 5.2 Hz, *J* = 11 Hz, 1H, pyrazole- H4 axial proton), 2.86 (dd, *J* = 11.2 Hz, *J* = 15.2 Hz, 1H, 1H, pyrazole-H4 equatorial proton); ^13 ^C NMR (DMSO-*d_6_*) δ: 159.3, 157.9, 154.2, 153.4, 150.3, 149.3, 144.4, 142.4, 136.8, 133.3, 130.1, 129.1, 128.1, 127.4, 126.4, 123.6, 115.9, 106.2, 61.8, 56.9, 55.5, 37.8; MS (*m/z*) 505 (22%, M + 2), 503 (68%, M^+^), 311 (100%); Anal. Calc. for: (C_28_H_26_ClN_3_O_4_): C, 66.73; H, 5.20; N, 8.34%; Found: C, 66.79; H, 5.25; N, 8.38%.

##### 4.1.2.15. 2-Chloro-3-[3–(4-chlorophenyl)-1–(2,4-dinitrophenyl)-4,5-dihydro-1H-pyrazol-5-yl]-6-methoxyquinoline (23)

Yellow solid (63%) mp = 143–144 °C; IR (KBr) cm^−1^: 3047 (CH aromatic), 2973 (CH aliphatic); ^1^H NMR (DMSO-*d_6_*) δ: 9.54 (s, 1H, nitrophenyl-H3), 8.74 (d, *J* = 4.4 Hz, 1H, nitrophenyl-H5), 8.69 (d, *J* = 4.4 Hz, 1H, 1H, nitrophenyl-H6), 8.61 (s, 1H, quinoline-H4), 8.10 (d, *J* = 6.8 Hz, 2H, chlorophenyl-H2, H6 protons), 8.20 (s,1H, quinoline-H5), 7.95 (d, *J* = 5.2 Hz, 1H, quinoline-H8), 7.76 (d, *J* = 5.6 Hz, 1H, quinoline-H7), 7.56 (d, *J* = 5.2 Hz, 2H, chlorophenyl-H3, H5 protons), 4.61 (t, *J* = 10, 1H, pyrazole-H5), 3.74 (s, 3H, quinoline OCH_3_), 3.59 (dd, *J* = 9.2, *J* = 16.4, 1H, pyrazole- H4 axial proton), 2.78 (dd, *J* = 12, *J* = 17.2, 1H, pyrazole-H4 equatorial proton); ^13 ^C NMR (DMSO-*d_6_*) δ: 153.4, 150.3, 146.8, 144.7, 141.2, 137.8, 136.4, 134.3, 133.3, 130.8, 130.1, 129.4, 128.4, 128.2, 127.4, 126.4, 125.6, 123.9, 106.5, 56.9, 55.9, 37.1; MS (*m/z*) 542 (2.7%, M^+^ +4), 540 (16%, M + 2), 538 (24.6%, M^+^), 345 (100%); Anal. Calc. for: (C_25_H_17_Cl_2_N_5_O_5_): C, 55.78; H, 3.18; N, 13.01%; Found: C, 55.85; H, 3.24; N, 13.08%.

##### 4.1.2.16. 4-[5–(2-Chloro-6-methoxyquinolin-3-yl)-1–(2,4-dinitrophenyl)-4,5-dihydro-1H-pyrazol-3-yl]aniline (24)

Yellow solid (57%) mp = 139–140 °C; IR (KBr) cm^−1^: 3300, 3292 (NH_2_), 3021 (CH aromatic), 2923 (CH aliphatic); ^1^H NMR (DMSO-*d_6_*) δ: 8.85 (s, 1H, nitrophenyl-H3), 8.83 (d, *J* = 2.8 Hz, 1H, nitrophenyl-H5), 8.41 (s, 1H, quinoline-H4), 8.29 (d, *J* = 2.8 Hz, 1H, nitrophenyl-H6), 8.0 (d, *J* = 9.2 Hz, 1H, quinoline-H8), 7.77 (d, *J* = 9.2, 1H, quinoline-H7), 7.47 (s, 1H, quinoline-H5), 7.40 (d, *J* = 8.8 Hz, 2H, aminophenyl-H2, H6), 6.84 (d, *J* = 8.8 Hz, 2H, aminophenyl-H3, H5), 5.36 (brs, 2H, NH_2_, D_2_O-exchangeable), 4.98 (t, *J* = 6 Hz, 1H, pyrazole-H5), 3.88 (s, 3H, quinoline OCH_3_), 3.70 (dd, *J* = 10 Hz, *J* = 14.4 Hz, 1H, pyrazole- H4 axial proton), 2.82 (dd, *J* = 9.6 Hz, *J* = 17.2 Hz, 1H, pyrazole-H4 equatorial proton); ^13 ^C NMR (DMSO-*d_6_*) δ: 162.4, 157.6, 156.9, 153.5, 150.6, 149.3, 145.8, 142.4, 139.2, 137.5, 133.3, 131.9, 129.1, 128.4, 127.7, 127.1, 123.9, 121.5, 116.3, 106.9, 57.2, 55.5, 37.5; MS (*m/z*) 520 (9.3%, M + 2), 518 (27.9%, M^+^), 326 (38%), 192 (100%); Anal. Calc. for: (C_25_H_19_ClN_6_O_5_): C, 57.87; H, 3.69; N, 16.20%; Found: C, 57.93; H, 3.74; N, 16.26%.

##### 4.1.2.17. 2-Chloro-3-[1–(2,4-dinitrophenyl)-3–(3,4,5-trimethoxyphenyl)-4,5-dihydro-1H-pyrazol-5-yl]-6-methoxyquinoline (25)

Orange solid (61%) mp = 135–136 °C; IR (KBr) cm^−1^: 3039 (CH aromatic), 2922 (CH aliphatic); ^1^H NMR (DMSO-*d_6_*) δ: 9.03 (s, 1H, nitrophenyl-H3), 8.94 (d, *J* = 5.7 Hz, 1H, nitrophenyl-H5), 8.43 (s, 1H, quinoline-H4), 7.97 (d, *J* = 9.3 Hz, 1H, quinoline-H8), 7.52 (s, 2H, methoxyphenyl-H2, H6), 7.50 (d, *J* = 9.3 Hz, 1H, quinoline-H7), 7.42 (s, 1H, quinoline-H5), 7.34(d, *J* = 5.7 Hz, 1H, 1H, nitrophenyl-H6), 5.30 (t, *J* = 5.4 Hz, 1H, pyrazole-H5), 3.94 (s, 6H, 2OCH_3_), 3.91 (s, 6H, 2OCH_3_), 3.71 (dd, *J* = 7.2 Hz, *J* = 14.4 Hz, 1H, pyrazole- H4 axial proton), 2.52 (dd, *J* = 6.3 Hz, *J* = 12.3 Hz, 1H, pyrazole-H4 equatorial proton); ^13 ^C NMR (DMSO-*d_6_*) δ: 157.9, 156.2, 155.5, 153.8, 153.1, 149.3, 144.7, 142.7, 136.8, 134.0, 133.3, 129.4, 129.1, 127.7, 127.4, 126.0, 123.6, 119.4, 115.6, 106.5, 56.9, 55.5, 55.2, 51.3, 37.1; MS (*m/z*) 595 (9%, M^+^ +2), 593 (28%, M^+^), 401 (100%); Anal. Calc. for: (C_28_H_24_ClN_5_O_8_): C, 56.62; H, 4.07; N, 11.79%; Found: C, 56.65; H, 4.10; N, 11.84%.

#### 4.1.3. General procedure for synthesis of compounds (26–31)

A mixture of chalcone **5–7** (10 mmole) and thiourea or guanidine hydrochloride (10 mmole) was stirred in absolute ethanol (20 mL), and then sodium hydroxide (0.8 g, 20 mmole) was added. The reaction mixture was heated under reflux for 8 h. After completion of reaction, as detected by TLC, the solvent was concentrated under reduced pressure, and then poured into ice water (50 mL). The obtained solid was filtered off, washed and recrystallized from ethanol to afford the desired compounds **26–31**.

##### 4.1.3.1. 6–(2-Chloro-6-methoxyquinolin-3-yl)-4–(4-chlorophenyl)pyrimidine-2(1H)-thione (26)

Yellow solid (54%) mp = 155–156 °C; IR (KBr) cm^−1^: 3308 (NH), 3056 (CH aromatic), 2969 (CH aliphatic); ^1^H NMR (DMSO-*d_6_*) δ: 14.16 (brs, 1H, NH, D_2_O-exchangeable), 8.61 (s, 1H, quinoline-H4), 8.34 (d, *J* = 8.8 Hz, 1H, quinoline-H8), 8.14 (d, *J* = 8.4 Hz, 2H, phenyl-H2, H6 protons), 7.86 (d, *J* = 8.8 Hz, 1H, quinoline-H7), 7.55 (d, *J* = 8.4 Hz, 2H, phenyl-H3, H5 protons), 7.42 (s, 1H, pyrimidine-H5), 7.33 (s, 1H, quinoline-H5), 3.81 (s, 3H, quinoline OCH_3_); ^13 ^C NMR (DMSO-*d_6_*) δ: 166.6, 160.1, 159.0, 157.9, 153.8, 147.5, 144.7, 142.3, 134.3, 133.3, 130.5, 129.1, 128.4, 128.1, 126.4, 123.9, 106.2, 55.9; MS (*m/z*) 418 (2.4%, M + 4), 416 (15.4%, M + 2), 414 (22.5%, M^+^), 220 (33%), 192 (100%); Anal. Calc. for: (C_20_H_13_Cl_2_N_3_OS): C, 57.98; H, 3.16; N, 10.14%; Found: C, 58.04; H, 3.22; N, 10.19%.

##### 4.1.3.2. 4–(4-Aminophenyl)-6–(2-chloro-6-methoxyquinolin-3-yl)pyrimidine-2(1H)-thione (27)

Yellow solid (63%) mp = 146–147 °C; IR (KBr) cm^−1^: 3311, 3299 (NH_2_), 3039 (CH aromatic), 2974 (CH aliphatic); ^1^H NMR (DMSO-*d_6_*) δ: 12.74 (brs, 1H, NH, D_2_O-exchangeable), 8.09 (s, 1H, quinoline-H4), 7.86 (d, *J* = 8.8 Hz, 1H, quinoline-H8), 7.45 (d, *J* = 8.8 Hz, 1H, quinoline-H7), 7.41 (s, 1H, pyrimidine-H5), 7.15 (d, *J* = 8.4 Hz, 2H, phenyl-H2, H6 protons), 6.49 (d, *J* = 8.4 Hz, 2H, phenyl-H2, H6 protons), 6.41 (s, 1H, quinoline-H5), 5.32 (brs, 2H, NH_2_, D_2_O-exchangeable), 3.88 (s, 3H, quinoline OCH_3_); ^13 ^C NMR (DMSO-*d_6_*) δ: 165.9, 159.7, 159.0, 157.6, 153.4, 148.3, 142.7, 139.5, 131.6, 129.5, 128.1, 127.4, 126.7, 123.2, 121.2, 116.3, 106.2, 55.5; MS (*m/z*) 396 (5.4%, M^+^ +2), 394 (16.6%, M^+^), 202 (39%), 302 (100%); Anal. Calc. for: (C_20_H_15_ClN_4_OS): C, 60.83; H, 3.83; N, 14.19%; Found: C, 60.87; H, 3.88; N, 14.22%.

##### 4.1.3.3. 6–(2-Chloro-6-methoxyquinolin-3-yl)-4–(3,4,5-trimethoxyphenyl)pyrimidine-2(1H)-thione (28)

Yellow solid (54%) mp = 141–142 °C; IR (KBr) cm^−1^: 3275 (NH), 3028 (CH aromatic), 2932 (CH aliphatic); ^1^H NMR (DMSO-*d_6_*) δ: 10.83 (brs, 1H, NH, D_2_O-exchangeable), 8.75 (s, 1H, quinoline-H4), 7.83 (d, *J* = 9.3 Hz, 1H, quinoline-H8), 7.52 (d, *J* = 9.3 Hz, 1H, quinoline-H7), 7.37 (s, 1H, pyrimidine-H5), 7.18 (s, 2H, phenyl-H2, H6 protons), 7.07 (s, 1H, quinoline-H5), 3.94 (s, 6H, 2OCH_3_), 3.89 (s, 6H, 2OCH_3_); ^13 ^C NMR (DMSO-*d_6_*) δ: 166.6, 163.8, 159.1, 153.8, 152.8, 148.9, 144.7, 142.7, 136.8, 134.3, 133.6, 128.4, 127.7, 126.4, 123.6, 115.6, 106.5, 56.5, 54.8, 54.4; MS (*m/z*) 471 (3%, M^+^ +2), 469 (10%, M^+^), 70 (100%); Anal. Calc. for: (C_23_H_20_ClN_3_O_4_S): C, 58.78; H, 4.29; N, 8.94%; Found: C, 58.79; H, 4.33; N, 8.99%.

##### 4.1.3.4. 4–(2-Chloro-6-methoxyquinolin-3-yl)-6–(4-chlorophenyl)pyrimidin-2-amine (29)

Yellow solid (72%) mp = 195–196 °C; IR (KBr) cm^−1^: 3321, 3284 (NH_2_), 3049 (CH aromatic), 2972 (CH aliphatic); ^1^H NMR (DMSO-*d_6_*) δ: 8.49 (s, 1H, quinoline-H4), 8.37 (d, *J* = 8.8 Hz, 1H, quinoline-H8), 7.79 (d, *J* = 8.4 Hz, 2H, phenyl-H2, H6 protons), 7.50 (s,1H, pyrimidine-H5), 7.11 (s, 1H, quinoline-H5), 7.05 (d, *J* = 8.4 Hz, 2H, phenyl-H3, H5 protons), 6.78 (d, *J* = 8.8 Hz, 1H, quinoline-H7), 5.41 (brs, 2H, NH_2_, D_2_O-exchangeable), 3.72 (s, 3H, quinoline OCH_3_); ^13 ^C NMR (DMSO-*d_6_*) δ: 162.8, 160.1, 159.4, 157.9, 153.8, 146.5, 144.4, 142.7, 134.0, 132.6, 130.5, 129.1, 128.1, 127.4, 126.0, 123.6, 106.5, 55.5; MS (*m/z*) 376 (1.4%, M + 4), 374 (7.4%, M^+^ +2), 372 (11%, M^+^), 204 (33%), 192 (100%); Anal. Calc. for: (C_20_H_14_Cl_2_N_4_O): C, 60.47; H, 3.55; N, 14.10%; Found: C, 60.54; H, 3.61; N, 14.17%.

##### 4.1.3.5. 4–(4-Aminophenyl)-6–(2-chloro-6-methoxyquinolin-3-yl)pyrimidin-2-amine (30)

Yellow solid (58%) mp = 175–176 °C; IR (KBr) cm^−1^: 3327, 3289 (NH_2_), 3028 (CH aromatic), 2918 (CH aliphatic); ^1^H NMR (DMSO-*d_6_*) δ: 8.51 (s, 1H, quinoline-H4), 8.13 (s, 1H, pyrimidine-H5), 7.84 (d, *J* = 8.4 Hz, 1H, quinoline-H7), 7.62 (d, *J* = 8.8 Hz, 2H, phenyl-H2, H6 protons), 7.43 (d, *J* = 8.4 Hz, 1H, quinoline-H8), 7.25 (s, 1H, quinoline-H5), 6.82 (d, *J* = 8.8 Hz, 2H, phenyl-H3, H5 protons), 5.50 (brs, 2H, NH_2_, D_2_O-exchangeable), 5.30 (brs, 2H, NH_2_, D_2_O-exchangeable), 3.71 (s, 3H, quinoline OCH_3_); ^13 ^C NMR (DMSO-*d_6_*) δ: 164.2, 162.4, 159.4, 157.6, 153.8, 148.9, 142.3, 139.9, 132.3, 129.4, 128.4, 127.7, 126.7, 121.5, 119.0, 116.6, 105.9, 55.2; MS (*m/z*) 379 (11.4%, M^+^ +2), 377 (35.6%, M^+^), 185 (66%), 302 (100%); Anal. Calc. for: (C_20_H_16_ClN_5_O): C, 63.58; H, 4.27; N, 18.54%; Found: C, 63.66; H, 4.33; N, 18.59%.

##### 4.1.3.6. 4–(2-Chloro-6-methoxyquinolin-3-yl)-6–(3,4,5-trimethoxyphenyl)pyrimidin-2-amine (31)

brown solid (69%) mp = 162–163 °C; IR (KBr) cm^−1^: 3303, 3291 (NH_2_), 3024 (CH aromatic), 2949 (CH aliphatic); ^1^H NMR (DMSO-*d_6_*) δ: 8.53 (s, 1H, quinoline-H4), 7.95 (d, *J* = 9 Hz, 1H, quinoline-H8), 7.54 (s, 1H, pyrimidine-H5), 7.51 (d, *J* = 9 Hz, 1H, quinoline-H7), 7.42 (s, 1H, quinoline-H5), 7.25 (s, 2H, phenyl-H2, H6 protons), 6.8 (brs, 2H, NH_2_, D_2_O-exchangeable), 3.91 (s, 6H, 2OCH_3_), 3.83 (s, 6H, 2OCH_3_); ^13 ^C NMR (DMSO-*d_6_*) δ: 168.3, 162.1, 159.4, 153.8, 153.5, 149.6, 144.7, 142.4, 136.7, 134.0, 133.0, 128.1, 127.4, 126.4, 123.6, 115.3, 106.2, 56.9, 55.2, 54.8; MS (*m/z*) 454 (0.6%, M + 2), 452 (2%, M^+^), 192 (100%); Anal. Calc. for: (C_23_H_21_ClN_4_O_4_): C, 61.00; H, 4.67; N, 12.37%; Found: C, 61.07; H, 4.70; N, 12.44%.

#### 4.1.4. General procedure for synthesis of compounds (32–34)

A mixture of chalcone **5–7** (10 mmole) and urea (0.6 g, 10 mmole) was stirred in ethanol (20 mL), and then hydrochloric acid (2 mL) was added. The mixture was heated at reflux for 12 h. After completion the reaction, the solvent was concentrated under reduced pressure and poured into ice water (50 mL). The obtained precipitate was filtered off, washed and recrystallized from ethanol to yield the titled compounds **32–34**.

##### 4.1.4.1. 6–(2-Chloro-6-methoxyquinolin-3-yl)-4–(4-chlorophenyl)pyrimidin-2(1H)-one (32)

Yellow solid (76%) mp = 180–181 °C; IR (KBr) cm^−1^: 3332 (NH), 3078 (CH aromatic), 2932 (CH aliphatic); ^1^H NMR (DMSO-*d_6_*) δ: 11.96 (brs, 1H, NH, D_2_O-exchangeable), 8.51 (s, 1H, quinoline-H4), 8.33 (d, *J* = 8.2 Hz, 1H, quinoline-H8), 8.06 (d, *J* = 8.8 Hz, 2H, phenyl-H2, H6 protons), 7.77 (d, *J* = 8.2 Hz, 1H, quinoline-H7), 7.28 (s,1H, pyrimidine-H5), 7.20 (s, 1H, quinoline-H5), 7.08 (d, *J* = 8.8 Hz, 2H, phenyl-H3, H5 protons), 3.74 (s, 3H, quinoline OCH_3_); ^13 ^C NMR (DMSO-*d_6_*) δ: 166.3, 160.1, 159.0, 157.9, 153.8, 147.5, 144.7, 142.7, 134.3, 133.6, 130.5, 129.8, 128.1, 127.1, 126.0, 123.6, 105.5, 55.2; MS (*m/z*) 402 (1.4%, M^+^ +4), 400 (9%, M + 2), 398 (13%, M^+^), 204 (33%), 192 (100%); Anal. Calc. for: (C_20_H_13_Cl_2_N_3_O_2_): C, 60.32; H, 3.29; N, 10.55%; Found: C, 60.40; H, 3.35; N, 10.61%.

##### 4.1.4.2. 4–(4-Aminophenyl)-6–(2-chloro-6-methoxyquinolin-3-yl)pyrimidin-2(1H)-one (33)

Yellow solid (76%) mp = 171–172 °C; IR (KBr) cm^−1^: 3322, 3285 (NH_2_), 3023 (CH aromatic), 2917 (CH aliphatic); ^1^H NMR (DMSO-*d_6_*) δ: 10.41 (brs, 1H, NH, D_2_O-exchangeable), 8.12 (s, 1H, quinoline-H4), 7.84 (d, *J* = 8.8 Hz, 2H, phenyl-H2, H6 protons), 7.59 (d, *J* = 8.4 Hz, 1H, quinoline-H8), 7.30 (d, *J* = 8.4 Hz, 1H, quinoline-H7), 7.20 (s, 1H, pyrimidine-H5), 6.62 (d, *J* = 8.8 Hz, 2H, phenyl-H3, H5 protons), 6.15 (s, 1H, quinoline-H5), 5.32 (brs, 2H, NH_2_, D_2_O-exchangeable), 3.79 (s, 3H, quinoline OCH_3_); ^13 ^C NMR (DMSO-*d_6_*) δ: 169.8, 160.1, 159.0, 157.6, 153.8, 148.9, 142.4, 139.5, 131.9, 129.1, 128.4, 127.7, 127.1, 124.2, 121.8, 116.3, 105.9, 55.2; MS (*m/z*) 380 (9.1%, M + 2), 378 (28.6%, M^+^), 92 (25%), 286 (100%); Anal. Calc. for: (C_20_H_15_ClN_4_O_2_): C, 63.41; H, 3.99; N, 14.79%; Found: C, 63.44; H, 4.02; N, 14.83%.

##### 4.1.4.3. 6–(2-Chloro-6-methoxyquinolin-3-yl)-4–(3,4,5-trimethoxyphenyl)pyrimidin-2(1H)-one (34)

Yellow solid (74%) mp = 176–177 °C; IR (KBr) cm^−1^: 3319 (NH), 3055 (CH aromatic), 2912 (CH aliphatic); ^1^H NMR (DMSO-*d_6_*) δ: 11.96 (brs, 1H, NH, D_2_O-exchangeable), 8.52 (s, 1H, quinoline-H4), 8.35 (d, *J* = 9 Hz, 1H, quinoline-H8), 7.79 (d, *J* = 9 Hz, 1H, quinoline-H7), 7.32 (s, 1H, pyrimidine-H5), 7.24 (s, 2H, phenyl-H2, H6 protons), 7.13 (s, 1H, quinoline-H5), 3.88 (s, 6H, 2OCH_3_), 3.82 (s, 6H, 2OCH_3_); ^13 ^C NMR (DMSO-*d_6_*) δ: 162.4, 161.1, 159.4, 153.5, 153.1, 148.9, 144.7, 142.3, 136.8, 134.3, 137.3, 128.4, 128.1, 126.7, 123.9, 115.9, 106.5, 56.9, 55.2, 54.8; MS (*m/z*) 455 (1.6%, M^+^ +2), 453 (6%, M^+^), 167 (100%); Anal. Calc. for: (C_23_H_20_ClN_3_O_5_): C, 60.86; H, 4.44; N, 9.26%; Found: C, 60.92; H, 4.50; N, 9.29%.

### 4.2. Biological evaluation

#### 4.2.1. *In vitro* cytotoxic activity

Evaluation of cytotoxic activity of the synthesised compounds was carried out using MTT assay protocol^46–48^ against a group of cancer cell lines namely; colorectal carcinoma (HCT-116), Hepatocellular carcinoma (HepG-2) and breast cancer (MCF-7) and colchicine was used as a standard drug. The complete procedure was depected in Supplementary data.

#### 4.2.2. In vitro tubulin polymerisation assay

The effect of the synthesised compounds on tubulin polymerisation was assessed turbidimetrically using a fluorescent plate reader method^49^. The complete procedure was depected in Supplementary data.

#### 4.2.3. Cell cycle analysis

The effect of the most promising compound 25 on cell cycle was evaluated using flowcytometer^50^^,^^53^^,^^54^ as illustrated in Supplementary data.

#### 4.2.4. Annexin V-FITC apoptosis assay

The effect of compound **25** on apoptosis induction was analysed using Annexin V-FITC/PI apoptosis detection kit using flowcytometer^51^^,^^55^^,^^56^ as illustrated in Supplementary data.

### 4.3. Docking studies

The Crystal structure of the target receptor (tubulin) [PDB ID: 1SA0, resolution 3.00 Å] was obtained from Protein Data Bank (http://www.pdb.org). The docking process was carried out using MOE2014 software. At first, the crystal structure of the target was prepared by removing water molecules and retaining the two essential chains and the co-crystallised ligand, *N*-deacetyl-*N*-(2-mercaptoacetyl)-colchicine (DAMA-colchicine). Then, the protein structure was protonated, and the hydrogen atoms were hided. Next, the energy was minimised, and the binding pocket of the protein was defined.

The 2D structures of the synthesised compounds and reference ligand (DAMA-colchicine) were sketched using ChemBioDraw Ultra 14.0 and saved as MDL-SD format^57^. Then, the saved files were opened using MOE and 3D structures were protonated. Next, energy minimisation was applied. Before docking process, validation of the docking protocol was carried out by running the simulation only using the co-crystallised ligand (DAMA-colchicine) which showed low RMSD value. The molecular docking of the synthesised was performed using a default protocol against the target receptor. In each case, 30 docked structures were generated using genetic algorithm searches, London dG was used for scoring and forcefield (MMFF94) for refinement. The London dG scoring function estimates the free energy of binding of the ligand from a given pose. The output from of MOE was further analysed and visualised using Discovery Studio 4.0 software.^58–63^

## Supplementary Material

Supplemental Material
