# Supplementary material for: Discovery of new quinolines as potent colchicine binding site inhibitors: design, synthesis, docking studies, and anti-proliferative evaluation
Source: J Enzyme Inhib Med Chem. 2021 Feb 15;36(1):640–58. doi: 10.1080/14756366.2021.1883598 (PMC7889231; doi:10.1080/14756366.2021.1883598)
Supplement: Supplemental Material [file IENZ_A_1883598_SM5111.pdf]

Discovery of new quinolines as potent colchicine binding site inhibitors: Design, synthesis, docking studies, and anti-proliferative evaluation

Mohamed Hagras*[a], Moshira A. El Deeb[b], Heba S. A. Elzahabi[c], Eslam B. Elkaeed[d,a], Ahmed B.M. Mehany[e], Ibrahim H. Eissa*[f]

[a] Pharmaceutical Organic Chemistry, Faculty of Pharmacy (Boys), Al-Azhar University, Cairo, Egypt

[b] Pharmaceutical Organic Chemistry, Faculty of Pharmacy (Girls), Al-Azhar University, Cairo, Egypt

[c] Pharmaceutical Medicinal Chemistry & Drug Design Department, Faculty of Pharmacy (Girls), Al-Azhar University, Cairo, Egypt

[d] Department of Pharmaceutical Sciences, College of Pharmacy, AlMaarefa University, Ad Diriyah 13713, Saudi Arabia

[e] Zoology Department, Faculty of Science, Al-Azhar University, Cairo, Egypt.

[f] Pharmaceutical Medicinal Chemistry & Drug Design Department, Faculty of Pharmacy (Boys), Al-Azhar University, Cairo, Egypt

*** Corresponding authors:**

Ibrahim H. Eissa

Pharmaceutical Medicinal Chemistry & Drug Design Department, Faculty of Pharmacy (Boys), Al-Azhar University, Cairo, 11884, Egypt

Email: Ibrahimeissa@azhar.edu.eg

Mohamed Hagras

Pharmaceutical Organic Chemistry, Faculty of Pharmacy (Boys), Al-Azhar University, Cairo, 11884, Egypt

E-mail: m.hagrs@azhar.edu.eg

3D structure of compound **21** docked into the colchicine binding site

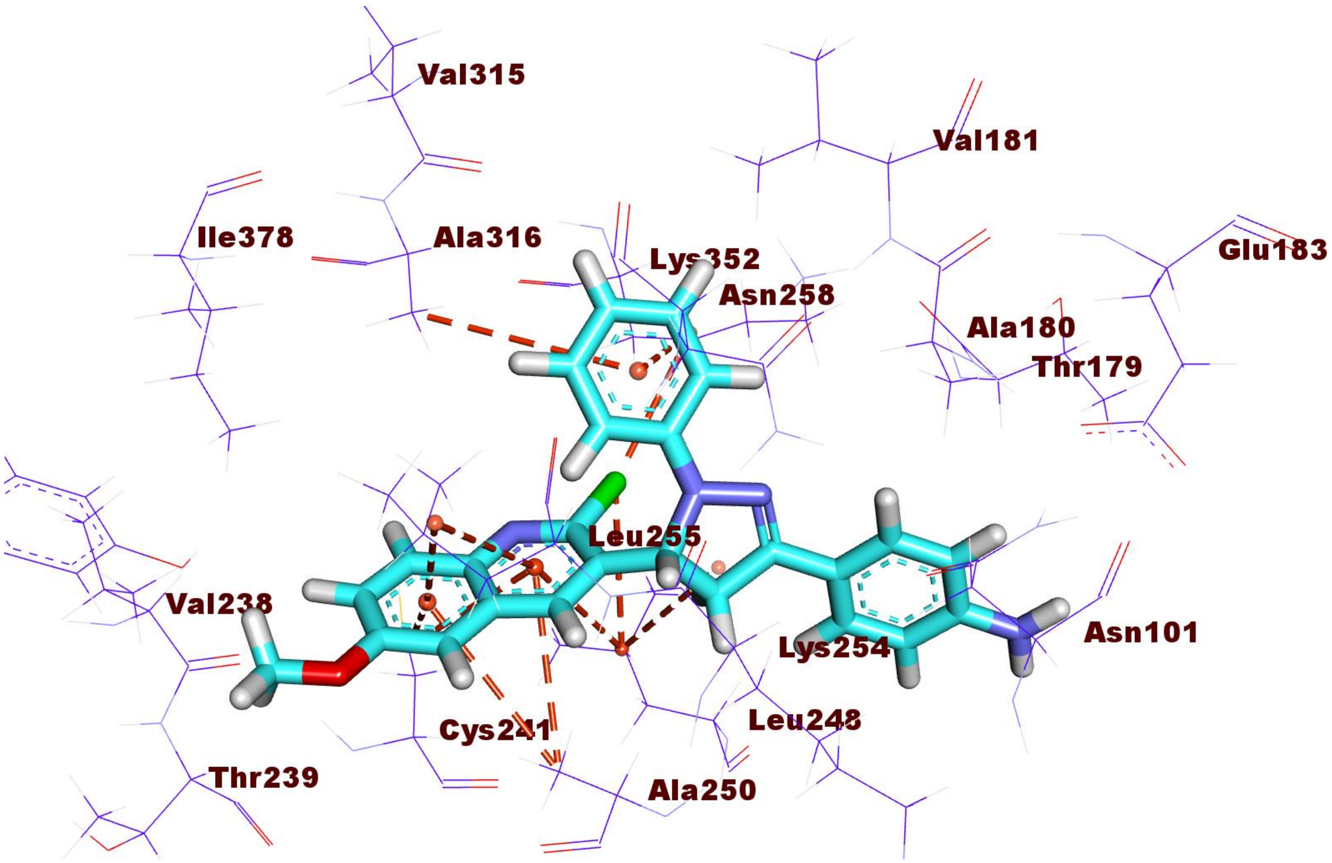

2D structure of compound **21** docked into the colchicine binding site

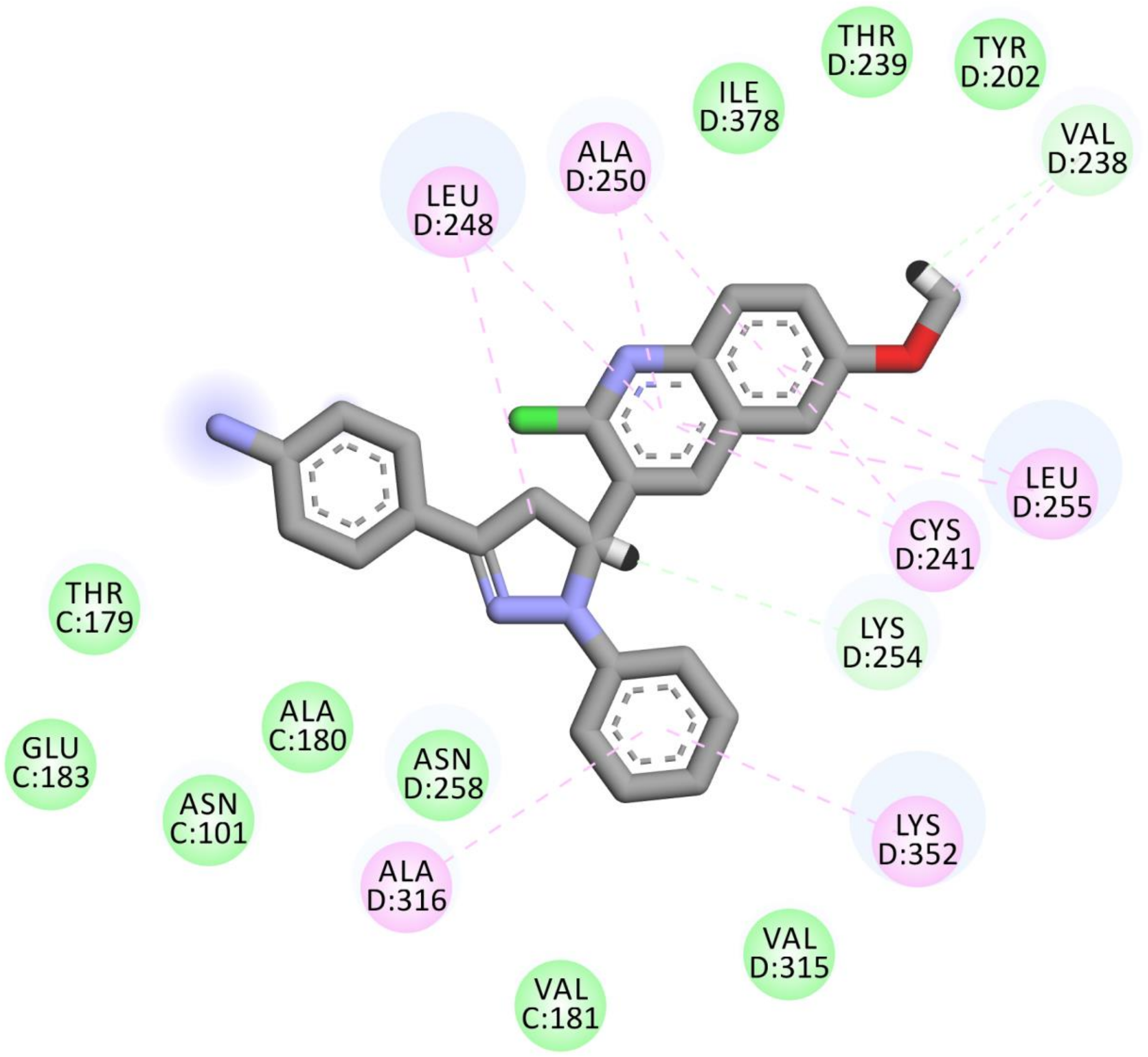

Mapping surface showing compound **21** occupying the active pocket of colchicine binding site

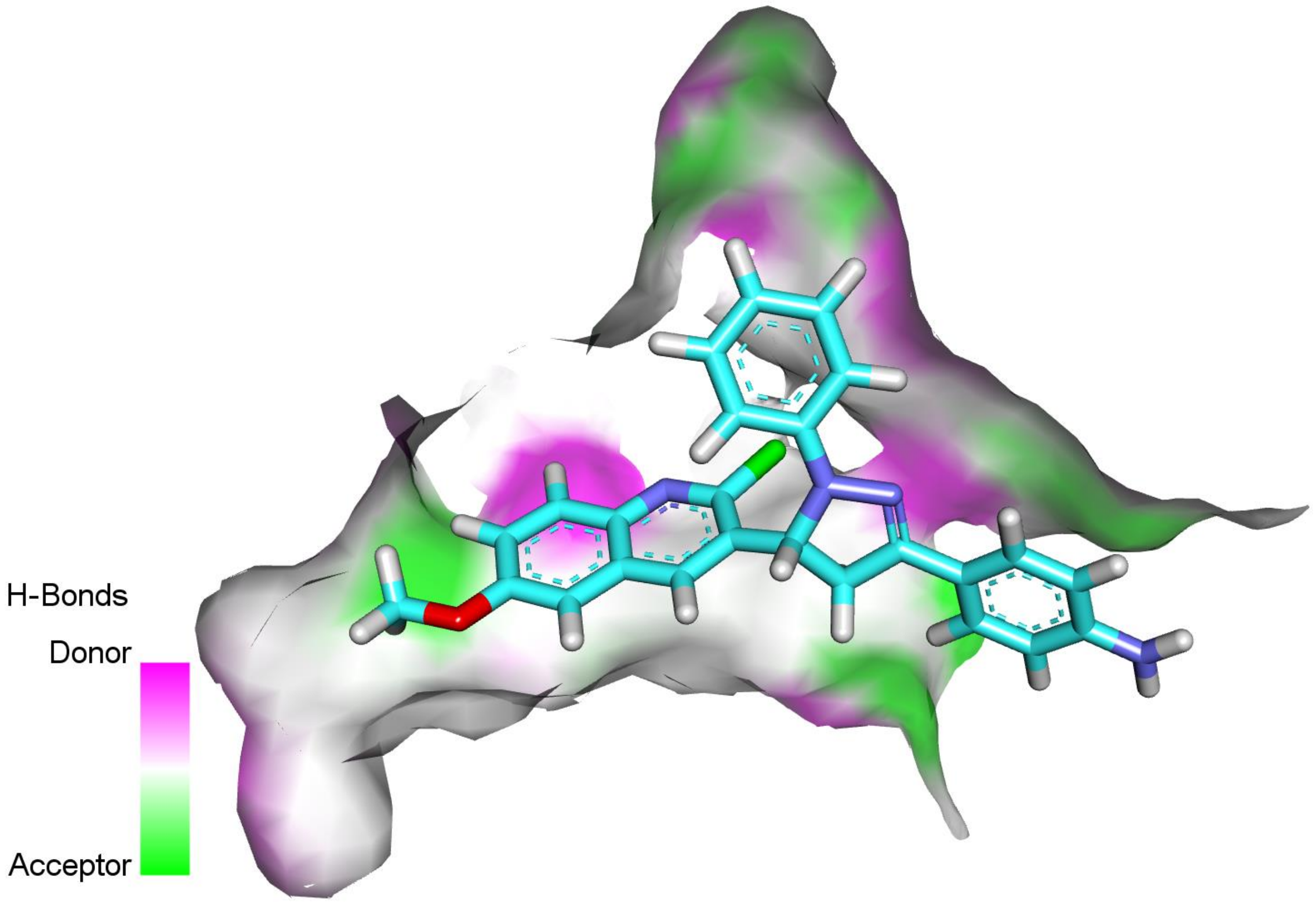

3D structure of compound **32** docked into the colchicine binding site

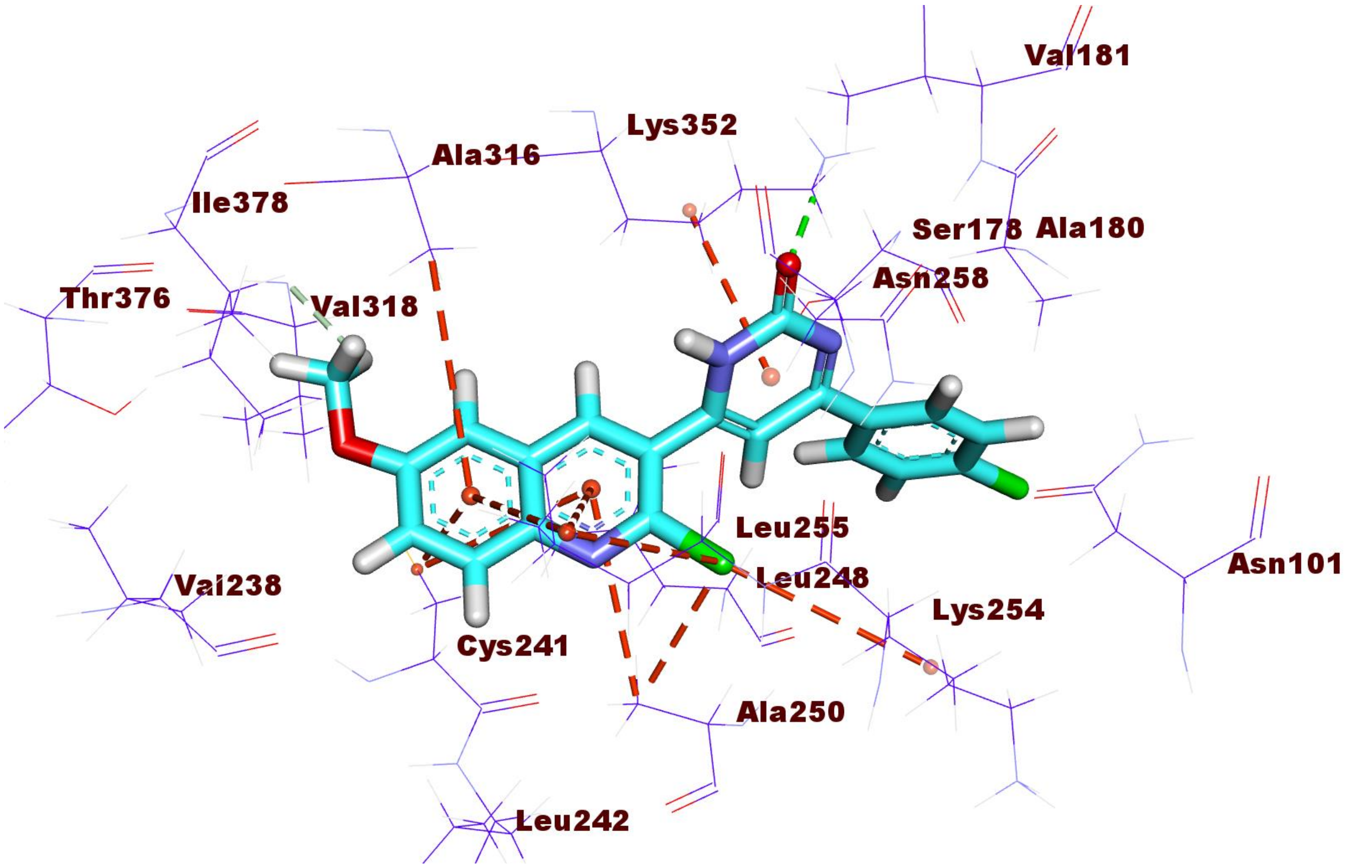

2D structure of compound **32** docked into the colchicine binding site

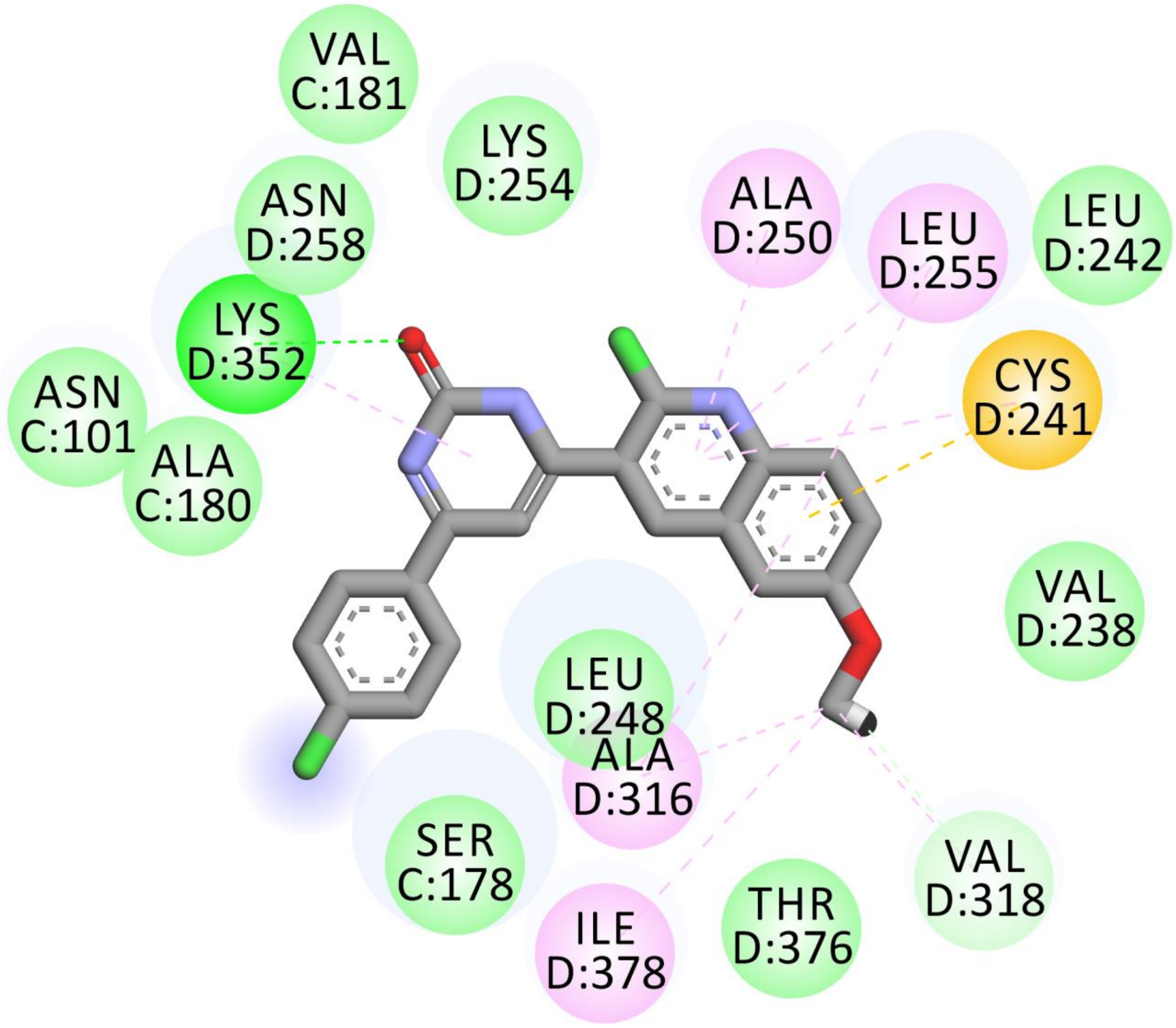

Mapping surface showing compound **32** occupying the active pocket of colchicine binding site

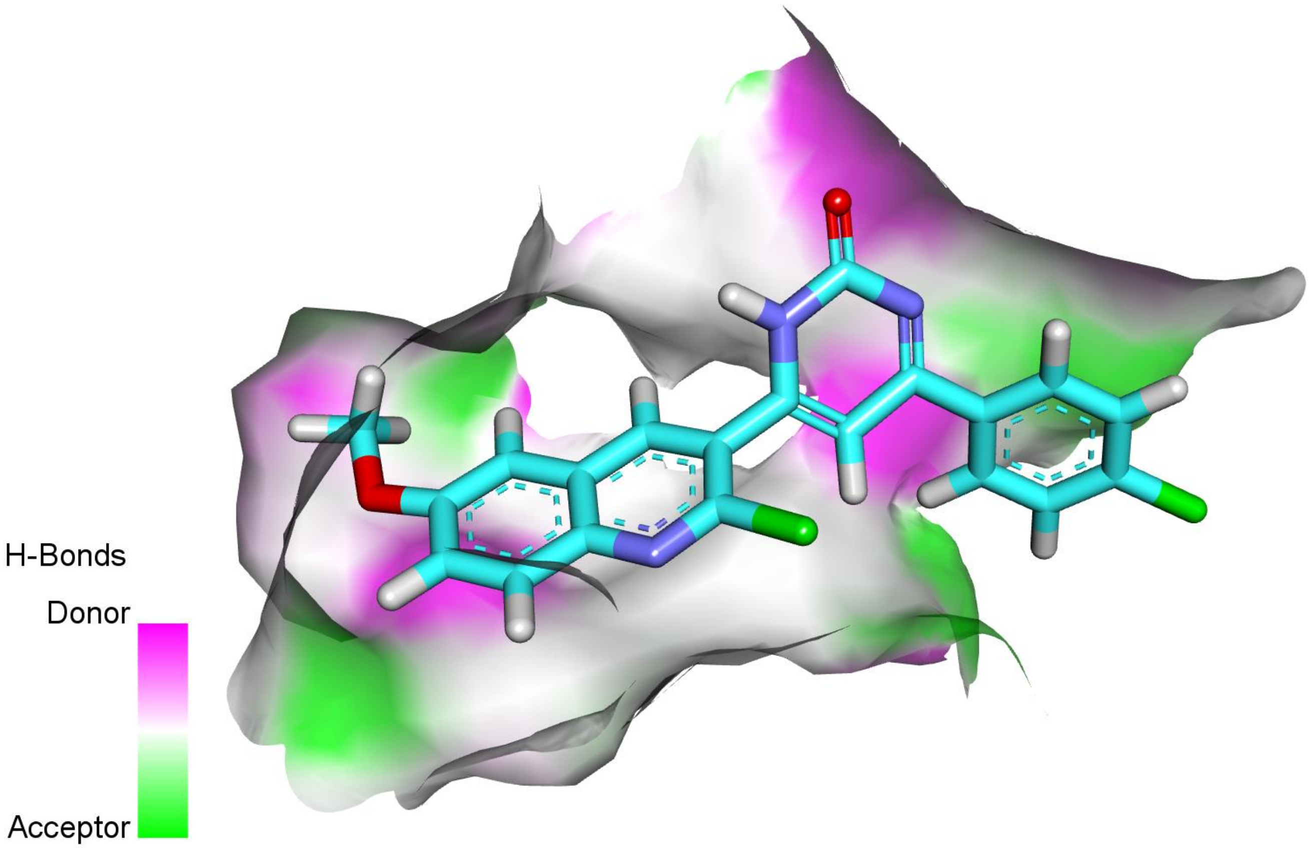

Mapping surface showing compound **19** occupying the active pocket of colchicine binding site

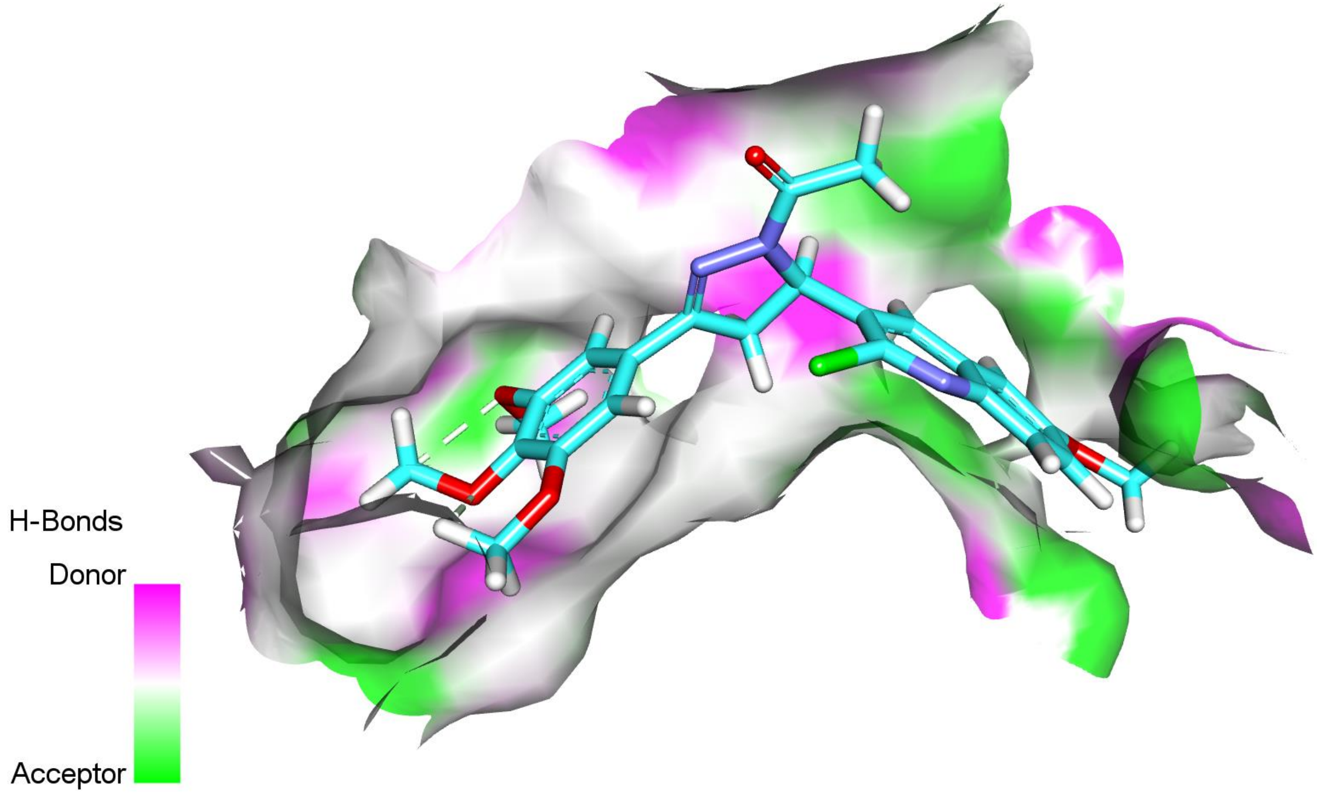

Mapping surface showing compound **25** occupying the active pocket of colchicine binding site

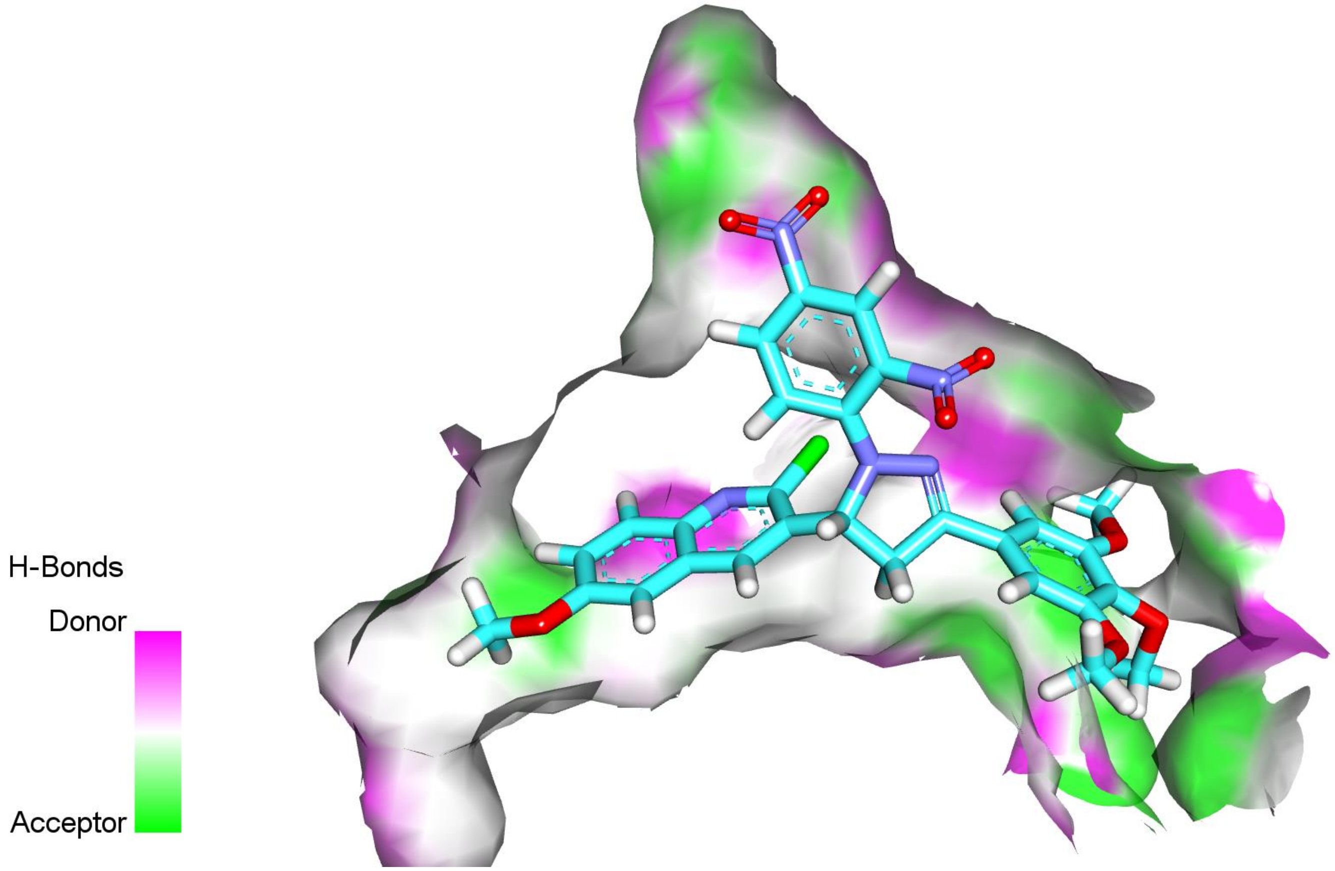

Mapping surface showing compound **32** occupying the active pocket of colchicine binding site

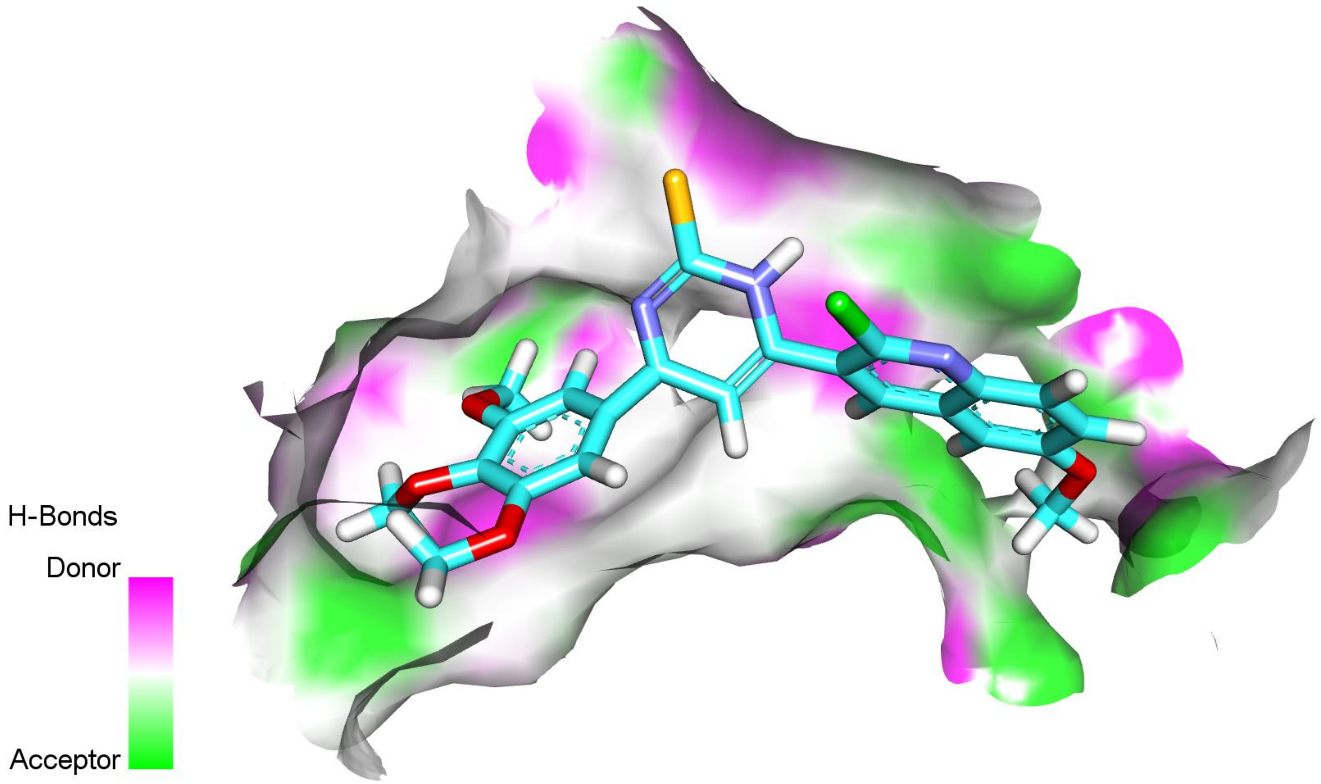

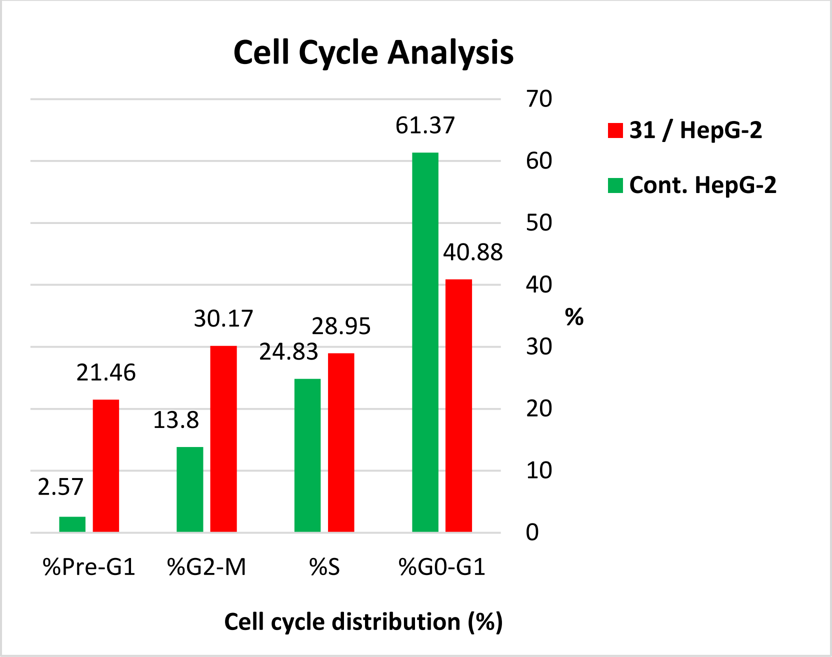

HepG-2 cells distribution upon treatment with compound **25**

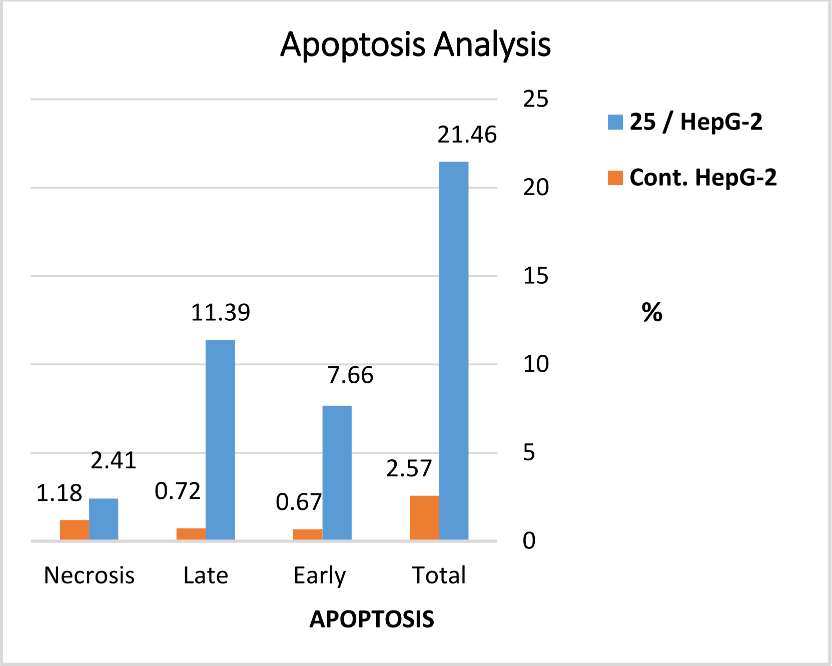

Induced apoptosis on HepG-2 cells by compound **25**

The proposed mechanism of reaction to form compound **5**

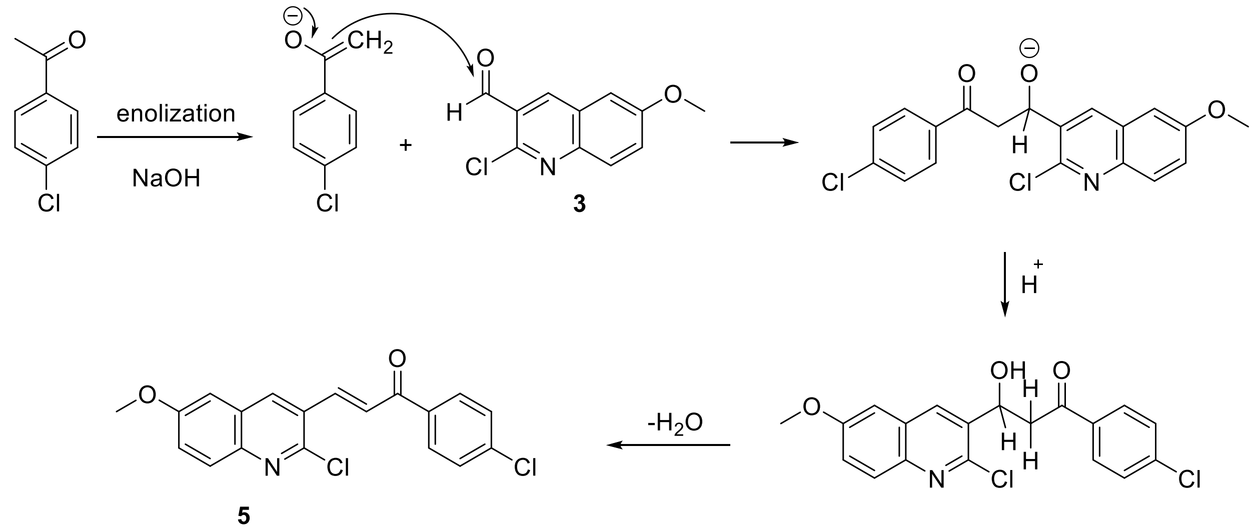

The proposed mechanism of reaction to form compound **8**

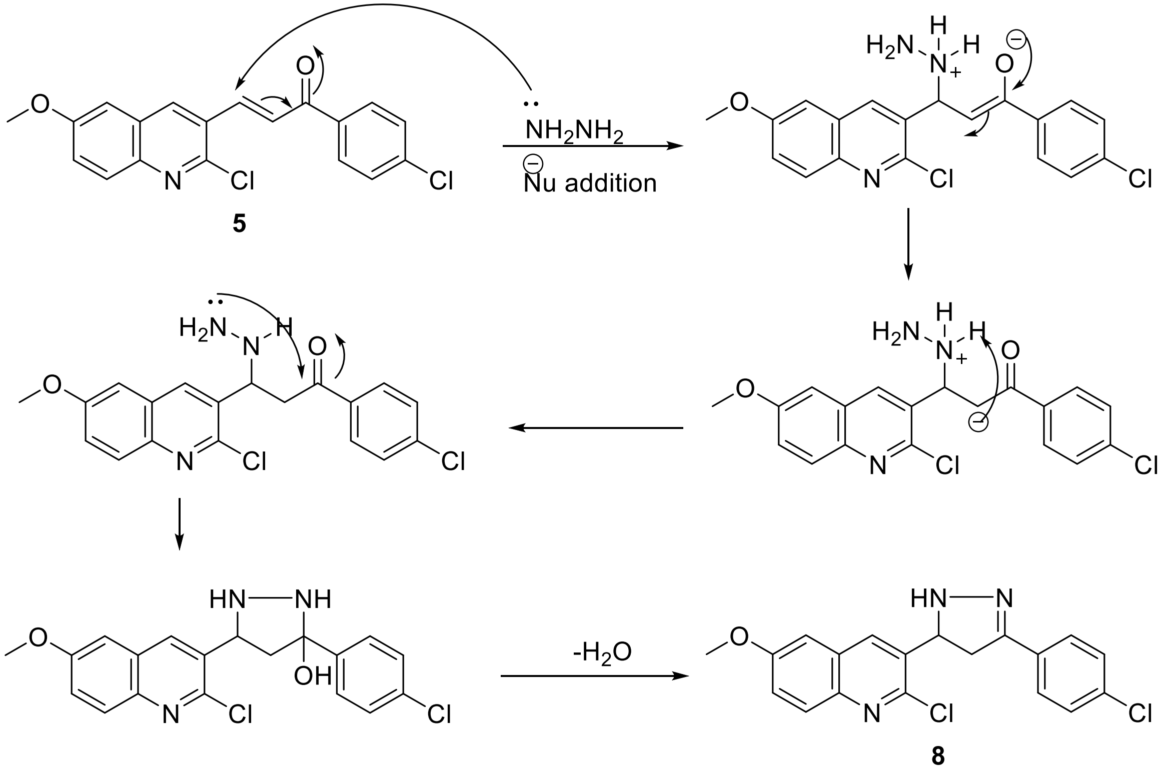

The proposed mechanism of reaction to form compound **11**

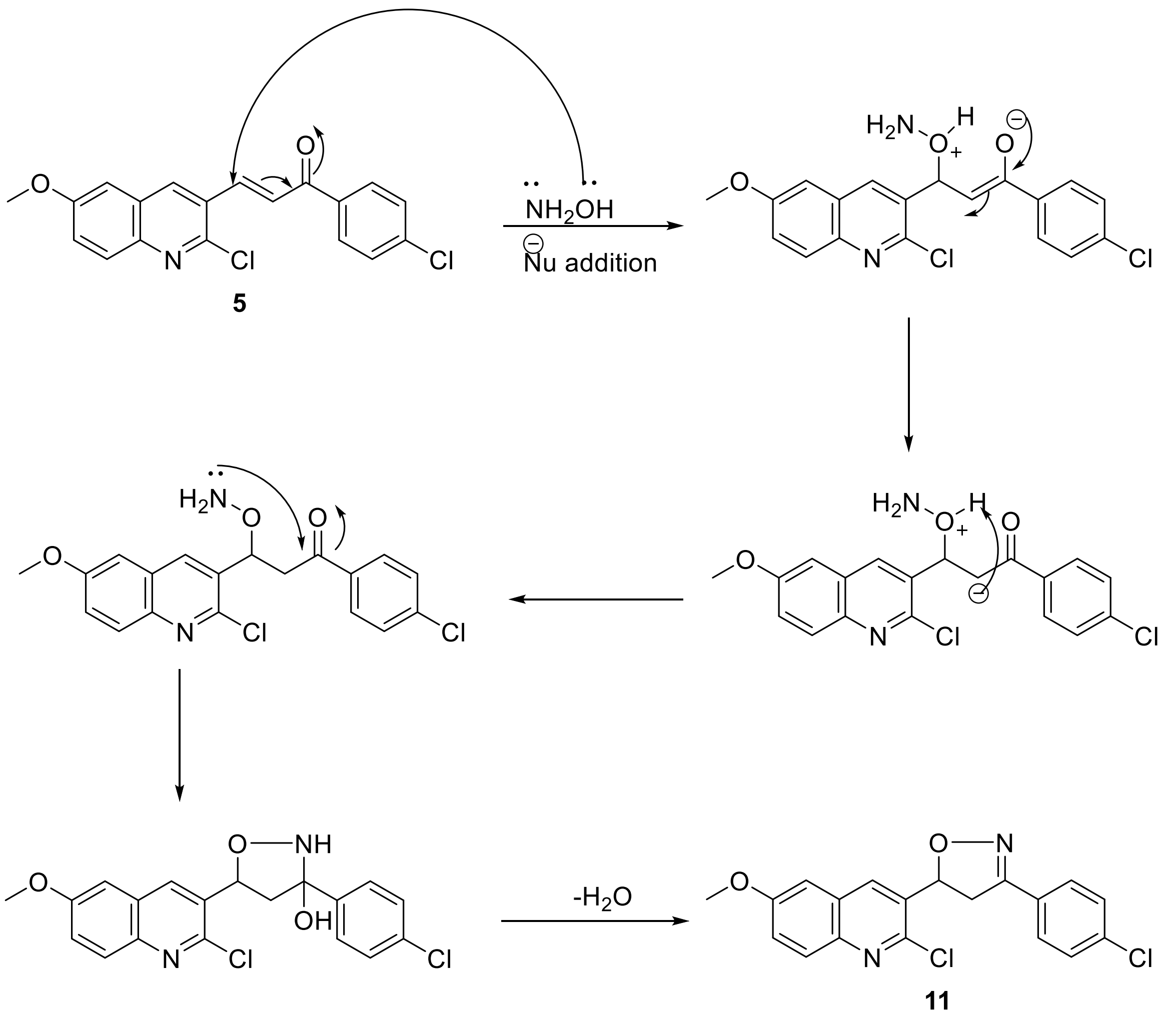

The proposed mechanism of reaction for formation of compounds containing pyrimidine

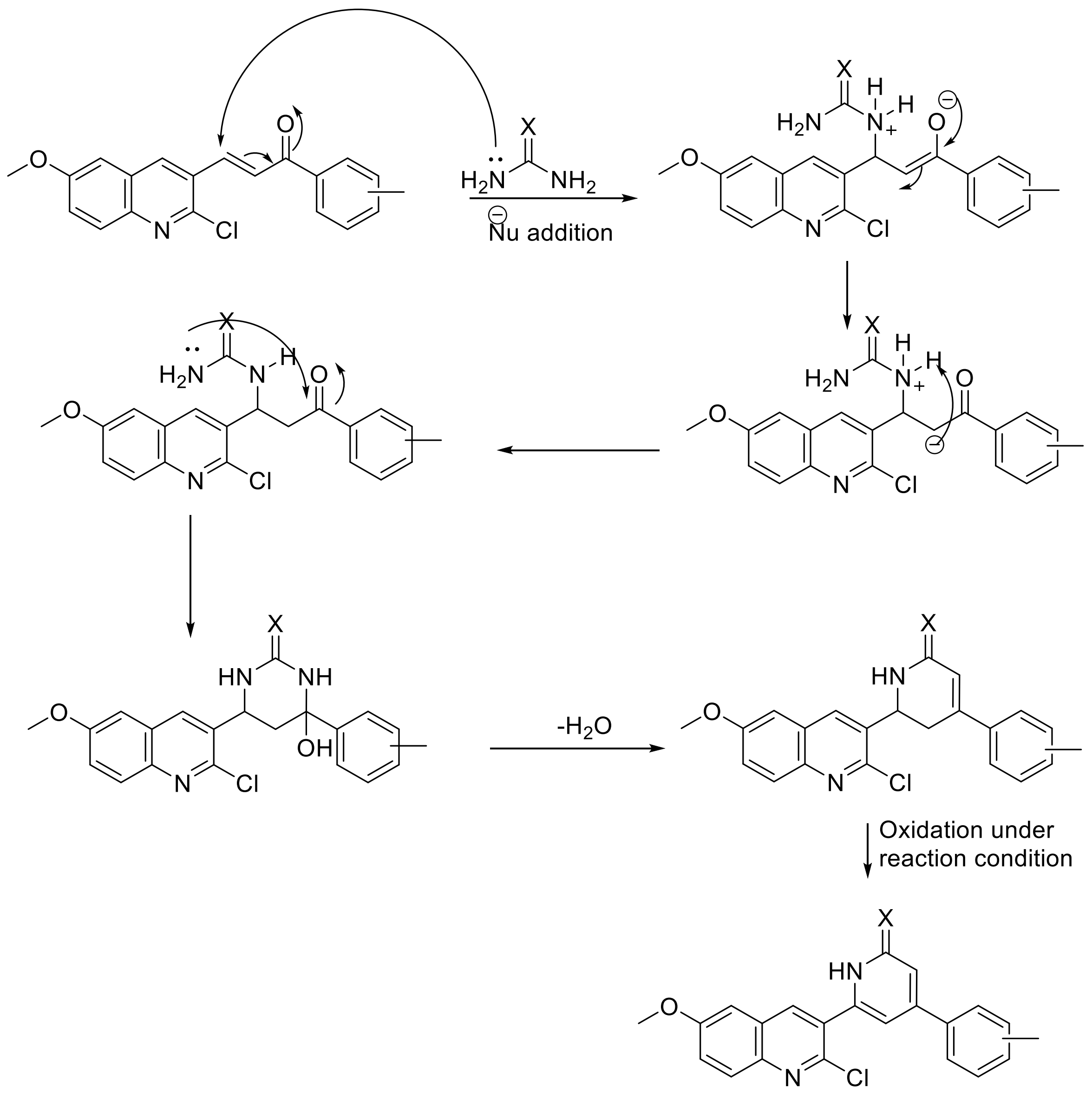
Nu addition
-H2O
Oxidation under
reaction condition

Dr_EmanYahia-ChoCH3

Sample Name	Dr_EmanYahia-Cho	Pulse sequence	PROTON	Temperature	26	Laboratory	MODCL
Date collected	2018-07-18	Solvent	DMSO	Spectrometer	nmr400-mercury400	NMR User	sameeh_Albadawy

Dr_EmanYahia-Cho

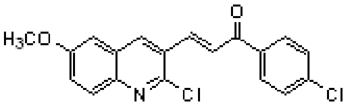

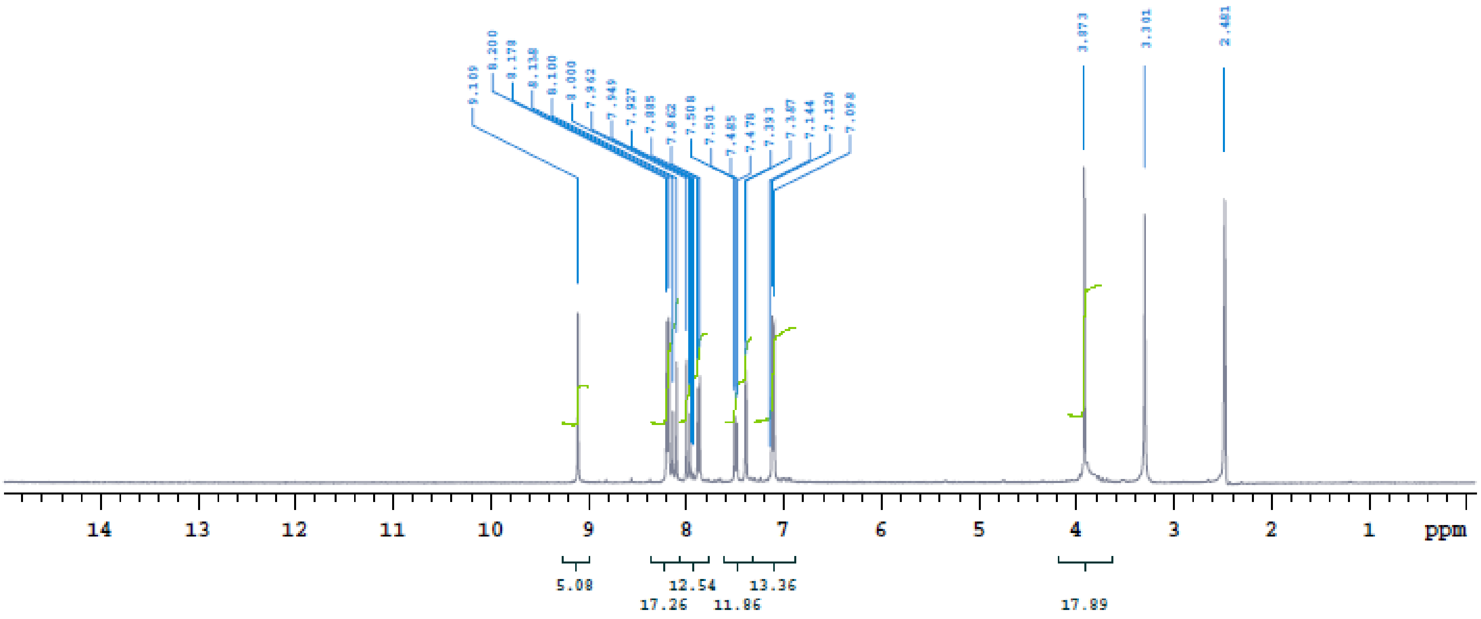

Plotname: Dr_EmanYahia-ChoCH3_PROTON_01_plot02

Data file /home/data/NMRlab2018/Jul/Dr_EmanYahia-ChoCH3_20180718_01/Dr_EmanYahia-ChoCH3_PROTON_01

Plot date 2018-07-18

Dr_EmanYahia-ChNH2

Sample Name Dr_EmanYahia-ChNH2	Pulse sequence PROTON	Temperature 25	Laboratory MODCL
Date collected 2018-07-18	Solvent DMSO	Spectrometer nmr400-mercury400	NMR User sameeh_Albadawy

Dr_EmanYahia-ChNH2

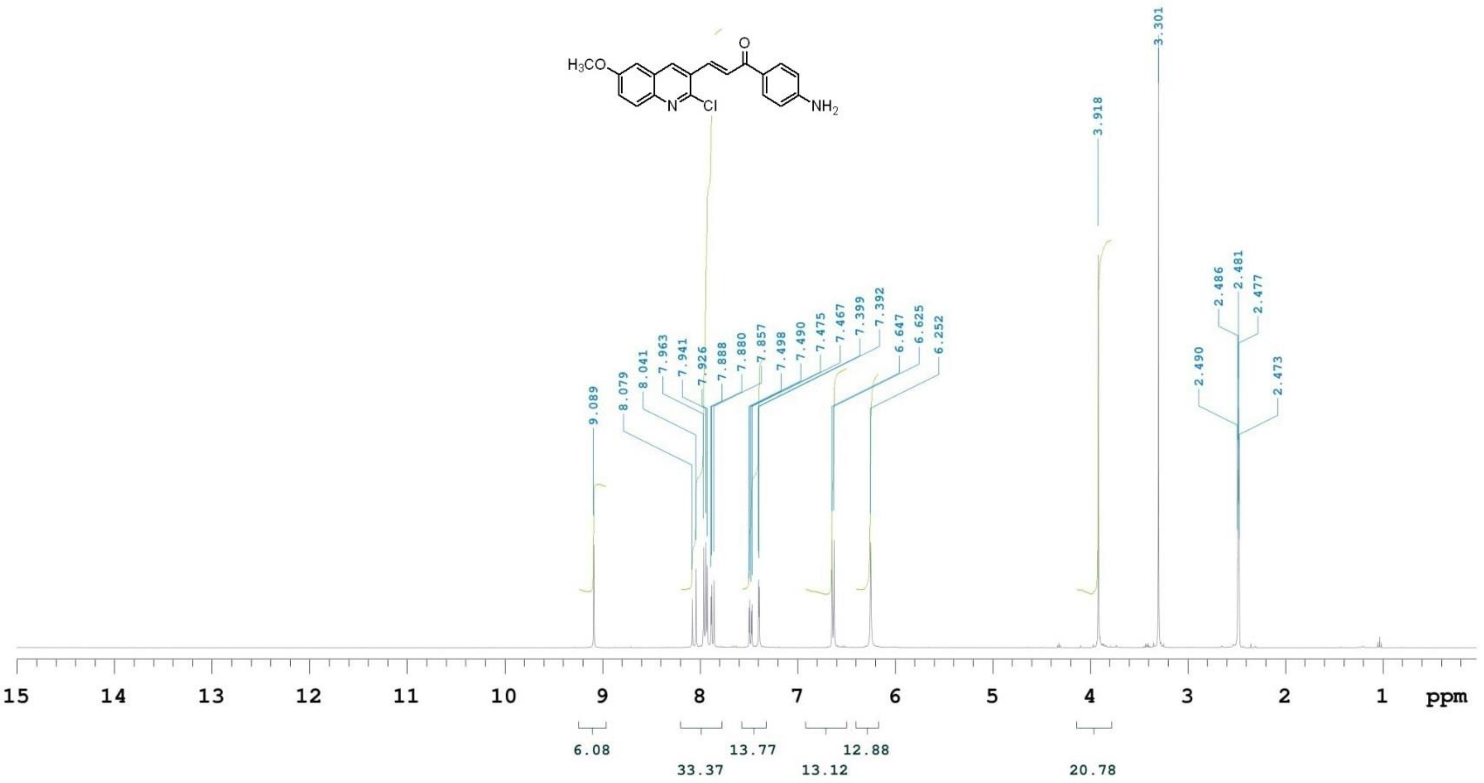

Plotname: Dr_EmanYahia-ChNH2_PROTON_01_plot02

Data file /home/data/NMRlab2018/Jul/Dr_EmanYahia-ChNH2_20180718_01/Dr_EmanYahia-ChNH2_PROTON_01

Plot date 2018-07-18

AbdolRahmanSalah-CA-DMSO-H1

Archive directory: /export/home/vnmr1/vnmrsys/data
Sample directory: DD5mm_test_12Mar2018-21:34:40
File: PROTON

Pulse Sequence: s2pul

Solvent: DMSO
Temp. 30.0 C / 303.1 K
Mercury-300BB "NMR300"

Relax. delay 1.000 sec
Pulse 45.0 degrees
Acq. time 4.853 sec
Width 6600.7 Hz
32 repetitions
OBSERVE H1, 300.0687865 MHz
DATA PROCESSING
FT size 65536
Total time 5 min, 16 sec
Date: Feb 5 2018

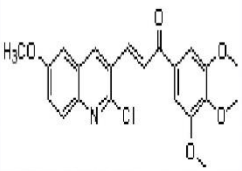

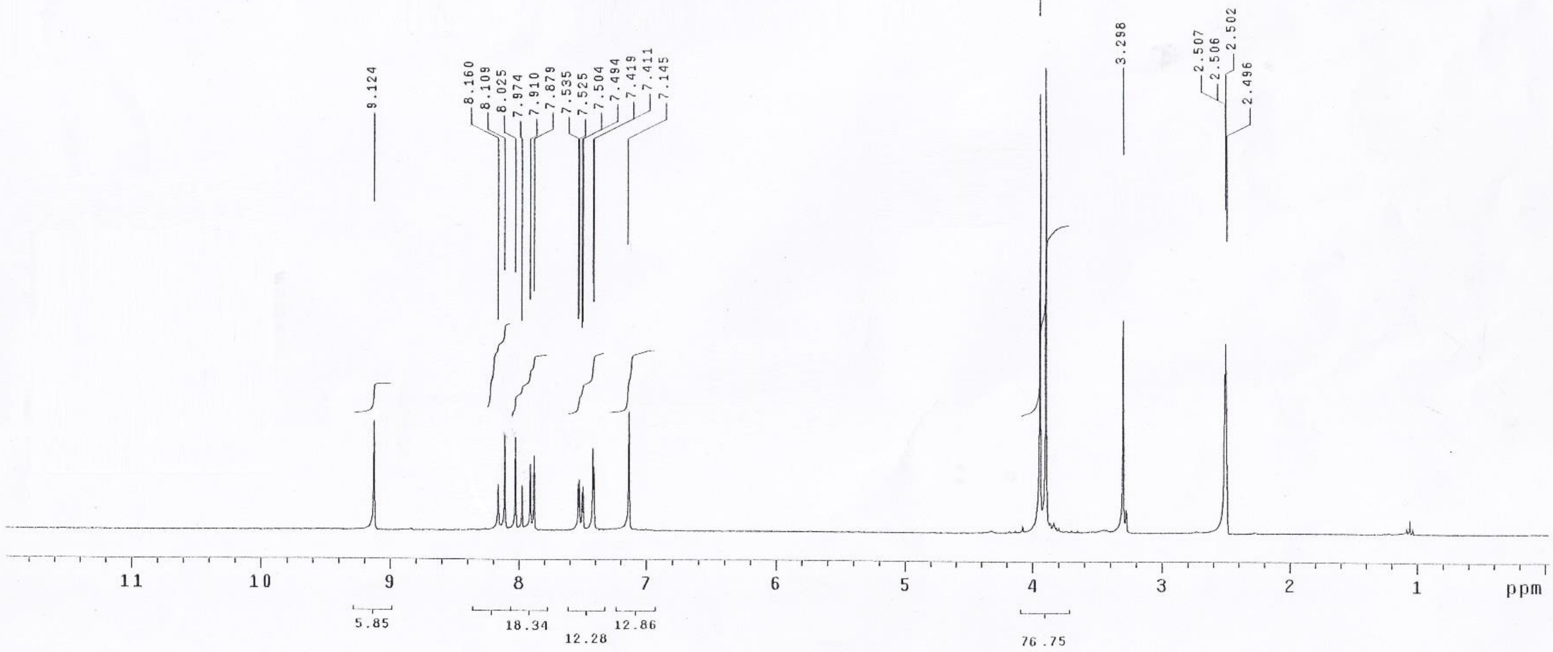

Dr_EmanYahia-ME1

Sample Name	Dr_EmanYahia-ME1	Pulse sequence	PROTON	Temperature	26	Laboratory	MODCL
Date collected	2018-07-18	Solvent	DMSO	Spectrometer	nmr400-mercury400	NMR User	sameeh_Albadawy

Dr_EmanYahia-ME1

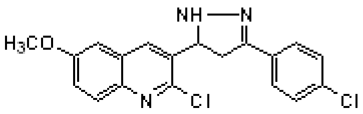

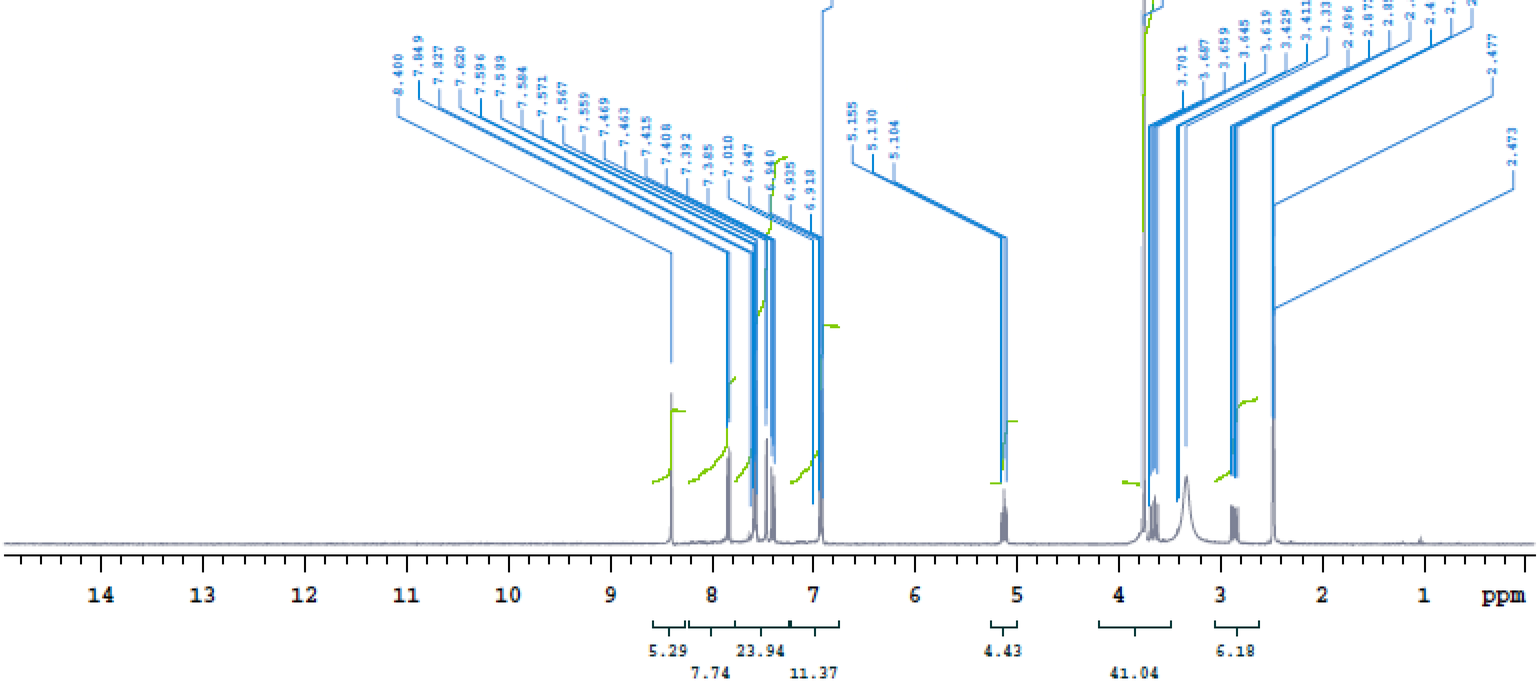

Plotname: Dr_EmanYahia-ME1_PROTON_01_plot03

Data file /home/data/NMRlab2018/Jul/Dr_EmanYahia-ME1_20180718_01/Dr_EmanYahia-ME1_PROTON_01

Plot date 2018-07-21

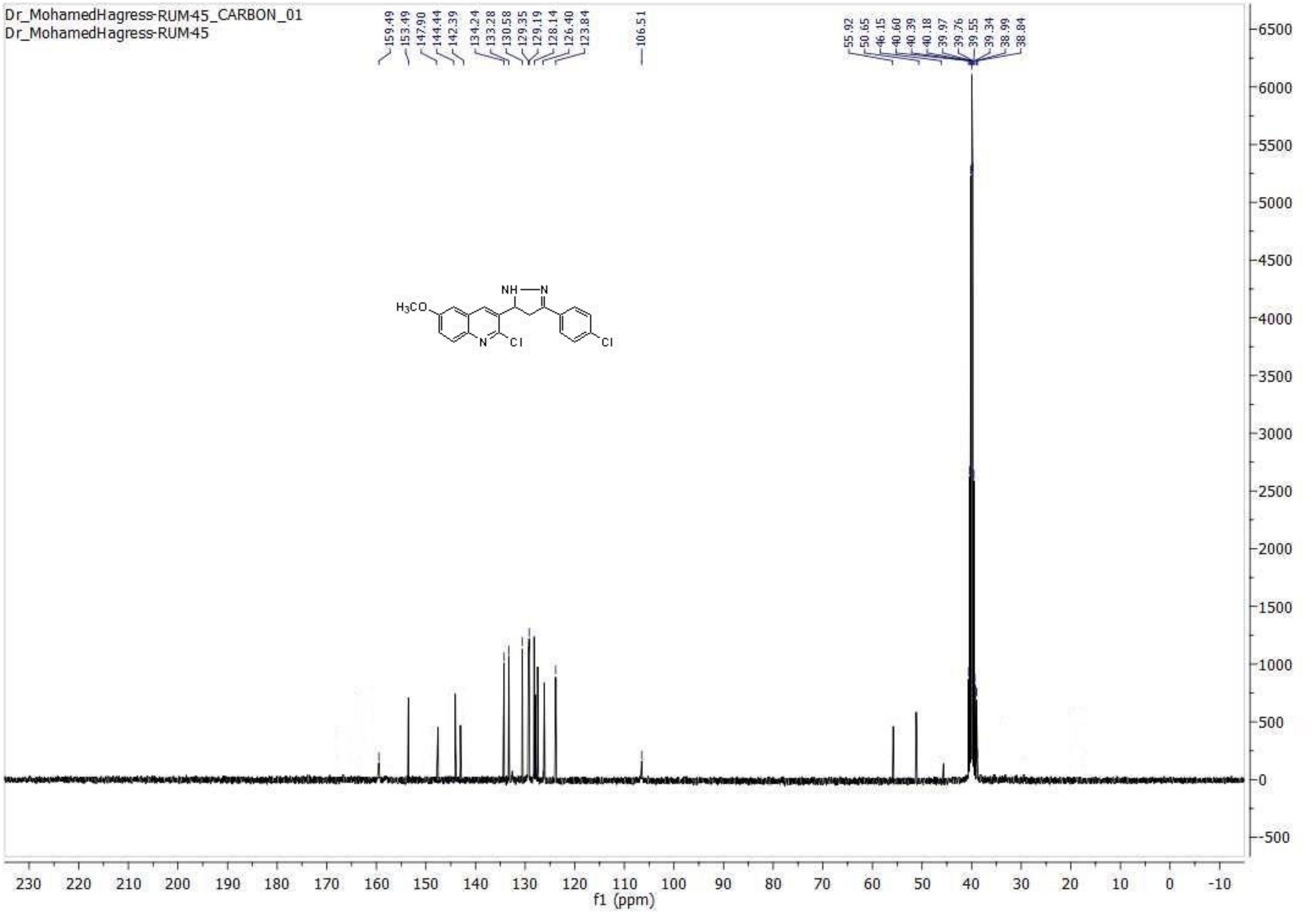

Dr_MohamedHagress-RUM45_CARBON_01
Dr_MohamedHagress-RUM45
159.49
153.49
147.90
144.44
142.39
134.24
133.28
130.58
129.35
129.19
128.14
126.40
123.84
106.51
55.92
50.65
46.15
40.60
40.39
40.18
39.97
39.76
39.55
39.34
38.99
38.84
H_3CO
NH
N
N
Cl
Cl
f1 (ppm)

Dr_EmanYahia-ME8

Sample Name Dr_EmanYahia-ME8	Pulse sequence PROTON	Temperature 25	Laboratory MODCL
Date collected 2018-07-18	Solvent DMSO	Spectrometer nmr400-mercury400	NMR User sameeh_Albadawy

Dr_EmanYahia-ME8

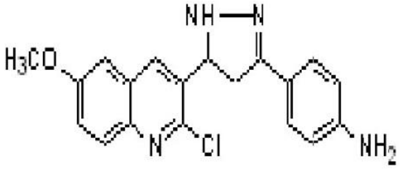

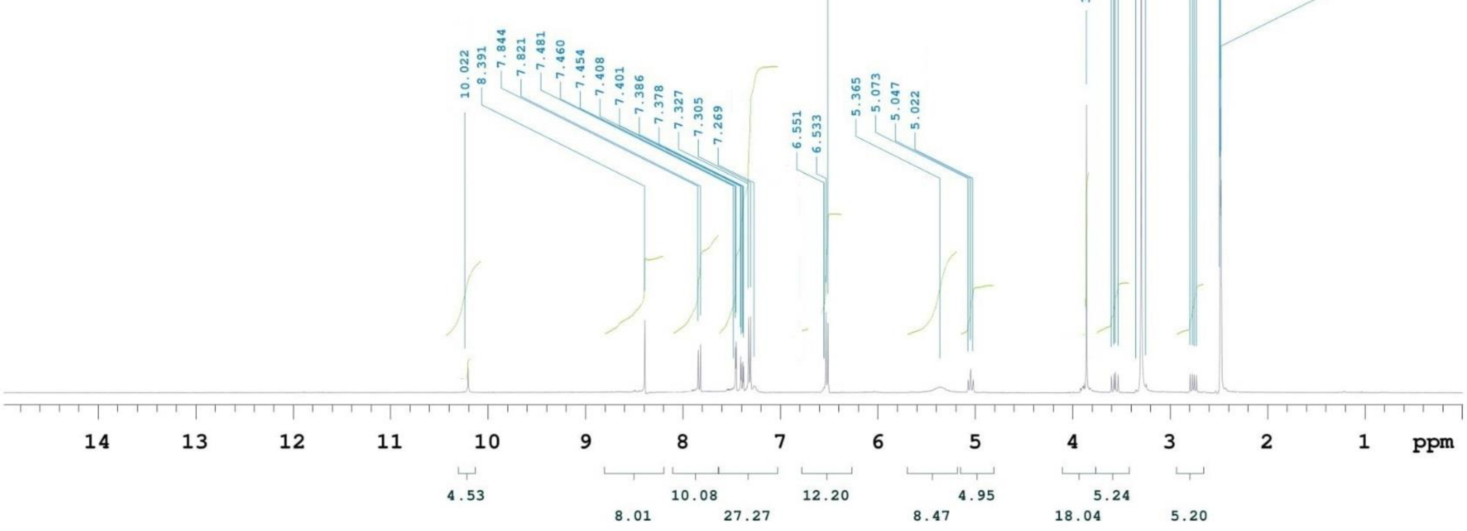

Plotname: Dr_EmanYahia-ME8_PROTON_01_plot02

Data file /home/data/NMRlab2018/Jul/Dr_EmanYahia-ME8_20180718_01/Dr_EmanYahia-ME8_PROTON_01

Plot date 2018-07-18

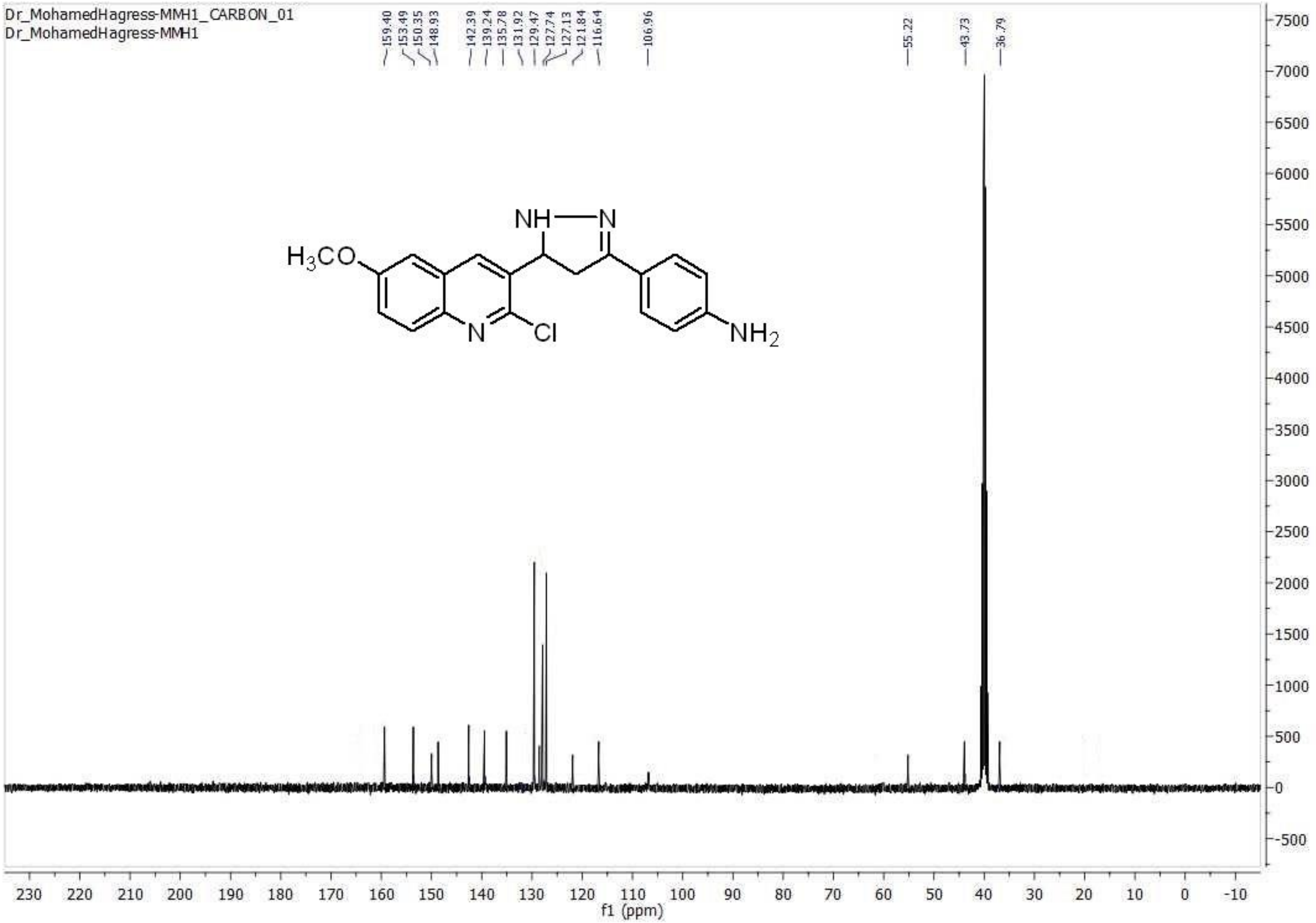
Dr_MohamedHagress-MMH1_CARBON_01
Dr_MohamedHagress-MMH1
159.40
153.49
150.35
148.93
142.39
139.24
135.78
131.92
129.47
127.74
127.13
121.84
116.64
106.96
55.22
43.73
36.79
H3CO
NH
N
N
Cl
NH2
f1 (ppm)

H1-proton

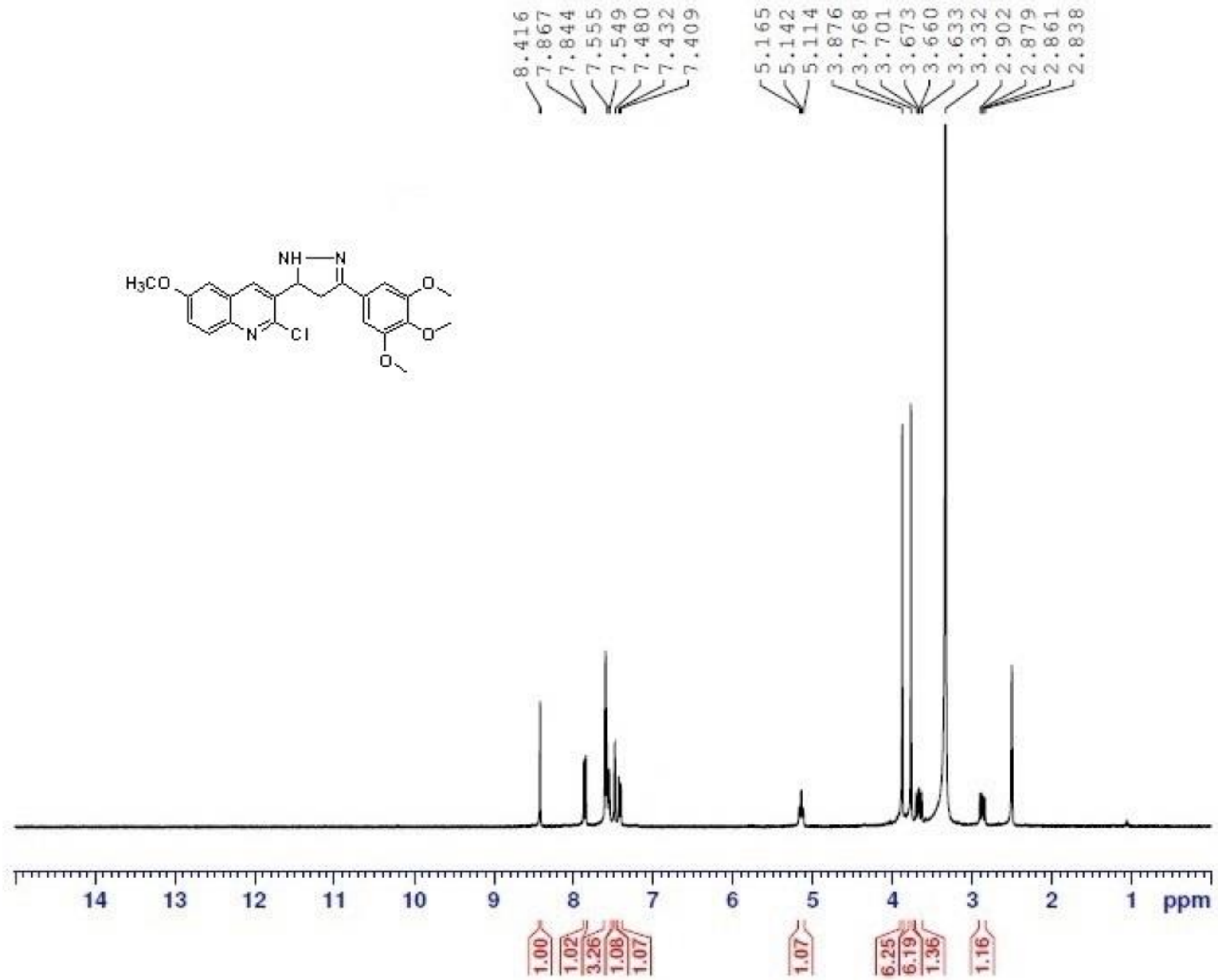

H_3CO
NH
N
N
Cl
O
8.416
7.867
7.844
7.555
7.549
7.480
7.432
7.409
5.165
5.142
5.114
3.876
3.768
3.701
3.673
3.660
3.633
3.332
2.902
2.879
2.861
2.838
14 13 12 11 10 9 8 7 6 5 4 3 2 1 ppm
1.00
1.02
3.26
1.08
1.07
1.07
6.25
6.19
1.36
1.16

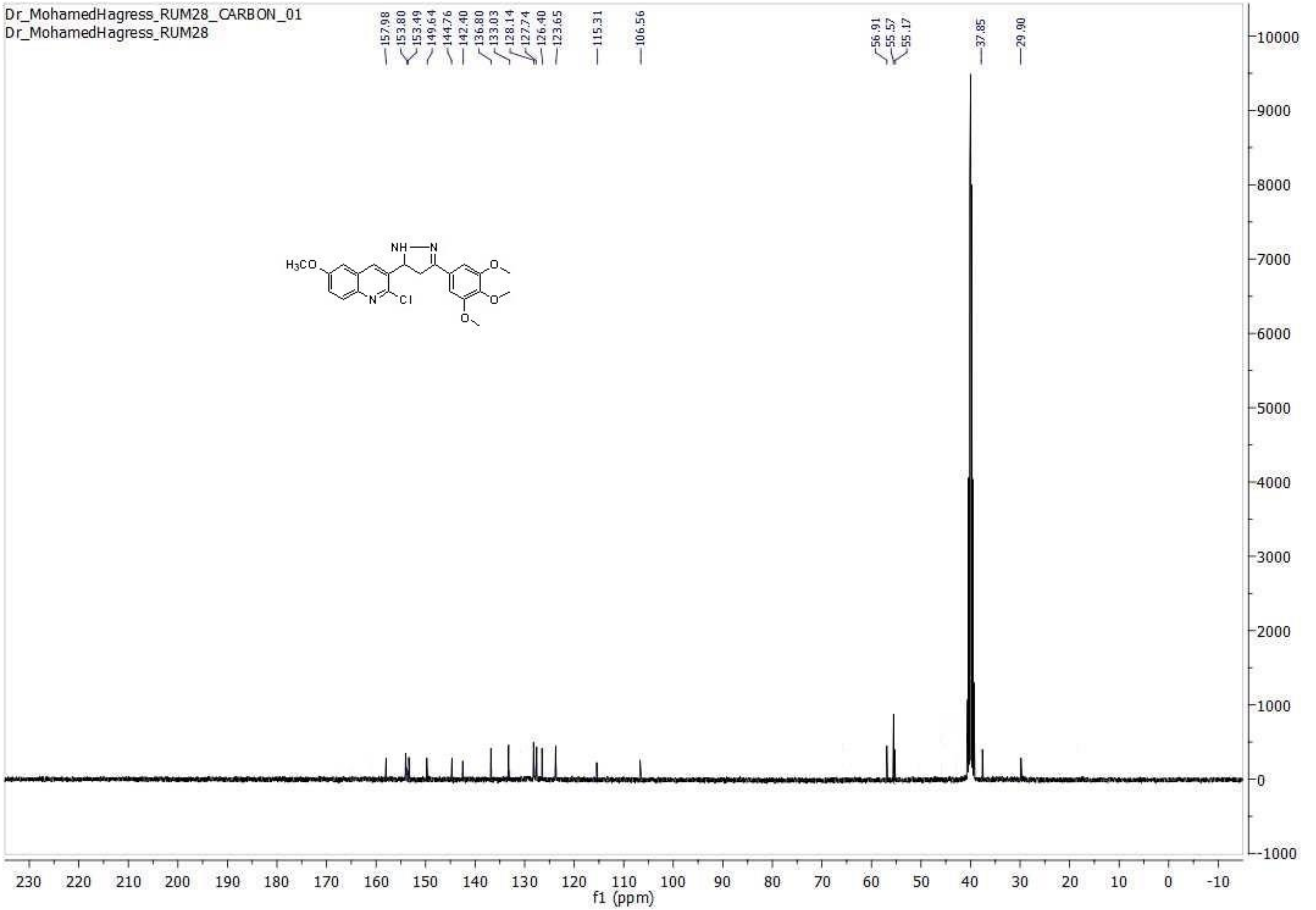
Dr_MohamedHagress_RUM28_CARBON_01
Dr_MohamedHagress_RUM28
157.98
153.80
153.49
149.64
144.76
142.40
136.80
133.03
128.14
127.74
126.40
123.65
115.31
106.56
56.91
55.57
55.17
37.85
29.90
f1 (ppm)

Dr_EmanYahia-ME2

Sample Name	Dr_EmanYahia-ME2	Pulse sequence	PROTON	Temperature	26	Laboratory	MODCL
Date collected	2018-07-18	Solvent	DMSO	Spectrometer	nmr400-mercury400	NMR User	sameeh_Albadawy

Dr_EmanYahia-ME2

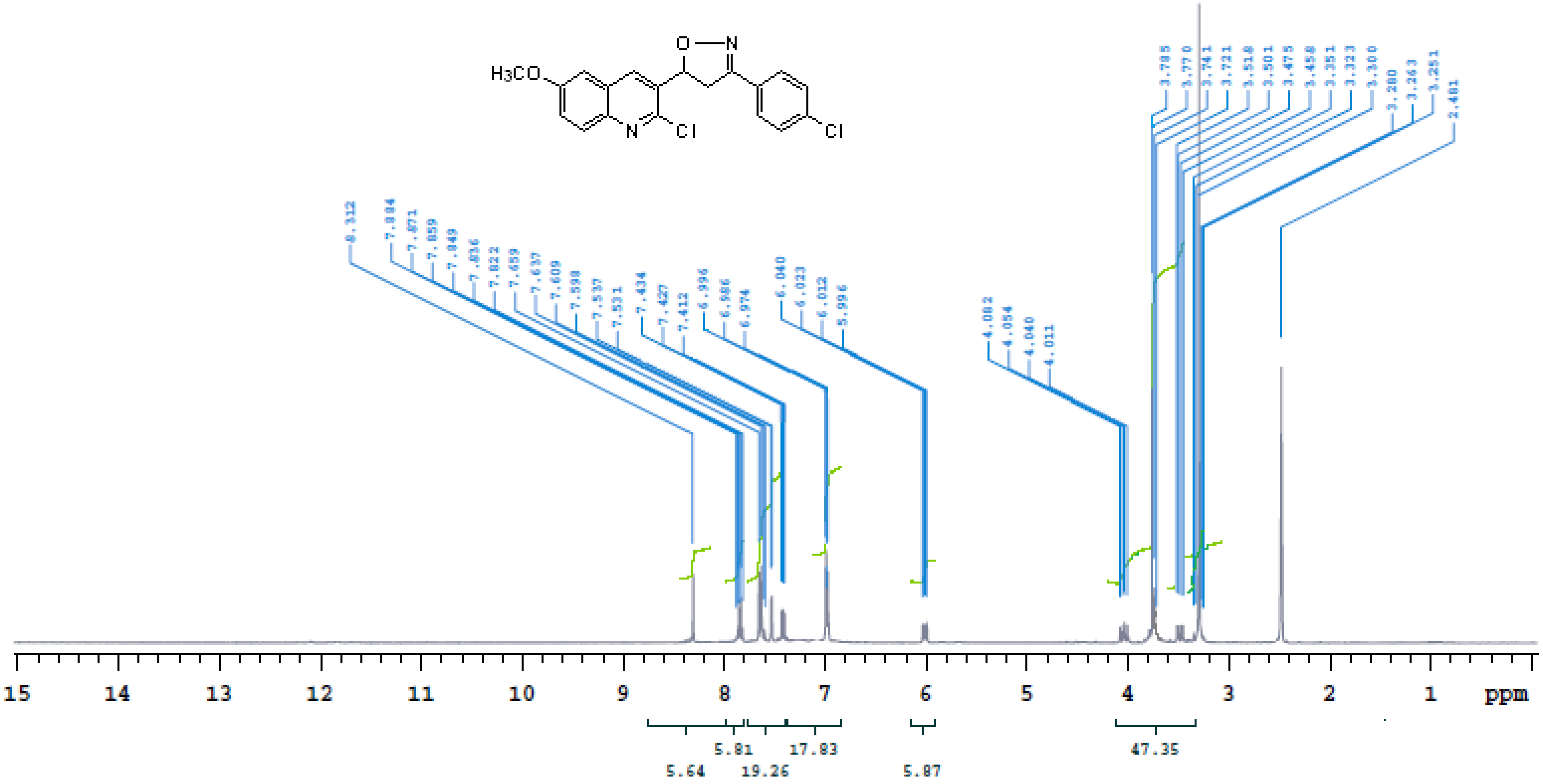

Plotname: Dr_EmanYahia-ME2_PROTON_01_plot05

Data file /home/data/NMRlab2018/Jul/Dr_EmanYahia-ME2_20180718_01/Dr_EmanYahia-ME2_PROTON_01

Plot date 2018-07-21

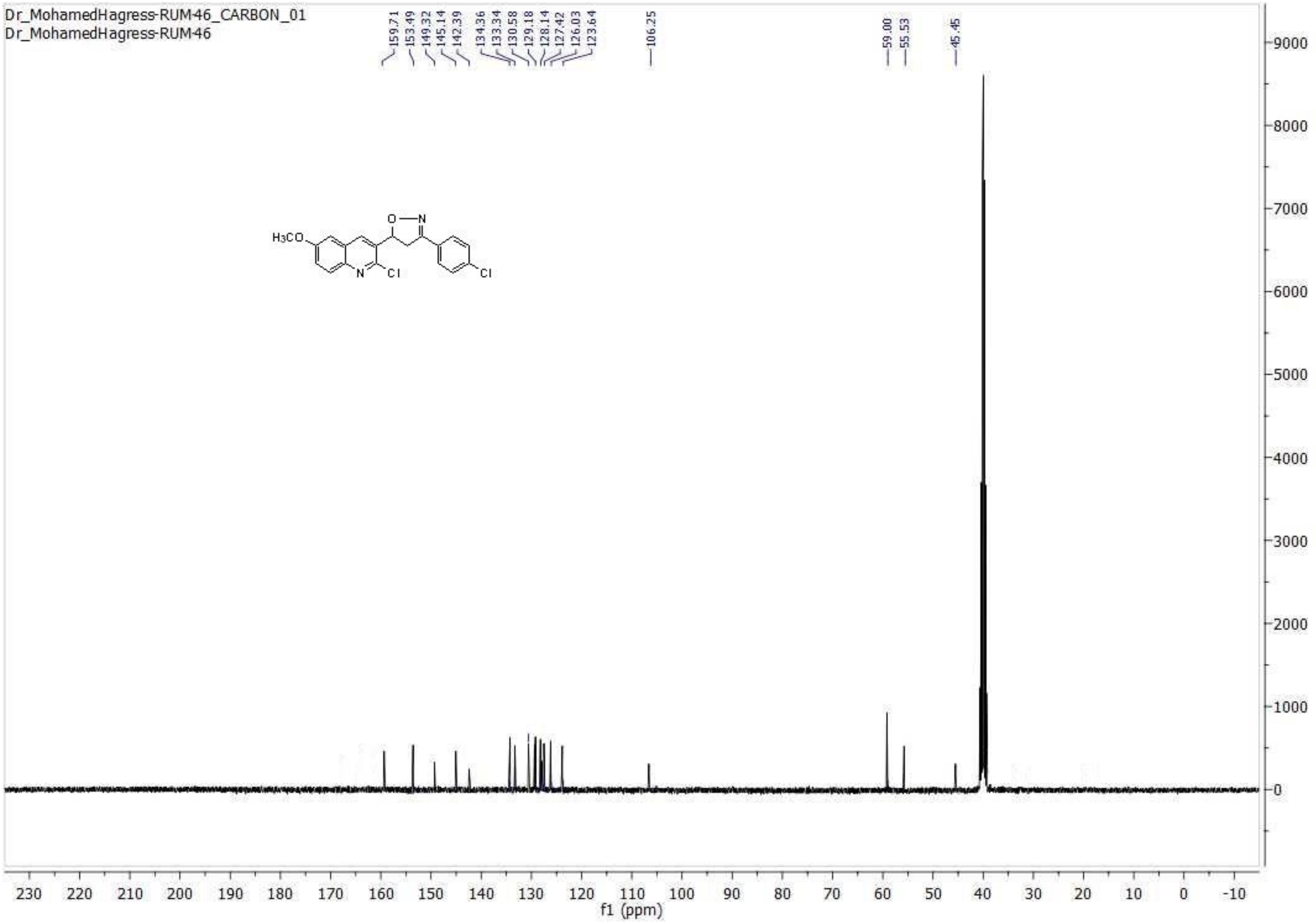
Dr_MohamedHagress-RUM46_CARBON_01
Dr_MohamedHagress-RUM46
159.71
153.49
149.32
145.14
142.39
134.36
133.34
130.58
129.18
128.14
127.42
126.03
123.64
106.25
59.00
55.53
45.45
H3CO
O
N
N
Cl
Cl
9000
8000
7000
6000
5000
4000
3000
2000
1000
0
230
220
210
200
190
180
170
160
150
140
130
120
110
100
90
80
70
60
50
40
30
20
10
0
-10
f1 (ppm)

Dr_EmanYahia-ME7

Sample Name	Dr_EmanYahia-ME7	Pulse sequence	PROTON	Temperature	25	Laboratory	MODCL
Date collected	2018-07-18	Solvent	DMSO	Spectrometer	nmr400-mercury400	NMR User	sameeh_Albadawy

Dr_EmanYahia-ME7

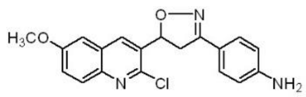

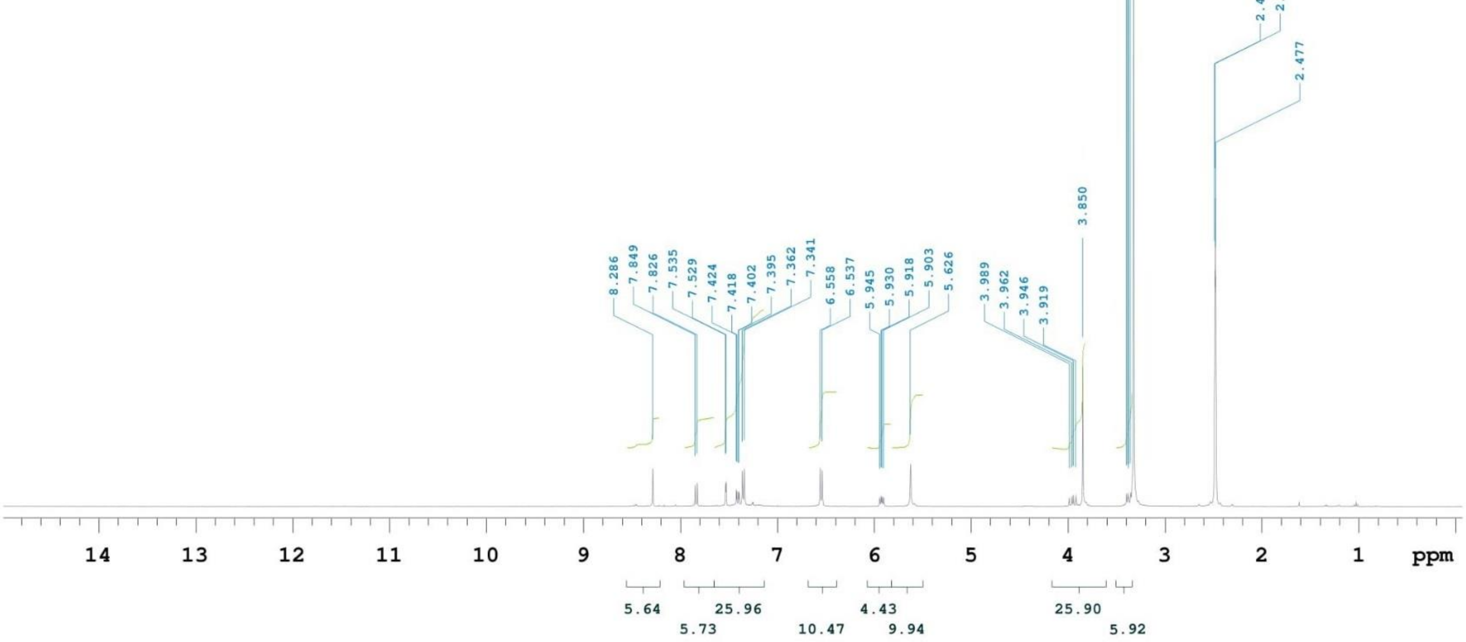

Plotname: Dr_EmanYahia-ME7_PROTON_01_plot02

Data file /home/data/NMRlab2018/Jul/Dr_EmanYahia-ME7_20180718_01/Dr_EmanYahia-ME7_PROTON_01

Plot date 2018-07-18

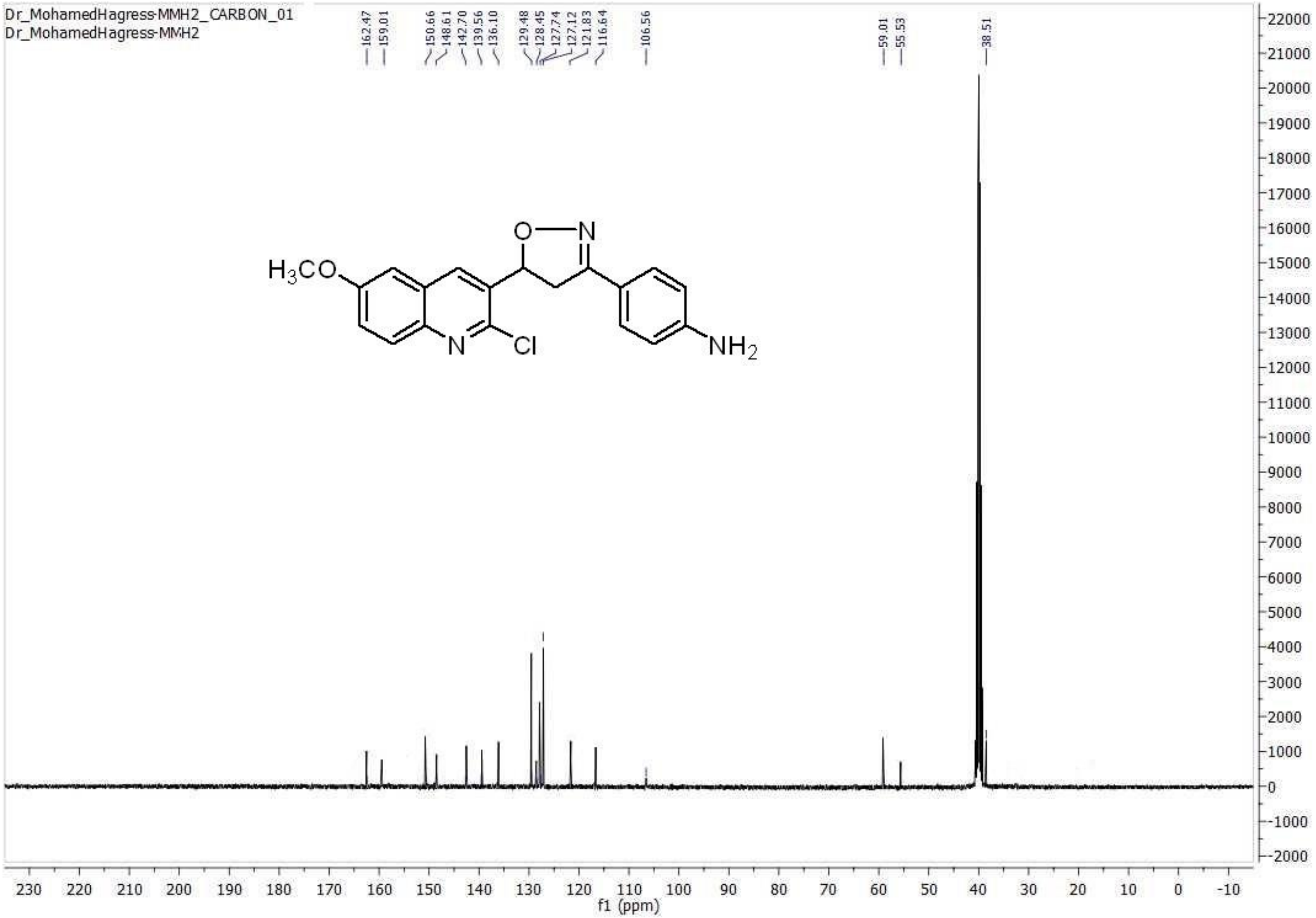

Dr_MohamedHagress-MMH2_CARBON_01
Dr_MohamedHagress-MMH2
162.47
159.01
150.66
148.61
142.70
139.56
136.10
129.48
128.45
127.74
127.12
121.83
116.64
106.56
59.01
55.53
38.51
H3CO
O
N
N
Cl
NH2
f1 (ppm)

Mohamed Samy-CA3-DMSO-H1

Archive directory: /export/home/vnmr1/vnmrsys/data
Sample directory: DD5mm_test_12Mar2019-21:34:40
File: PROTON

Pulse Sequence: s2pul

Solvent: DMSO
Temp. 30.0 C / 303.1 K
Mercury-300BB "NMR300"

Relax. delay 1.000 sec
Pulse 45.0 degrees
Acq. time 4.853 sec
Width 6600.7 Hz
12 repetitions
OBSERVE H1, 300.0687865 MHz
DATA PROCESSING
FT size 65536
Total time 5 min, 16 sec
Date: Feb 5 2019

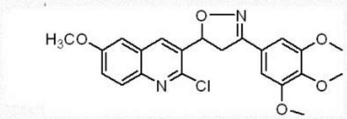

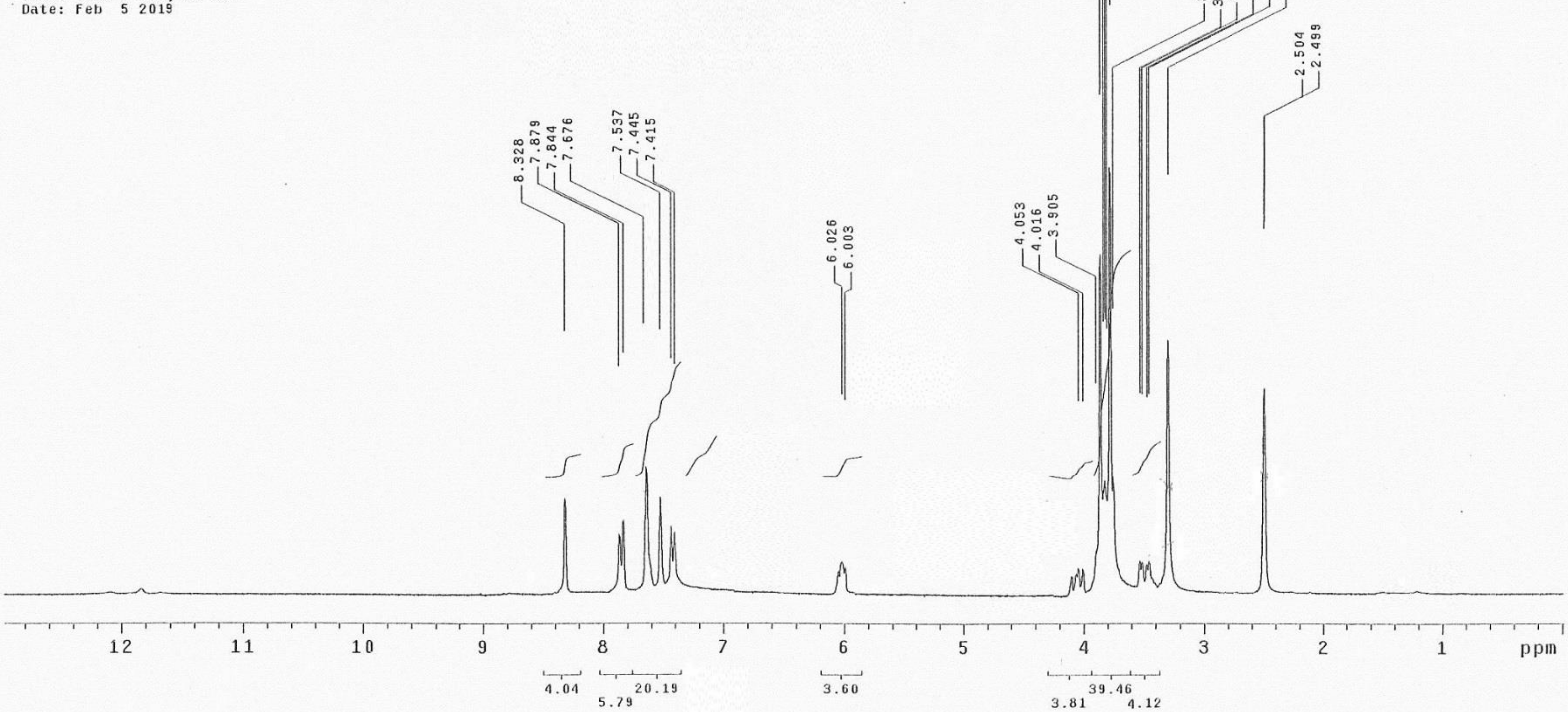

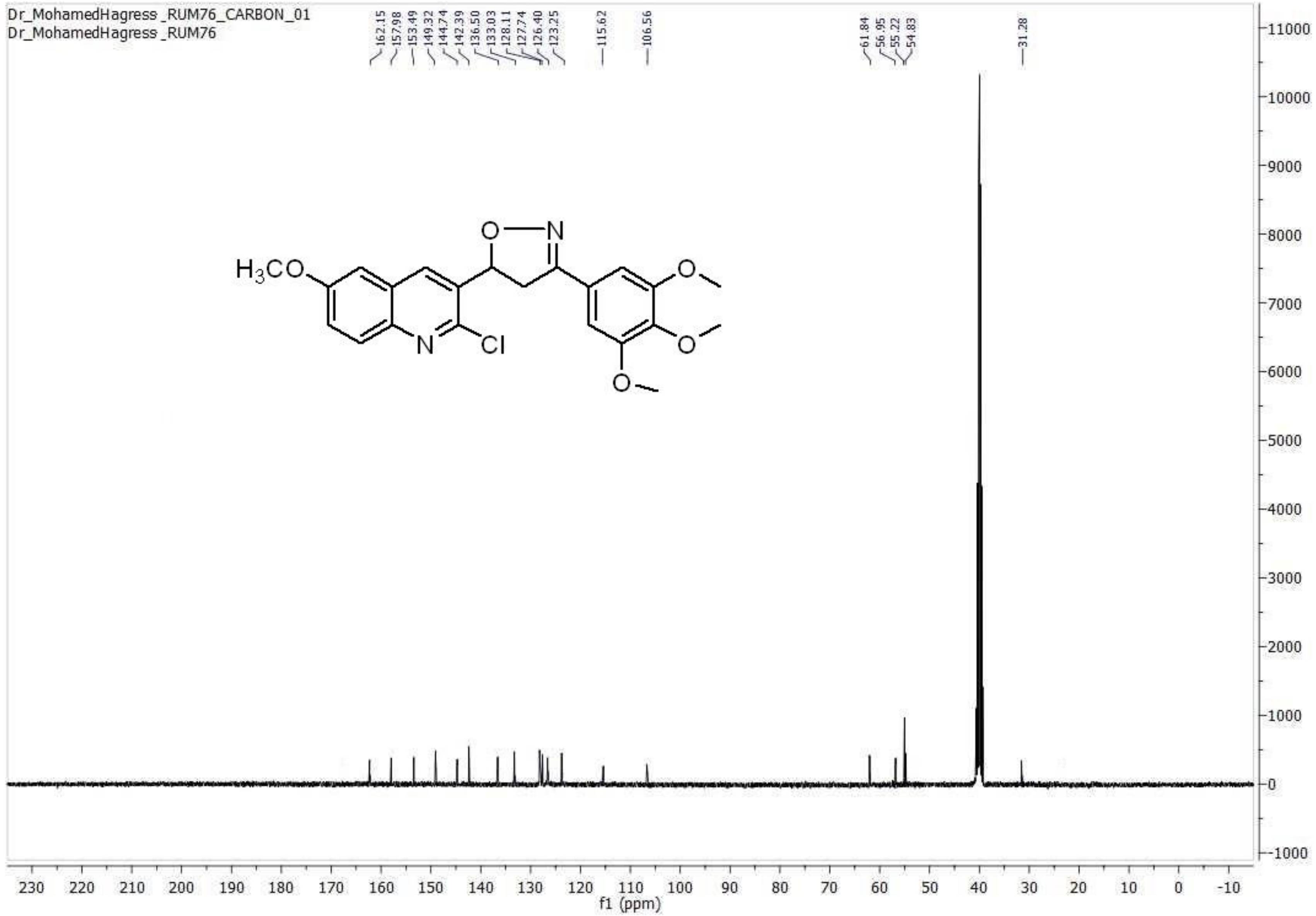

Dr_MohamedHagress _RUM76_CARBON_01
Dr_MohamedHagress _RUM76
162.15
157.98
153.49
149.32
144.74
142.39
136.50
133.03
128.11
127.74
126.40
123.25
115.62
106.56
61.84
56.95
55.22
54.83
31.28
H3CO
O
N
Cl
f1 (ppm)

Dr_MohammedHagress-Ruk26

Dr_MohammedHagress-Ruk26

Sample Name **Dr_MohammedHagress-Ruk26**	Pulse sequence **PROTON**	Temperature **26**	Study owner **vnmr1**
Date collected **2017-11-12**	Solvent **dmso**	Spectrometer **nmr400-mercury400**	Operator **vnmr1**

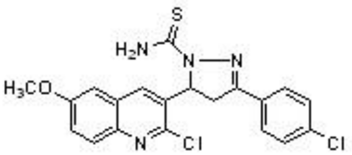

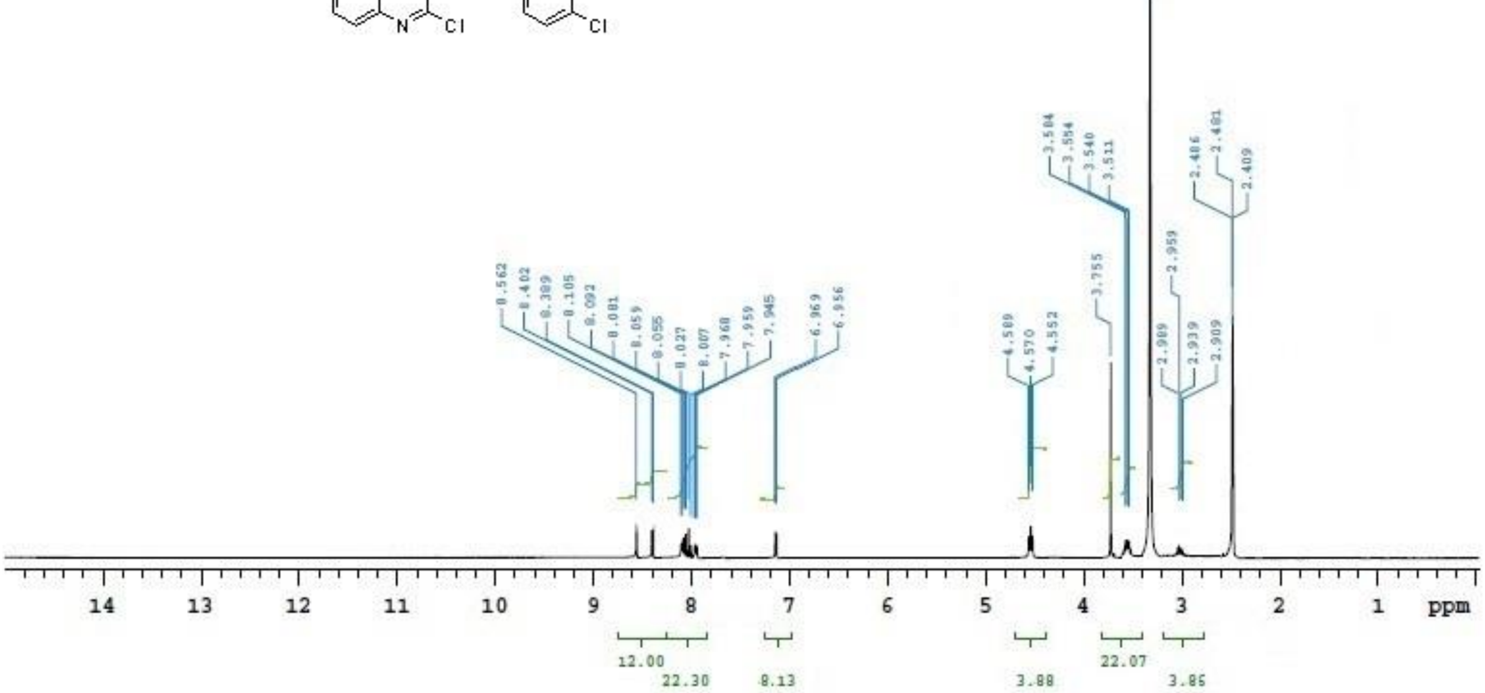

Plotname: Dr_MohammedHagress-Ruk26_PROTON_01_plot02

Data file /home/data/NMRlab2017/Nov/Dr_MohammedHagress-Ruk26_20171112_01/Dr_MohammedHagress-Ruk26_PROTON_01

Plot date 2017-11-12

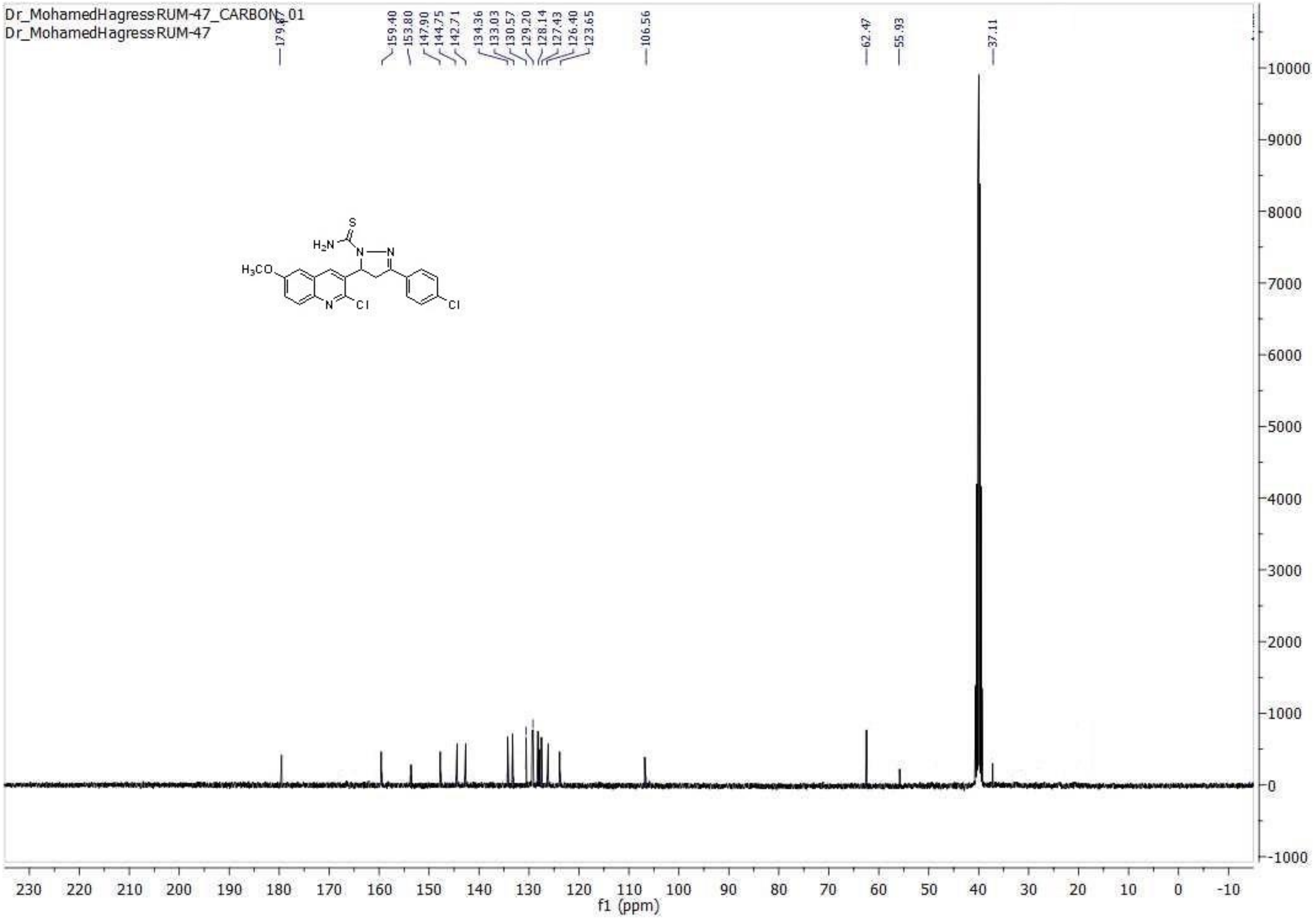
Dr_MohamedHagressRUM-47_CARBON_01
Dr_MohamedHagressRUM-47
159.40
153.80
147.90
144.75
142.71
134.36
133.03
130.57
129.20
128.14
127.43
126.40
123.65
106.56
62.47
55.93
37.11
S
H2N
N
N
H3CO
N
Cl
Cl
f1 (ppm)

Dr_EmanYahia-ME-1b

Dr_EmanYahia-ME-1b

Sample Name Dr_EmanYahia-ME-1b	Pulse sequence PROTON	Temperature 25	Laboratory MODCL
Date collected 2018-03-19	Solvent DMSO	Spectrometer nmr400-mercury400	NMR User

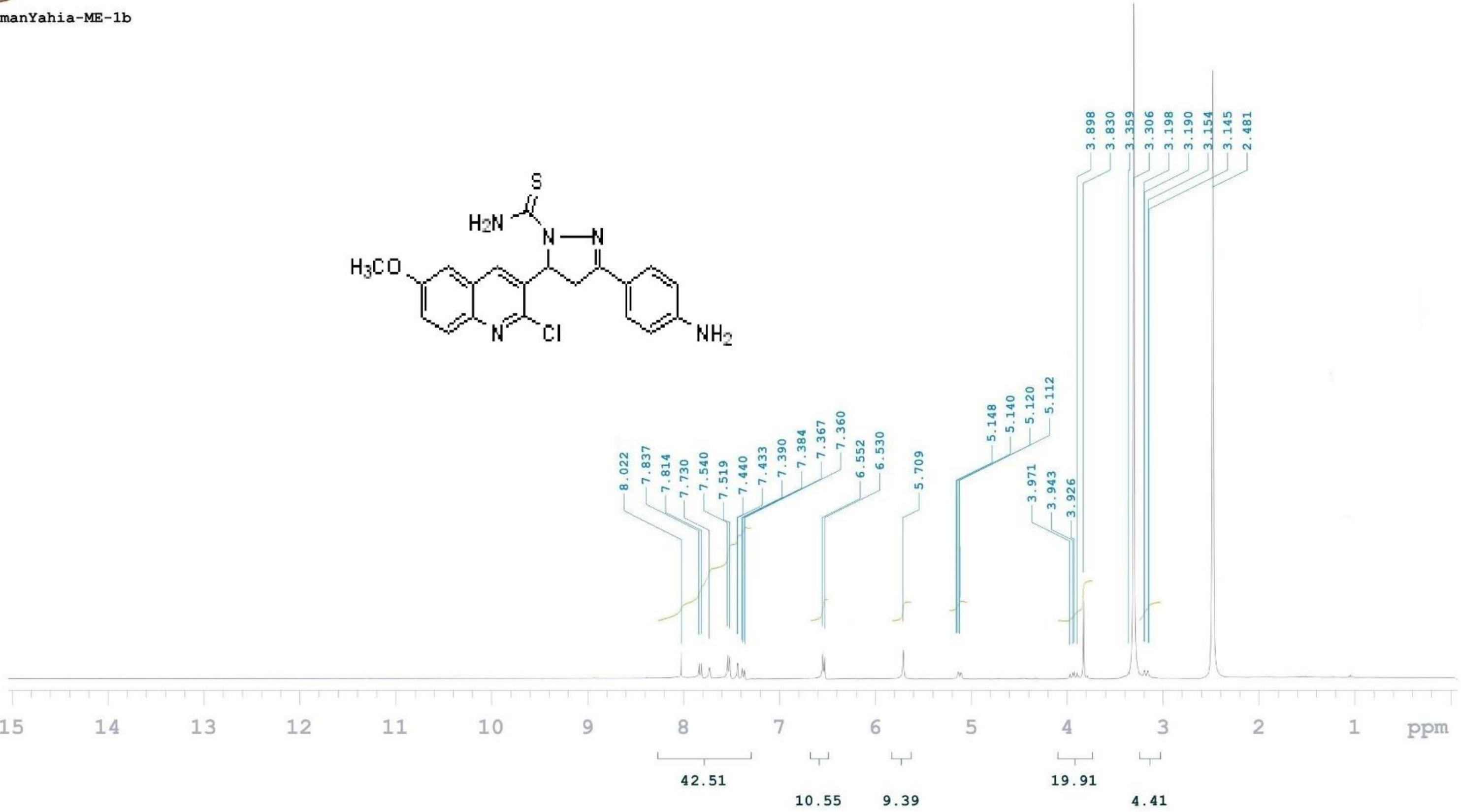

Plotname: Dr_EmanYahia-ME-1b_PROTON_01_plot02

Data file /home/data/NMRlab2018/Mar/Dr_EmanYahia-ME-1b_20180319_01/Dr_EmanYahia-ME-1b_PROTON_01

Plot date 2018-03-19

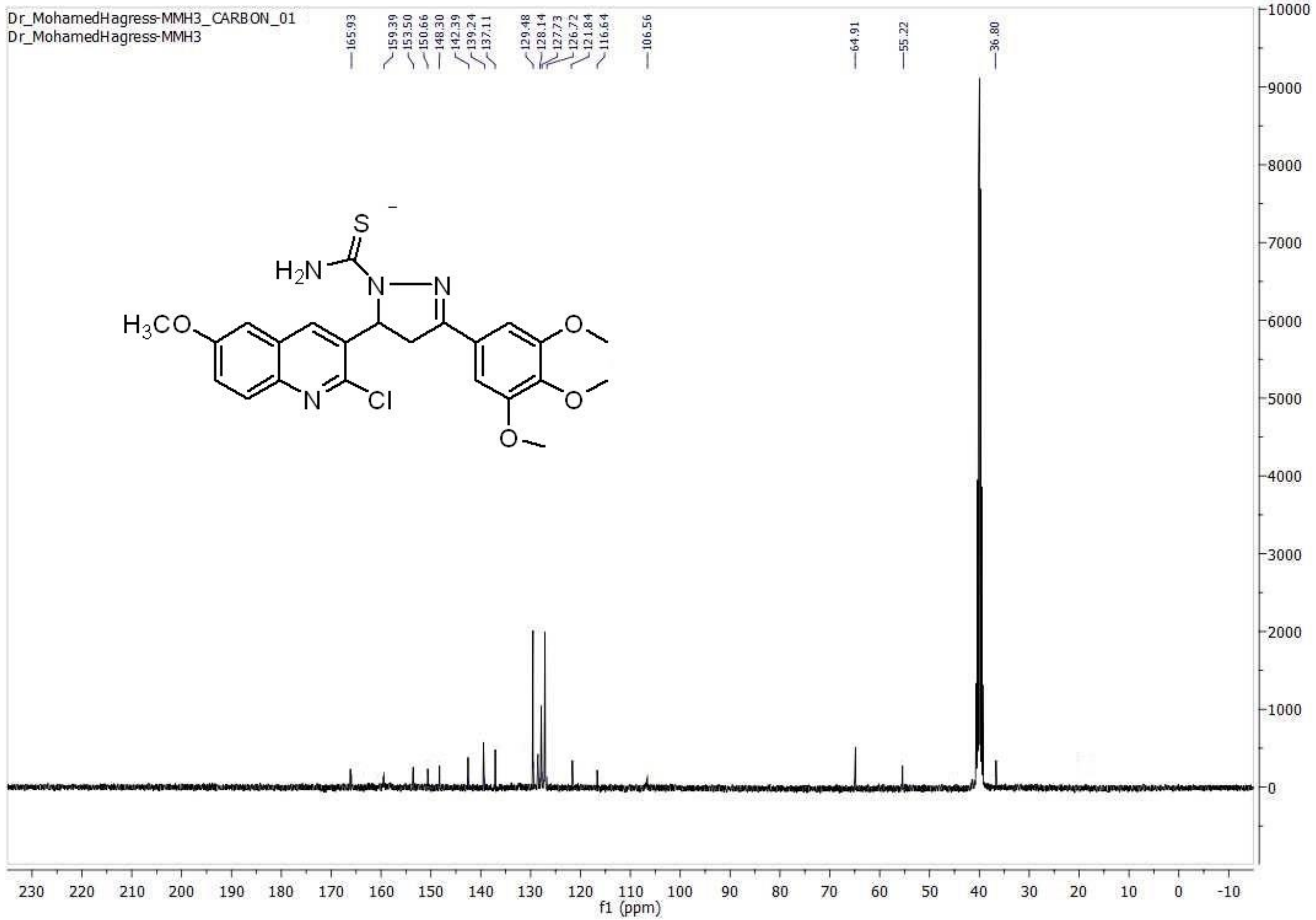

Dr_MohamedHagress-MMH3_CARBON_01
Dr_MohamedHagress-MMH3
165.93
159.39
153.50
150.66
148.30
142.39
139.24
137.11
129.48
128.14
127.73
126.72
121.84
116.64
106.56
64.91
55.22
36.80
S
H2N
N
N
H3CO
N
Cl
O
O
O
f1 (ppm)

H1- 2

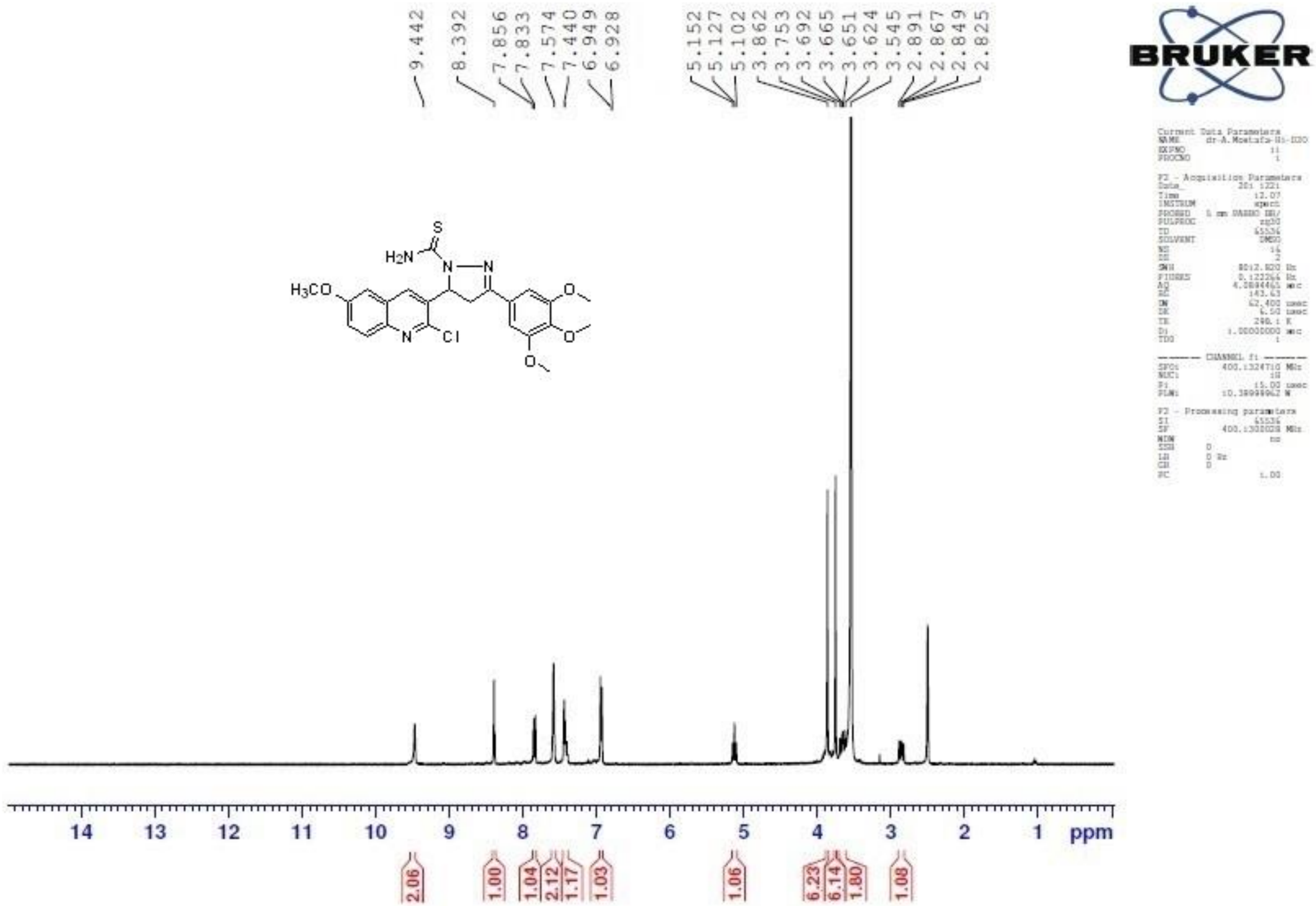
9.442
8.392
7.856
7.833
7.574
7.440
6.949
6.928
5.152
5.127
5.102
3.862
3.753
3.692
3.665
3.651
3.624
3.545
2.891
2.867
2.849
2.825
BRUKER
Current Data Parameters
F2 - Acquisition Parameters
CHANNEL f1
F2 - Processing parameters
S
H2N
N
N
H3CO
N
Cl
O
O
O
14
13
12
11
10
9
8
7
6
5
4
3
2
1
ppm
2.06
1.00
1.04
2.12
1.17
1.03
1.06
6.23
6.14
1.80
1.08

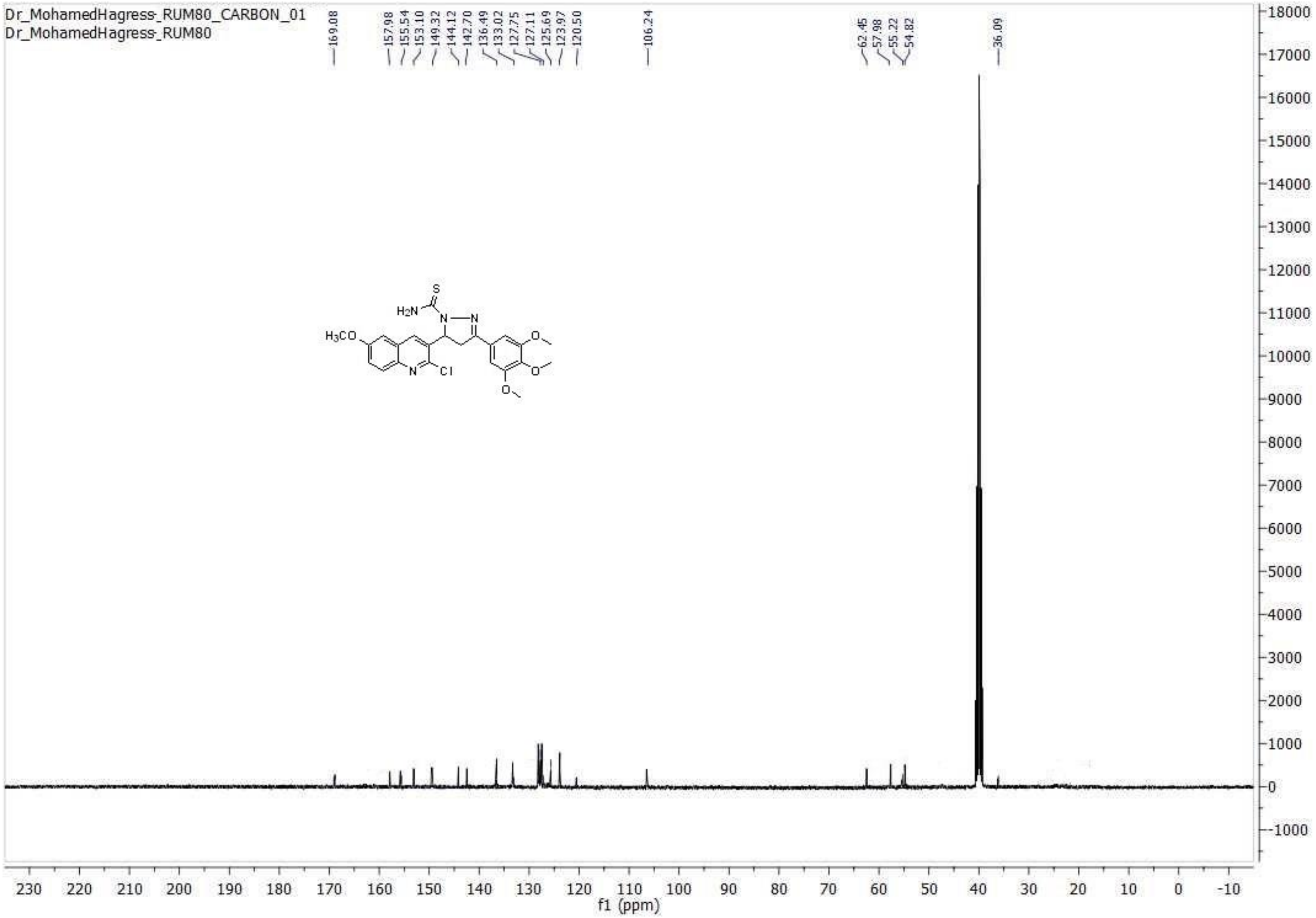
Dr_MohamedHagress_RUM80_CARBON_01
Dr_MohamedHagress_RUM80
169.08
157.98
155.54
153.10
149.32
144.12
142.70
136.49
133.02
127.75
127.11
125.69
123.97
120.50
106.24
62.45
57.98
55.22
54.82
36.09
H2N
S
N
N
H3CO
N
Cl
O
O
O
f1 (ppm)

Dr_MohammedHagress-Ruk28

Dr_MohammedHagress-Ruk28

Sample Name	Dr_MohammedHagress-Ruk28	Pulse sequence	PROTON	Temperature	26	Study owner	vnmr1
Date collected	2017-11-12	Solvent	dmso	Spectrometer	nmr400-mercury400	Operator	vnmr1

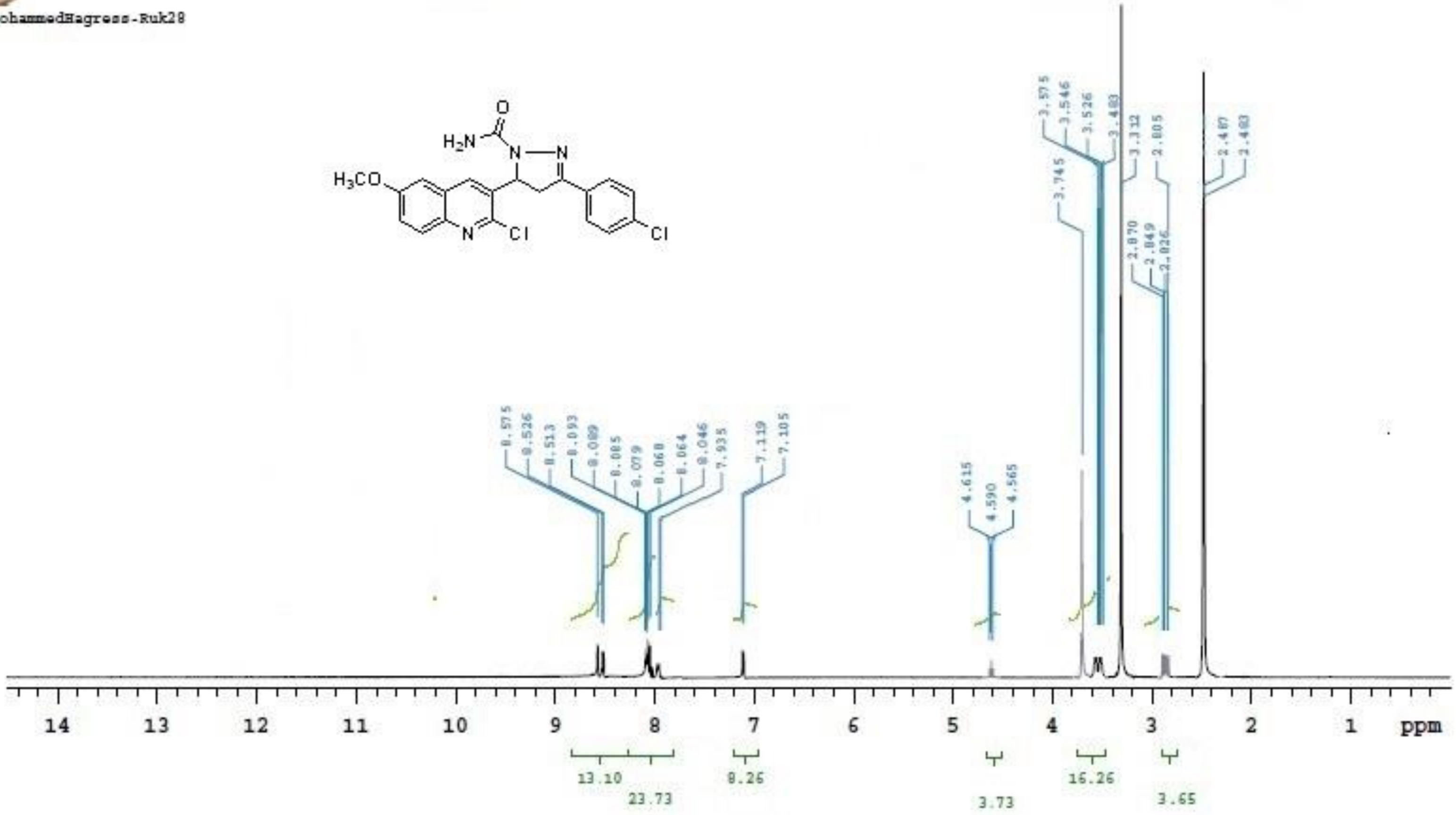

Plotname: Dr_MohammedHagress-Ruk28_PROTON_01_plot02

Data file /home/data/NMRlab2017/Nov/Dr_MohammedHagress-Ruk28_20171112_01/Dr_MohammedHagress-Ruk28_PROTON_01

Plot date 2017-11-12

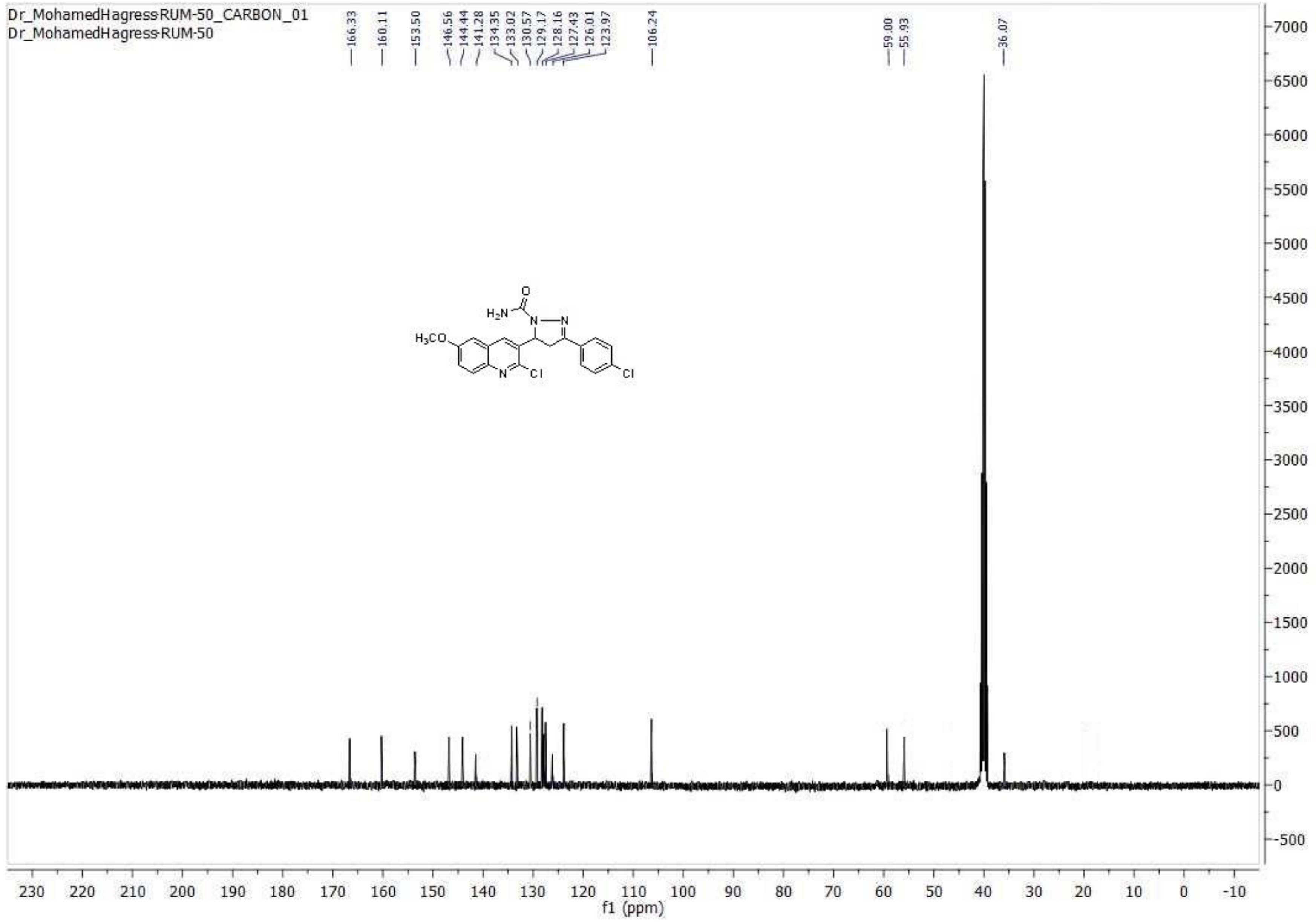

Dr_MohamedHagress-RUM-50_CARBON_01
Dr_MohamedHagress-RUM-50
166.33
160.11
153.50
146.56
144.44
141.28
134.35
133.02
130.57
129.17
128.16
127.43
126.01
123.97
106.24
59.00
55.93
36.07
H3CO
H2N
O
N
Cl
f1 (ppm)

Dr_EmanYahia-ME-1c

Dr_EmanYahia-ME-1c

Sample Name	Dr_EmanYahia-ME-1c	Pulse sequence	PROTON	Temperature	25	Laboratory	MODCL
Date collected	2018-03-19	Solvent	DMSO	Spectrometer	nmr400-mercury400	NMR User	

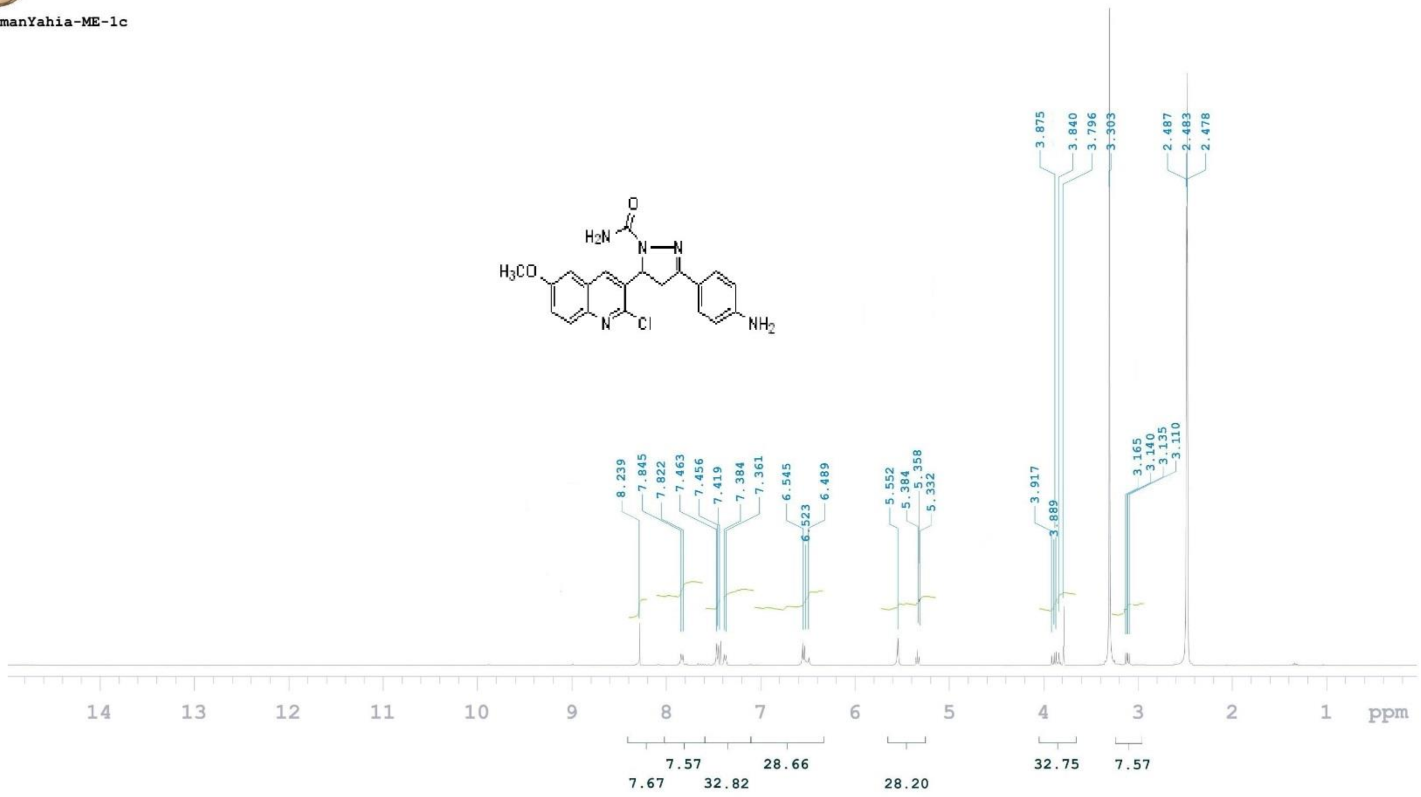

Plotname: Dr_EmanYahia-ME-1c_PROTON_01_plot02

Data file /home/data/NMRlab2018/Mar/Dr_EmanYahia-ME-1c_20180319_01/Dr_EmanYahia-ME-1c_PROTON_01

Plot date 2018-03-19

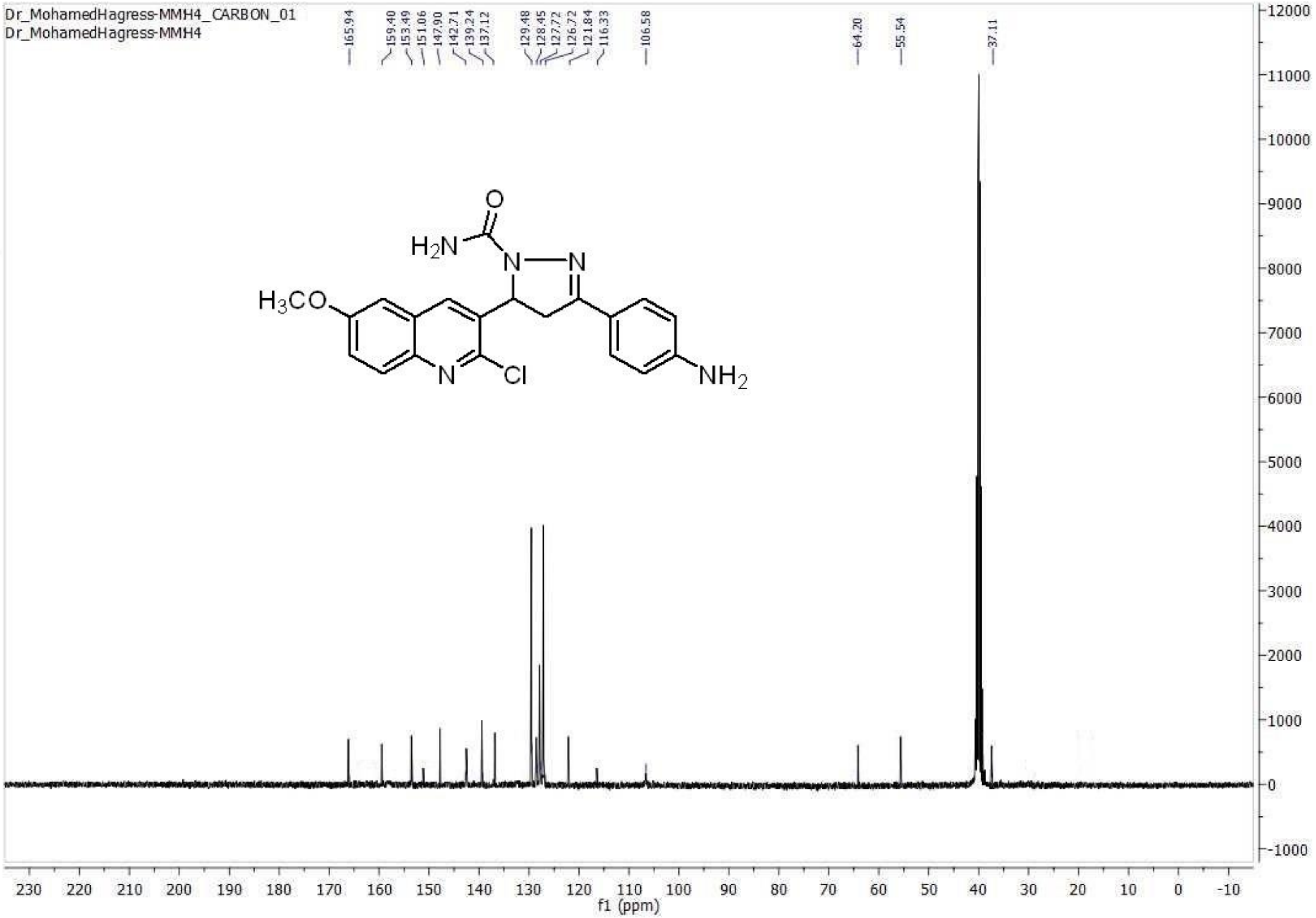

Dr_MohamedHagress-MMH4_CARBON_01
Dr_MohamedHagress-MMH4
165.94
159.40
153.49
151.06
147.90
142.71
139.24
137.12
129.48
128.45
127.72
126.72
121.84
116.33
106.58
64.20
55.54
37.11
O
H2N
N
N
H3CO
N
Cl
NH2
f1 (ppm)

Mohamed Samy-SHK2-DMSO-H1

Archive directory: /export/home/vnmr1/vnmrsys/data
Sample directory: DD5mm_test_12Mar2019-21:34:40
File: PROTON

Pulse Sequence: s2pul

Solvent: DMSO
Temp. 30.0 C / 303.1 K
Mercury-300BB "NMR300"

Relax. delay 1.000 sec
Pulse 45.0 degrees
Acq. time 4.853 sec
Width 6600.7 Hz
8 repetitions
OBSERVE H1, 300.0687865 MHz
DATA PROCESSING
FT size 65536
Total time 5 min, 16 sec
Date: Feb 5 2019

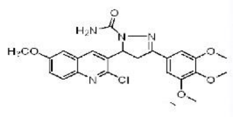

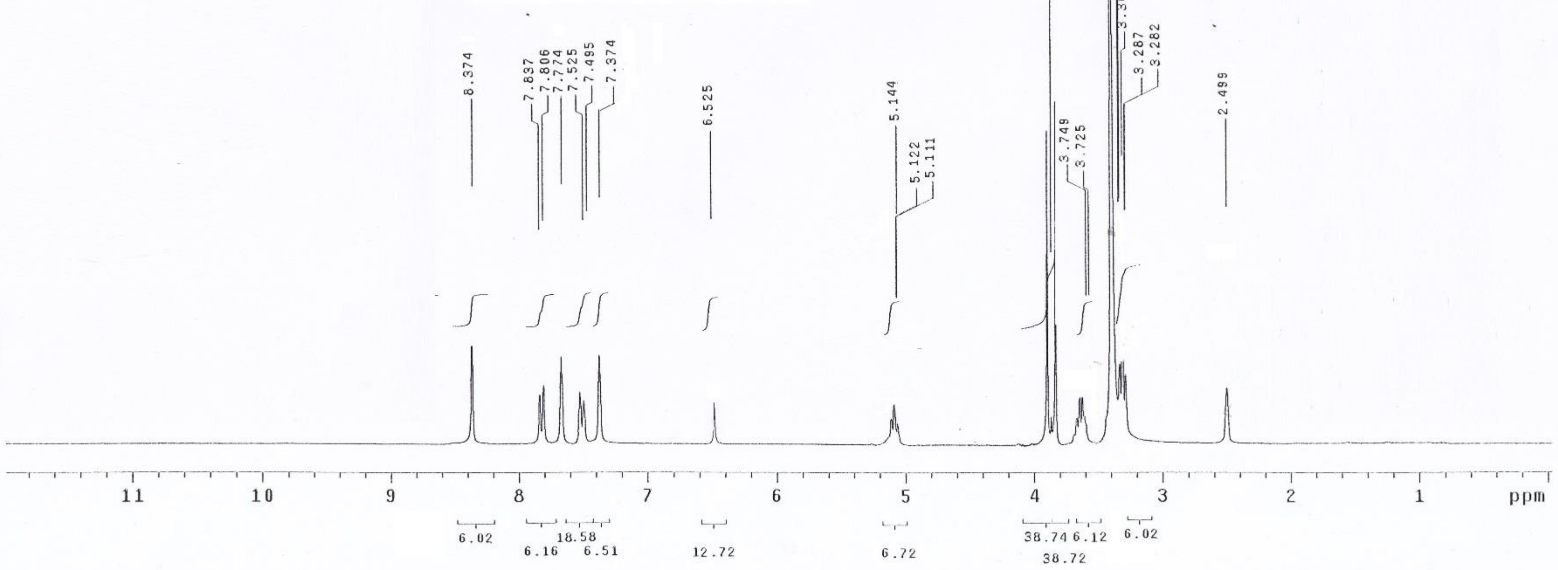

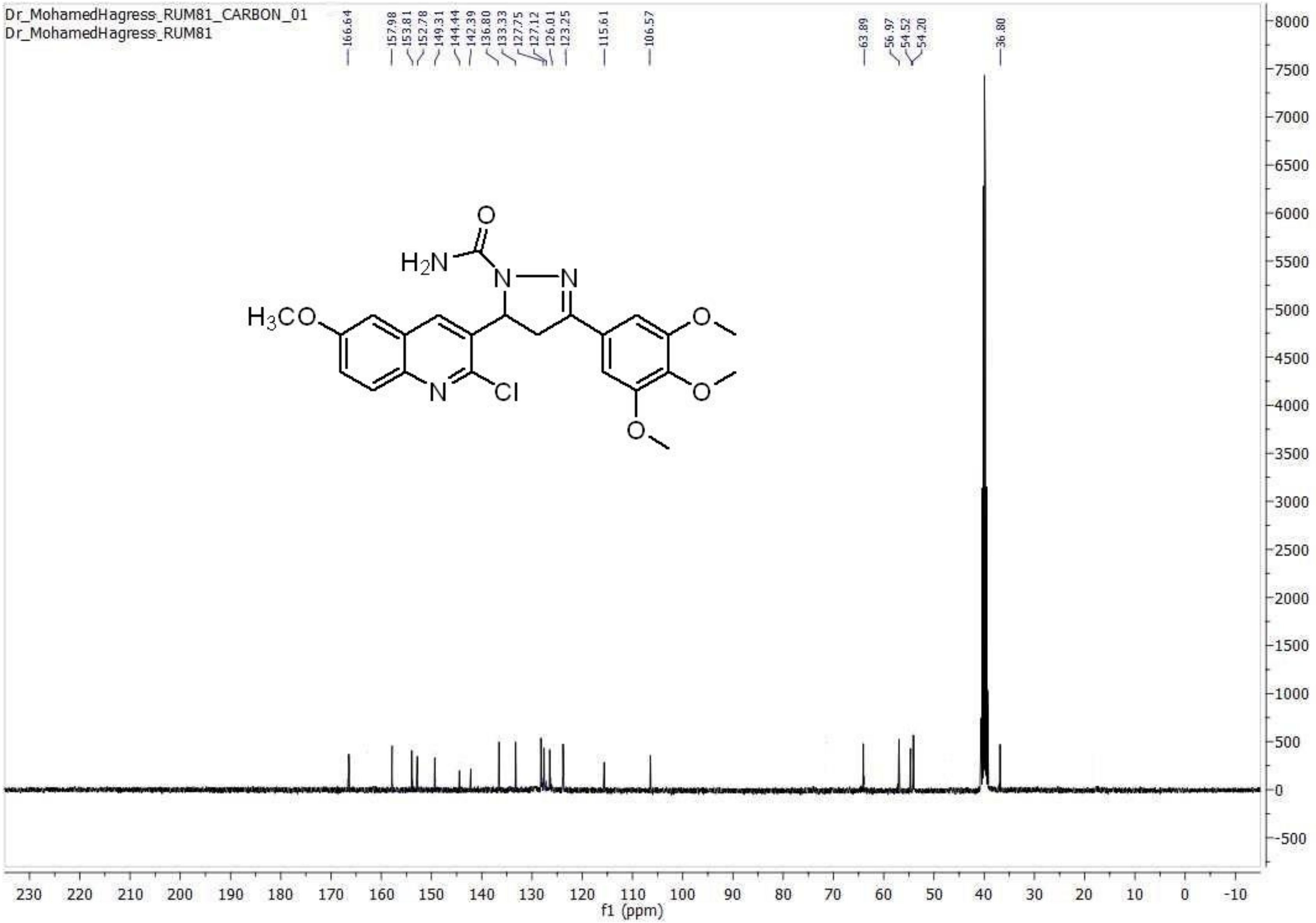

Dr_MohamedHagress_RUM81_CARBON_01
Dr_MohamedHagress_RUM81
166.64
157.98
153.81
152.78
149.31
144.44
142.39
136.80
133.33
127.75
127.12
126.01
123.25
115.61
106.57
63.89
56.97
54.52
54.20
36.80
H2N
O
N
N
H3CO
N
Cl
O
O
O
f1 (ppm)

Dr_MohammedHagress-Ruk27

Dr_MohammedHagress-Ruk27

Sample Name	Dr_MohammedHagress-Ruk27	Pulse sequence	PROTON	Temperature	26	Study owner	vnmr1
Date collected	2017-11-12	Solvent	dmso	Spectrometer	nmr400-mercury400	Operator	vnmr1

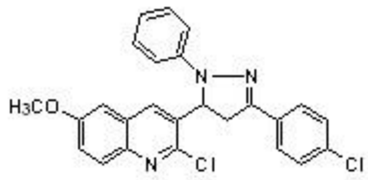

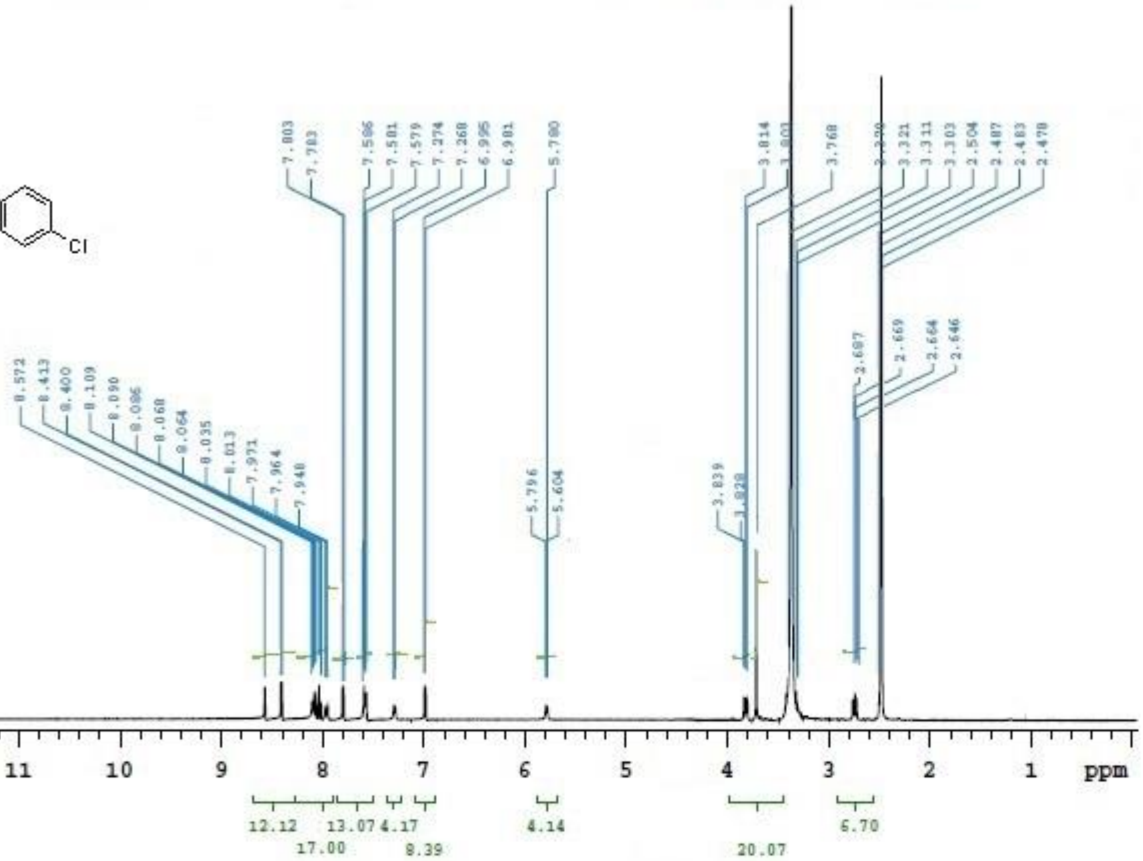

Plotname: Dr_MohammedHagress-Ruk27_PROTON_01_plot02

Data file /home/data/NMRlab2017/Nov/Dr_MohammedHagress-Ruk27_20171112_01/Dr_MohammedHagress-Ruk27_PROTON_01

Plot date 2017-11-12

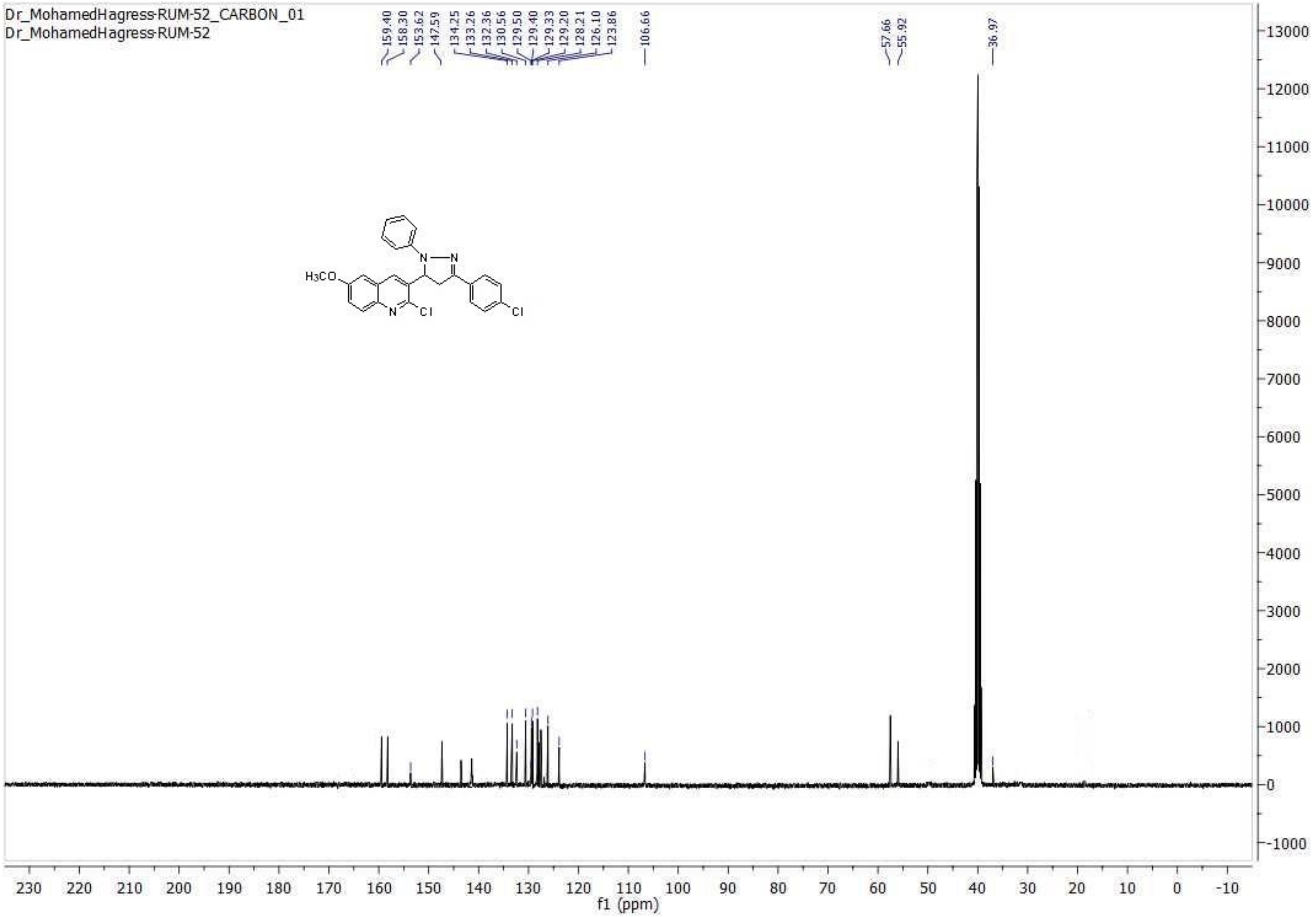
Dr_MohamedHagress-RUM-52_CARBON_01
Dr_MohamedHagress-RUM-52
159.40
158.30
153.62
147.59
134.25
133.26
132.36
130.56
129.50
129.40
129.33
129.20
128.21
126.10
123.86
106.66
57.66
55.92
36.97
H3CO
N
Cl
f1 (ppm)

Dr_EmanYahia-ME-1f

Dr_EmanYahia-ME-1f

Sample Name	Dr_EmanYahia-ME-1f	Pulse sequence	PROTON	Temperature	25	Laboratory	MODCL
Date collected	2018-03-19	Solvent	DMSO	Spectrometer	nmr400-mercury400	NMR User	sameeh_Albadawy

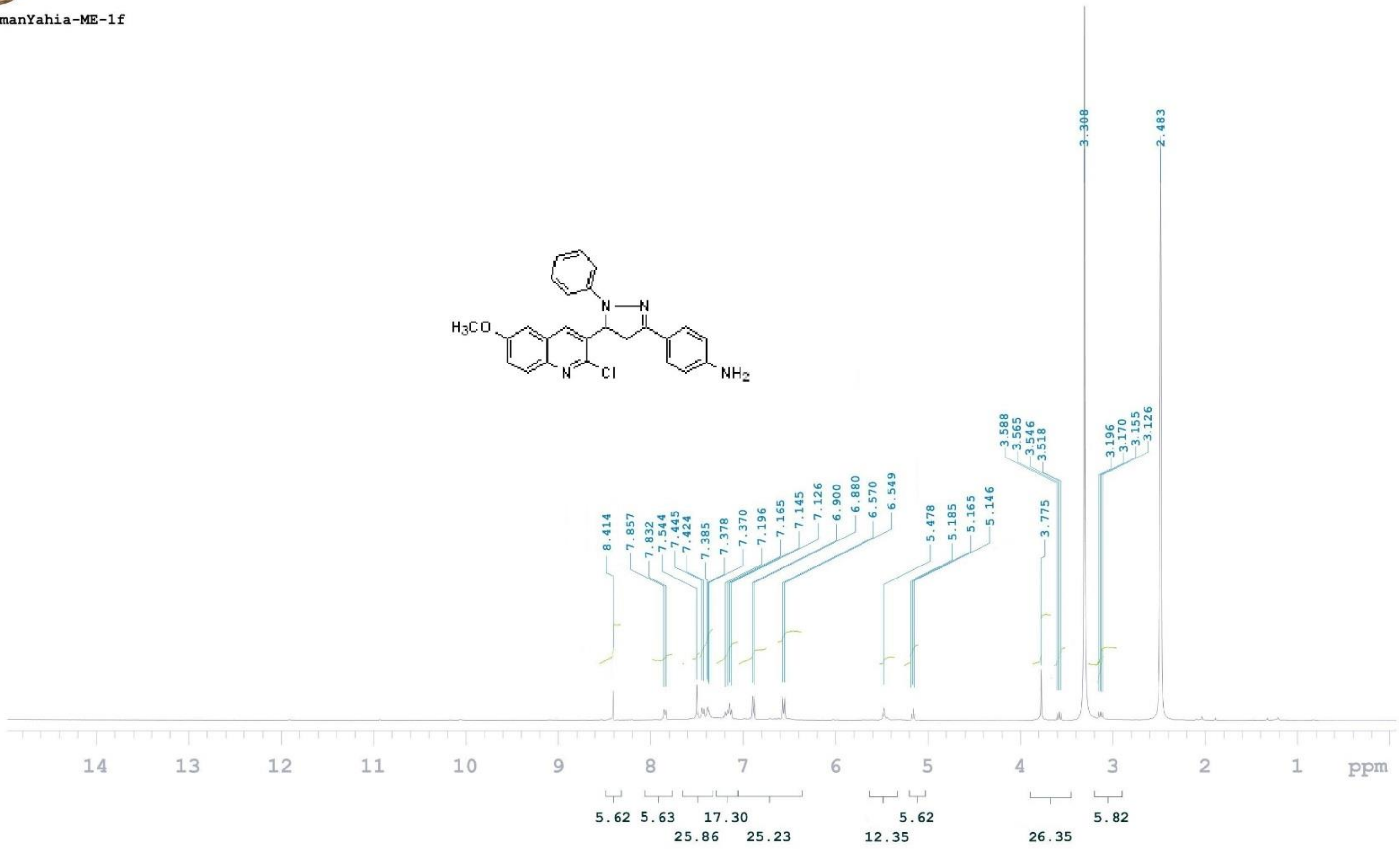

Plotname: Dr_EmanYahia-ME-1f_PROTON_01_plot02

Data file /home/data/NMRlab2018/Mar/Dr_EmanYahia-ME-1f_20180319_01/Dr_EmanYahia-ME-1f_PROTON_01

Plot date 2018-03-19

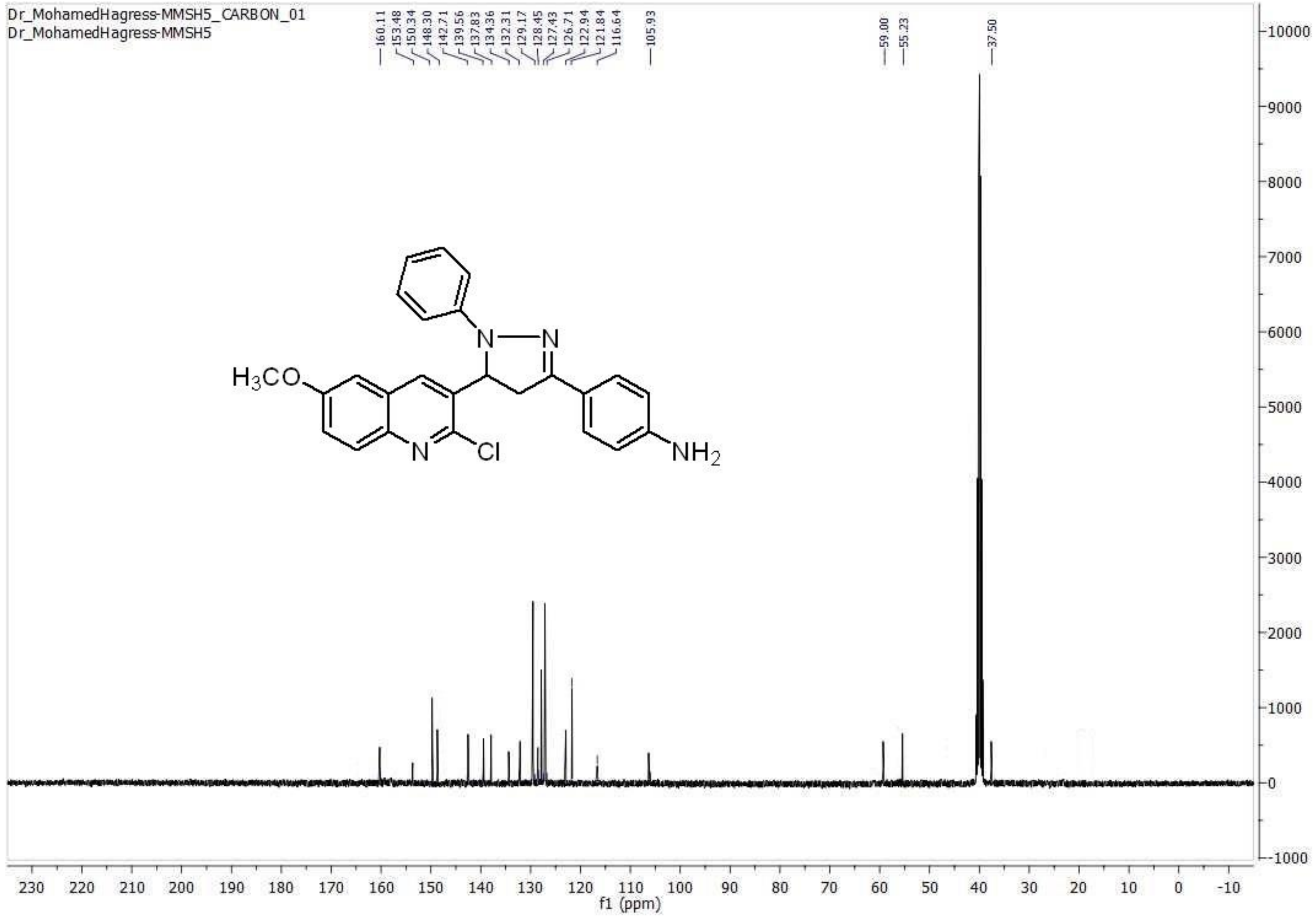

Dr_MohamedHagress-MMSH5_CARBON_01
Dr_MohamedHagress-MMSH5
160.11
153.48
150.34
148.30
142.71
139.56
137.83
134.36
132.31
129.17
128.45
127.43
126.71
122.94
121.84
116.64
105.93
59.00
55.23
37.50
H3CO
N
N
N
Cl
NH2
f1 (ppm)

H2-proton

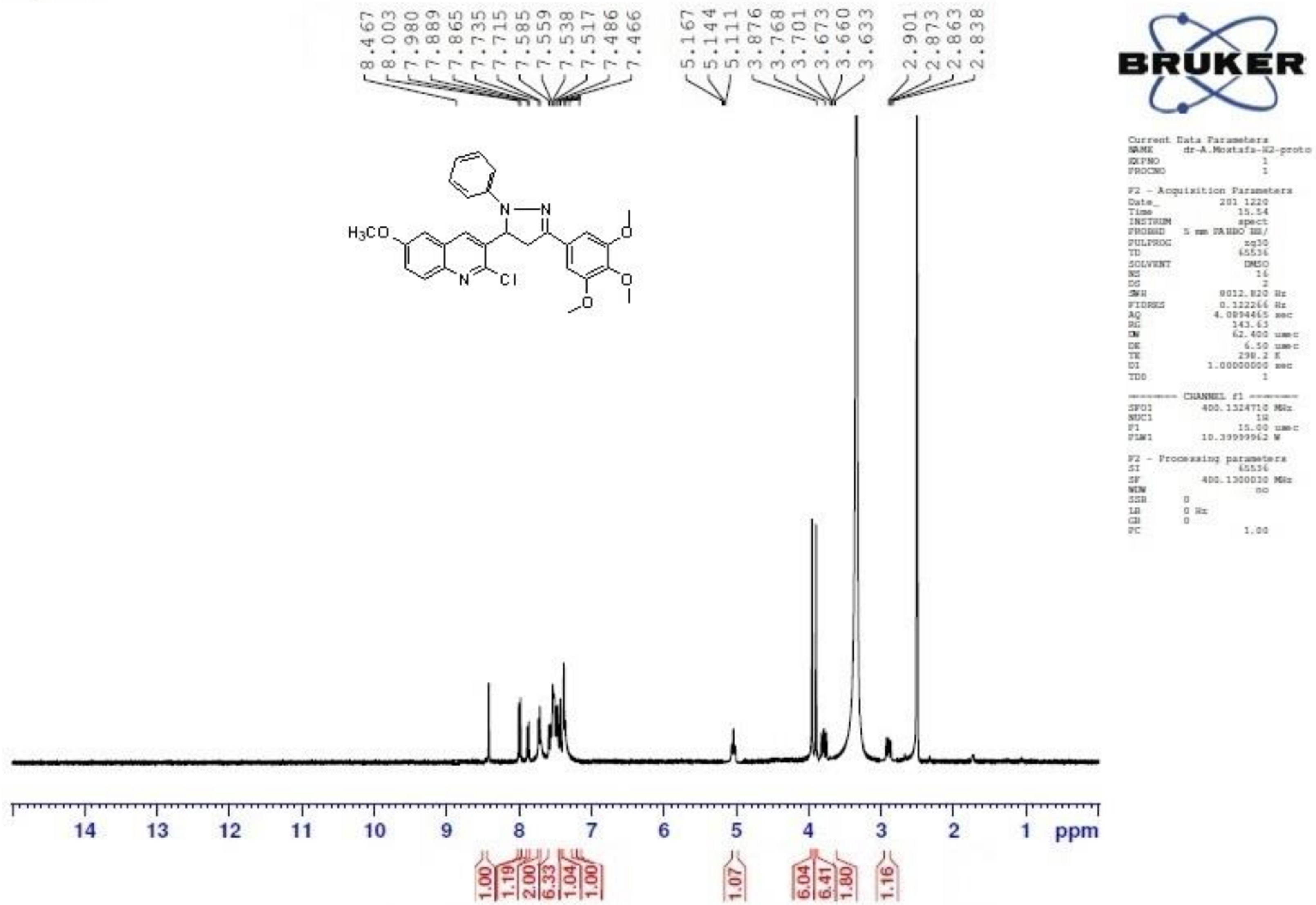

8.467
8.003
7.980
7.889
7.865
7.735
7.715
7.585
7.559
7.538
7.517
7.486
7.466
5.167
5.144
5.111
3.876
3.768
3.701
3.673
3.660
3.633
2.901
2.873
2.863
2.838
H3CO
N
N
N
Cl
O
O
O
14
13
12
11
10
9
8
7
6
5
4
3
2
1
ppm
1.00
1.19
2.00
6.33
1.04
1.00
1.07
6.04
6.41
1.80
1.16
BRUKER
Current Data Parameters
NAME dr-A.Mostafa-H2-proto
EXPNO 1
PROCNO 1
F2 - Acquisition Parameters
Date_ 201 1220
Time 15.54
INSTRUM spect
PROBHD 5 mm PABBO BB/
PULPROG zg30
TD 65536
SOLVENT DMSO
NS 16
DS 2
SWH 8012.820 Hz
FIDRES 0.122266 Hz
AQ 4.0894465 sec
RG 143.63
DW 62.400 usec
DE 6.50 usec
TE 298.2 K
D1 1.00000000 sec
TD0 1
======== CHANNEL f1 ========
SFO1 400.1324710 MHz
NUC1 1H
P1 15.00 usec
PLW1 10.39999962 W
F2 - Processing parameters
SI 65536
SF 400.1300030 MHz
WDW no
SSB 0
LB 0 Hz
GB 0
PC 1.00

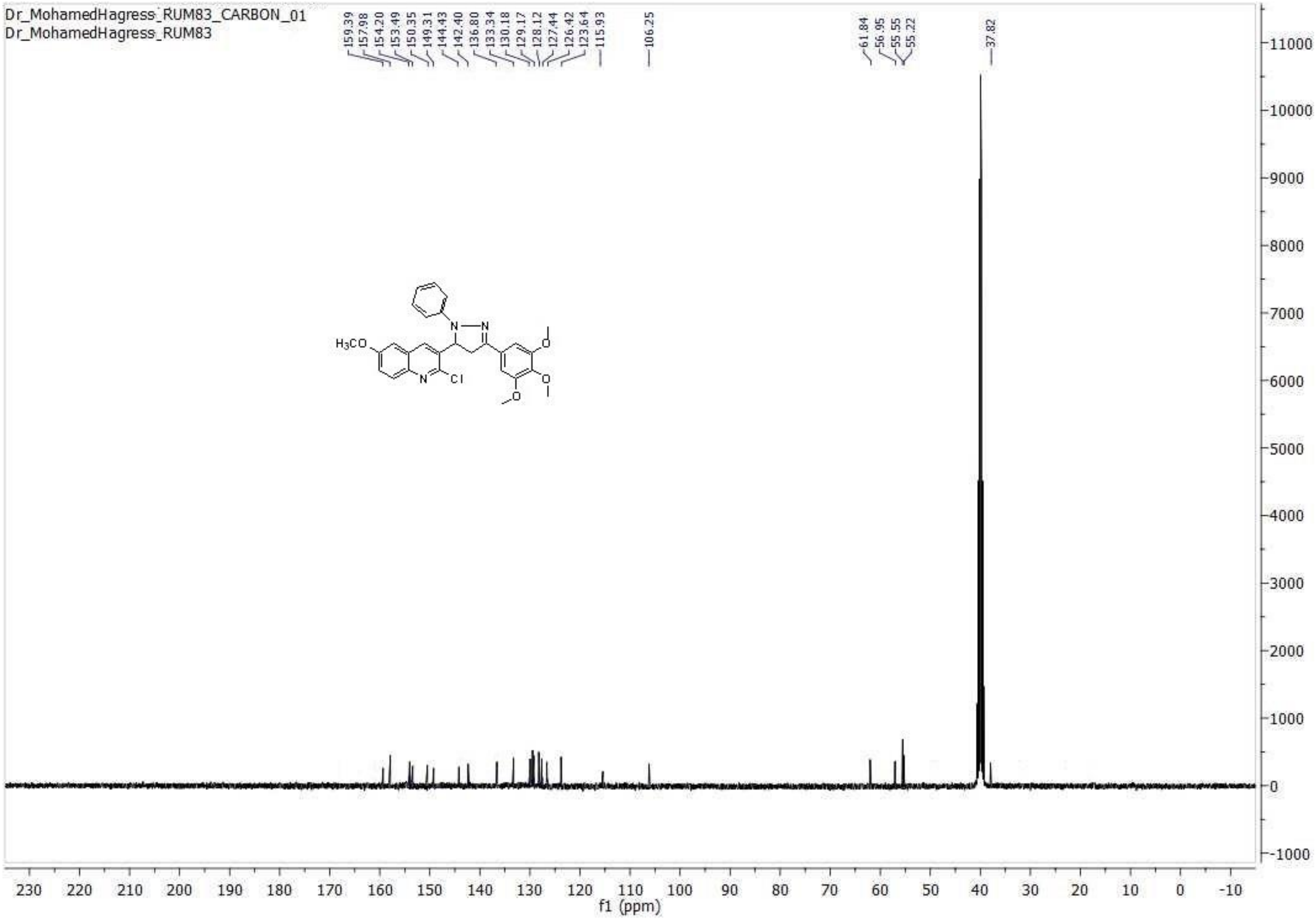

Dr_MohamedHagress_RUM83_CARBON_01
Dr_MohamedHagress_RUM83
159.39
157.98
154.20
153.49
150.35
149.31
144.43
142.40
136.80
133.34
130.18
129.17
128.12
127.44
126.42
123.64
115.93
106.25
61.84
56.95
55.55
55.22
37.82
H3CO
N
N
N
Cl
O
O
O
f1 (ppm)

Dr_MohammedHagress-Ruk3

Dr_MohammedHagress-Ruk3

Sample Name	Dr_MohammedHagress-Ruk3	Pulse sequence	PROTON	Temperature	26	Study owner	vnmr1
Date collected	2017-11-06	Solvent	dmso	Spectrometer	nmr400-mercury400	Operator	vnmr1

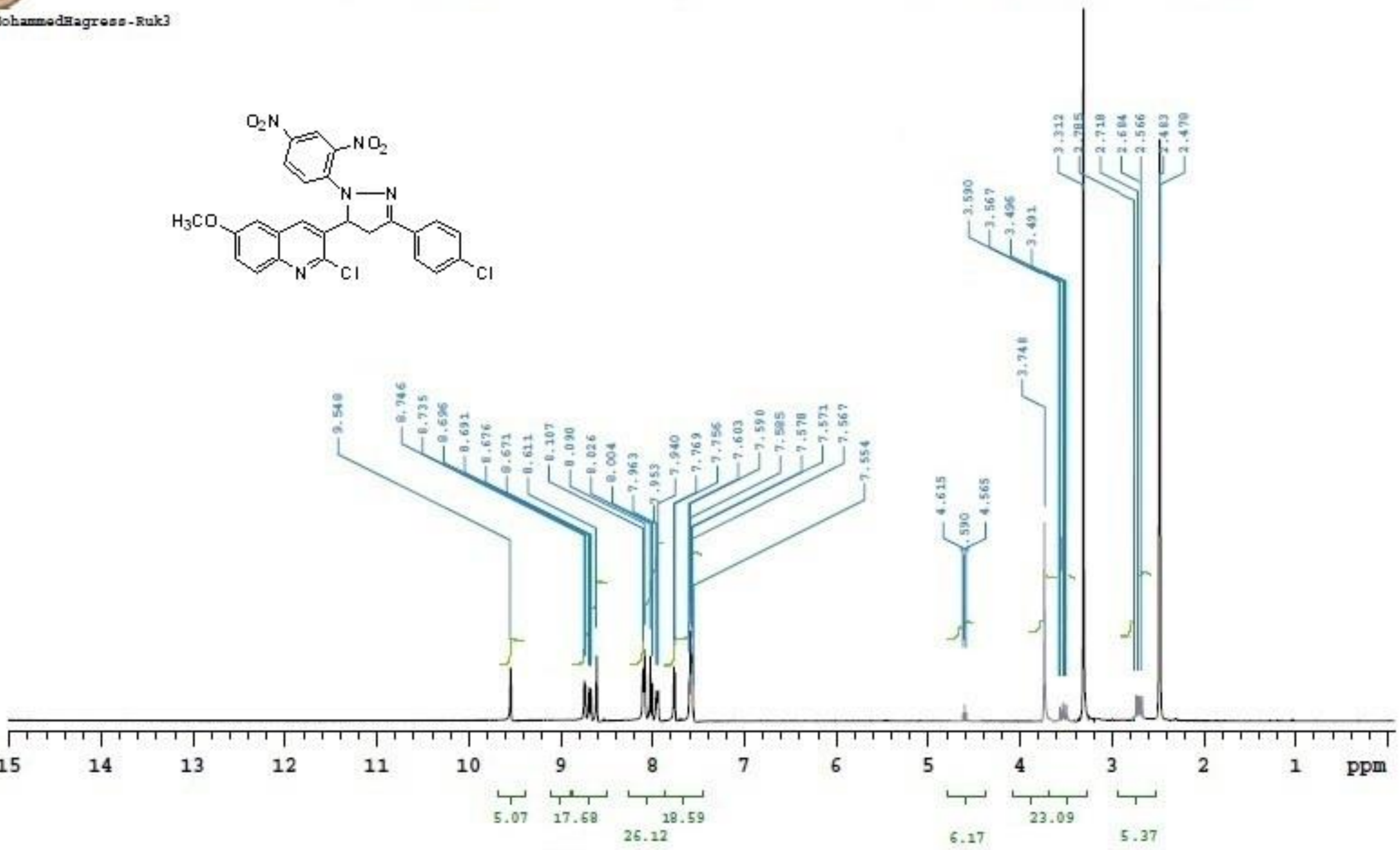

Plotname: Dr_MohammedHagress-Ruk3_PROTON_01_plot02

Data file /home/data/NMRlab2017/Nov/Dr_MohammedHagress-Ruk3_20171106_01/Dr_MohammedHagress-Ruk3_PROTON_01

Plot date 2017-11-06

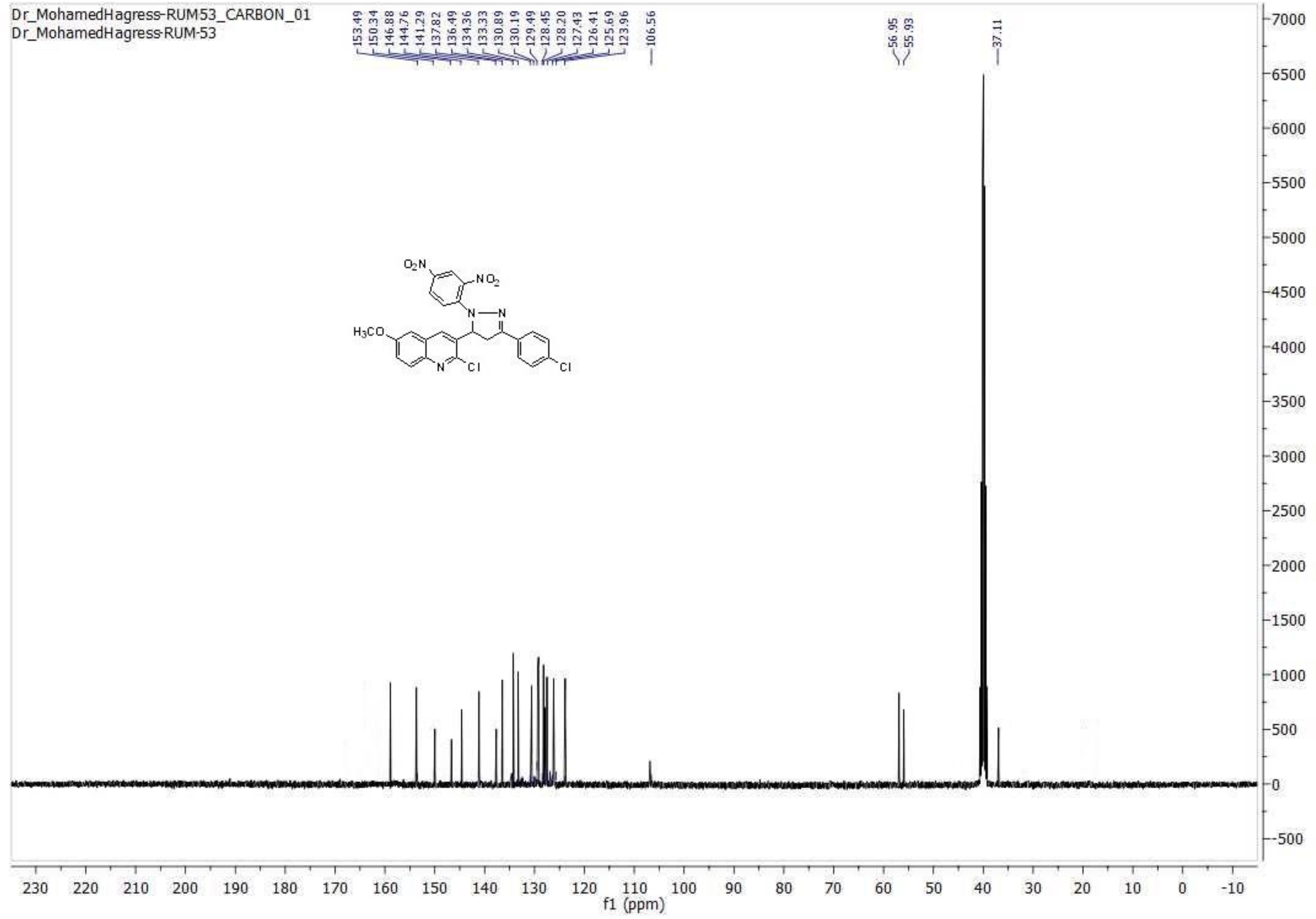

Dr_MohamedHagress-RUM53_CARBON_01
Dr_MohamedHagress-RUM-53
153.49
150.34
146.88
144.76
141.29
137.82
136.49
134.36
133.33
130.89
130.19
129.49
128.45
128.20
127.43
126.41
125.69
123.96
106.56
56.95
55.93
37.11
O2N
NO2
N
N
H3CO
N
Cl
Cl
f1 (ppm)

Dr_EmanYahia-ME9

Sample Name	**Dr_EmanYahia-ME9**	Pulse sequence	**PROTON**	Temperature	**25**	Laboratory	**MODCL**
Date collected	**2018-07-18**	Solvent	**DMSO**	Spectrometer	**nmr400-mercury400**	NMR User	**sameeh_Albadawy**

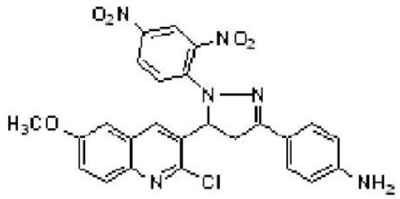

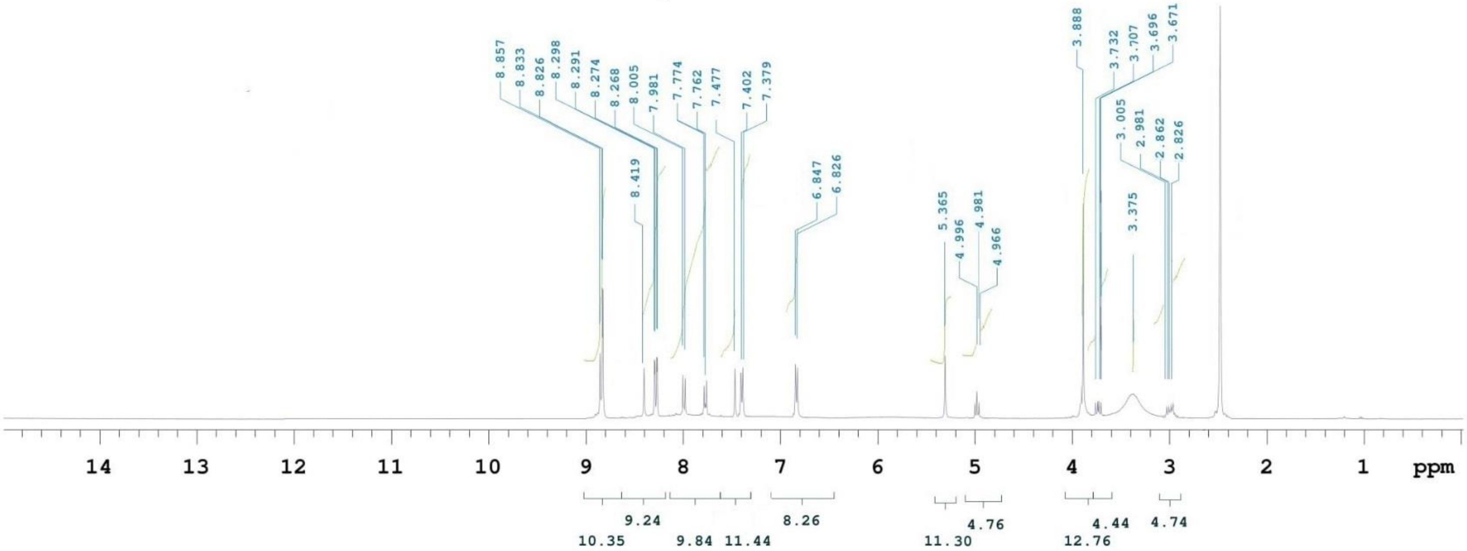

Plotname: Dr_EmanYahia-ME9_PROTON_01_plot01

Data file /home/data/NMRlab2018/Jul/Dr_EmanYahia-ME9_20180718_01/Dr_EmanYahia-ME9_PROTON_01

Plot date 2018-07-18

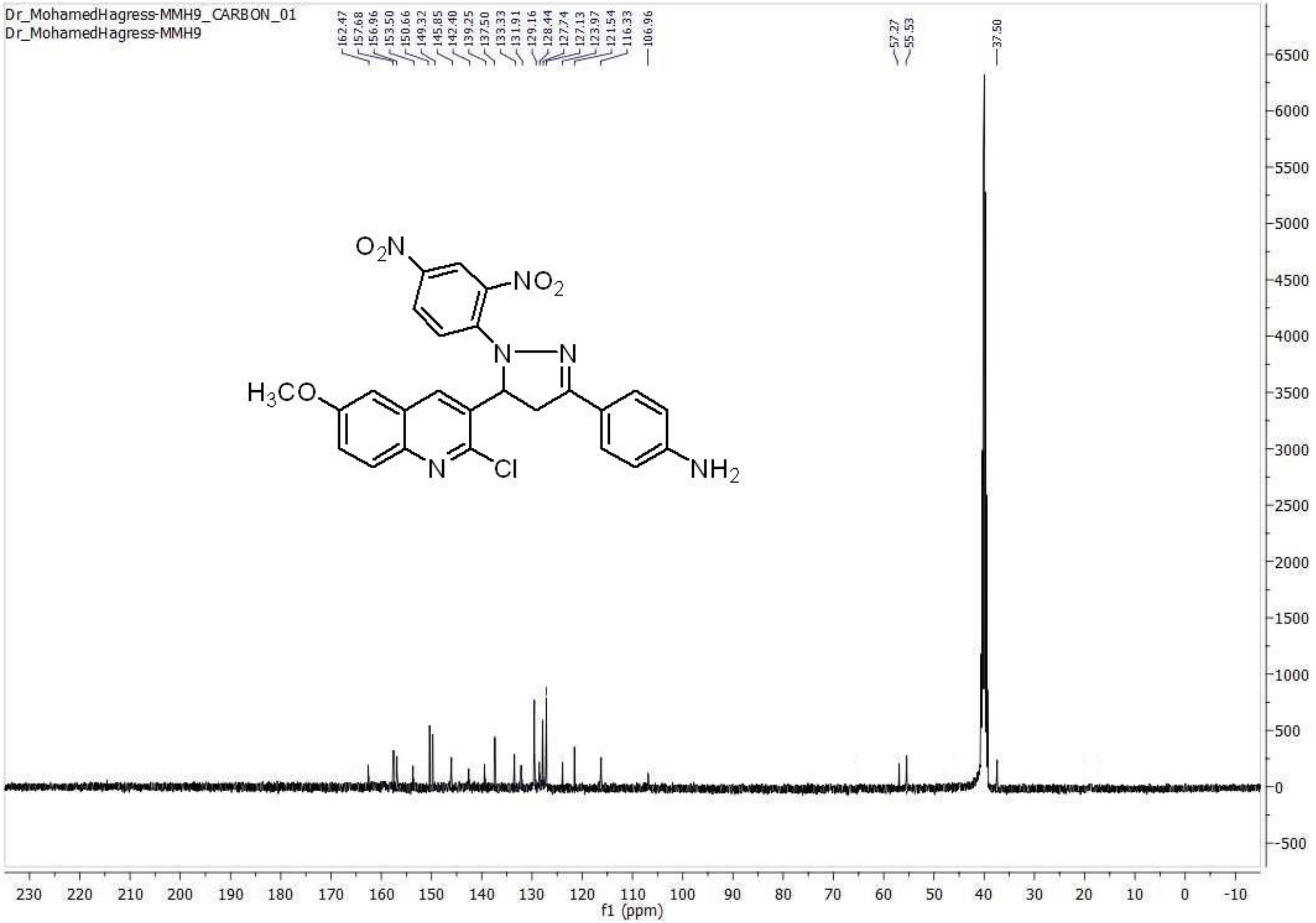
Dr_MohamedHagress-MMH9_CARBON_01
Dr_MohamedHagress-MMH9
162.47
157.68
156.96
153.50
150.66
149.32
145.85
142.40
139.25
137.50
133.33
131.91
129.16
128.44
127.74
127.13
123.97
121.54
116.33
106.96
57.27
55.53
37.50
O2N
NO2
N
N
H3CO
N
Cl
NH2
f1 (ppm)

Mohamed Samy-SHE1-DMSO-H1

Archive directory: /export/home/vnmr1/vnmrsys/data
Sample directory: DD5mm_test_12Mar2019-21:34:40
File: PROTON

Pulse Sequence: s2pul

Solvent: DMSO
Temp. 30.0 C / 303.1 K
Mercury-300BB "NMR300"

Relax. delay 1.000 sec
Pulse 45.0 degrees
Acq. time 4.853 sec
Width 6600.7 Hz
13 repetitions
OBSERVE H1, 300.0687865 MHz
DATA PROCESSING
FT size 65536
Total time 5 min, 16 sec
Date: Feb 5 2019

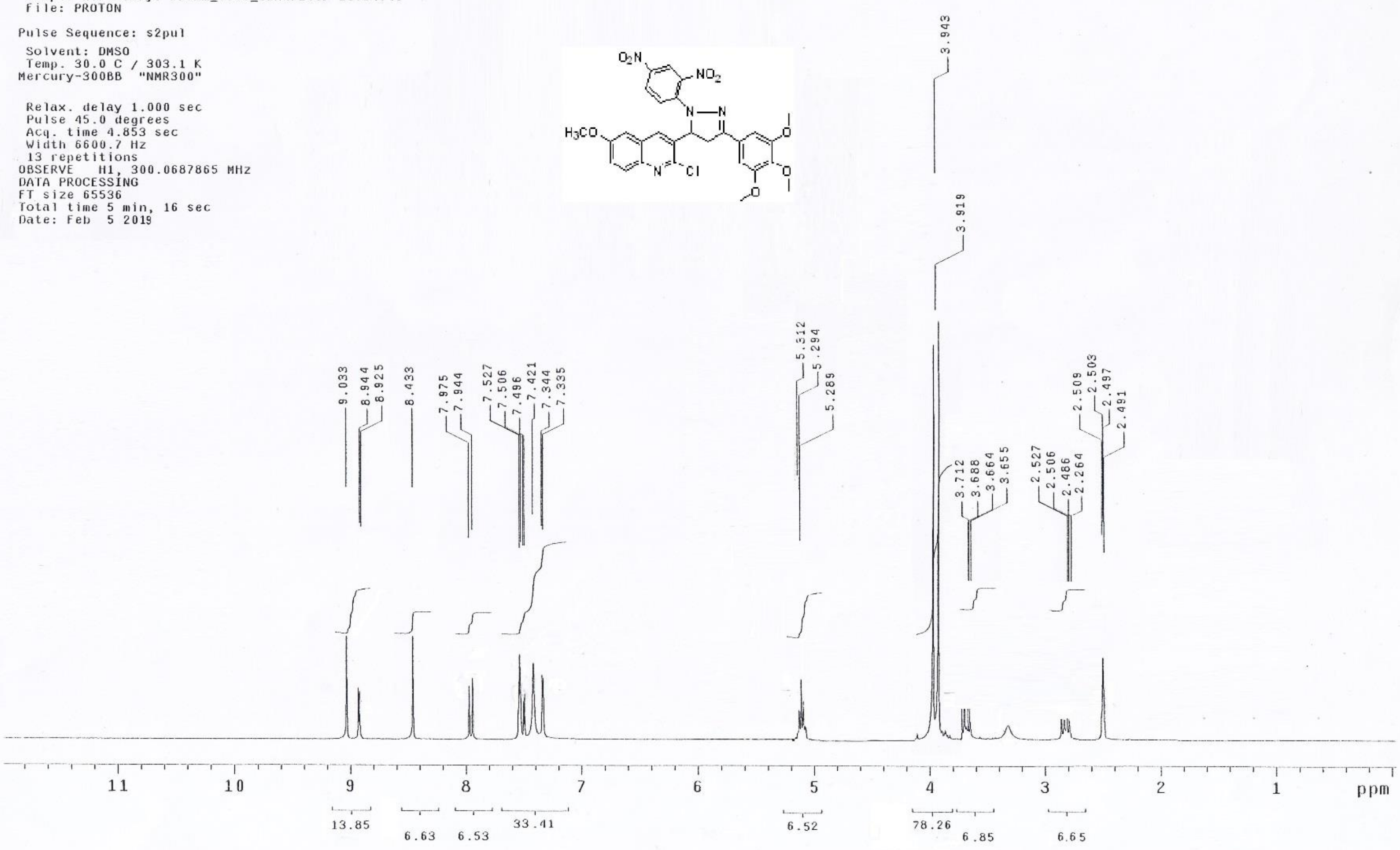

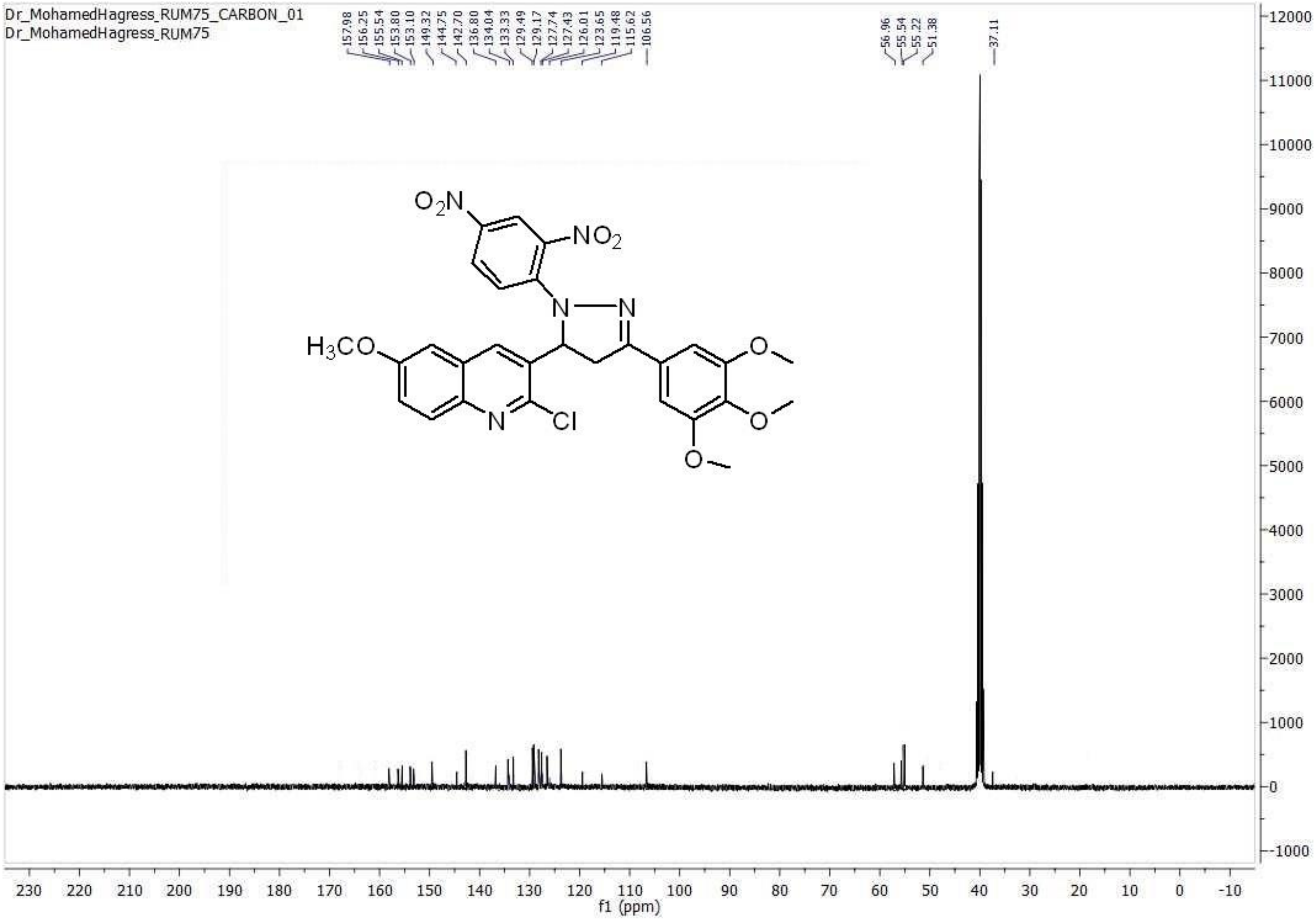

Dr_MohamedHagress_RUM75_CARBON_01
Dr_MohamedHagress_RUM75
157.98
156.25
155.54
153.80
153.10
149.32
144.75
142.70
136.80
134.04
133.33
129.49
129.17
127.74
127.43
126.01
123.65
119.48
115.62
106.56
56.96
55.54
55.22
51.38
37.11
O_2N
NO_2
N
N
H_3CO
N
Cl
O
O
O
f1 (ppm)

Dr_EmanYahia-SH

Sample Name **Dr_EmanYahia-SH**	Pulse sequence **PROTON**	Temperature **26**	Laboratory **MODCL**
Date collected **2018-07-18**	Solvent **DMSO**	Spectrometer **nmr400-mercury400**	NMR User **sameeh_Albadawy**

Dr_EmanYahia-SH

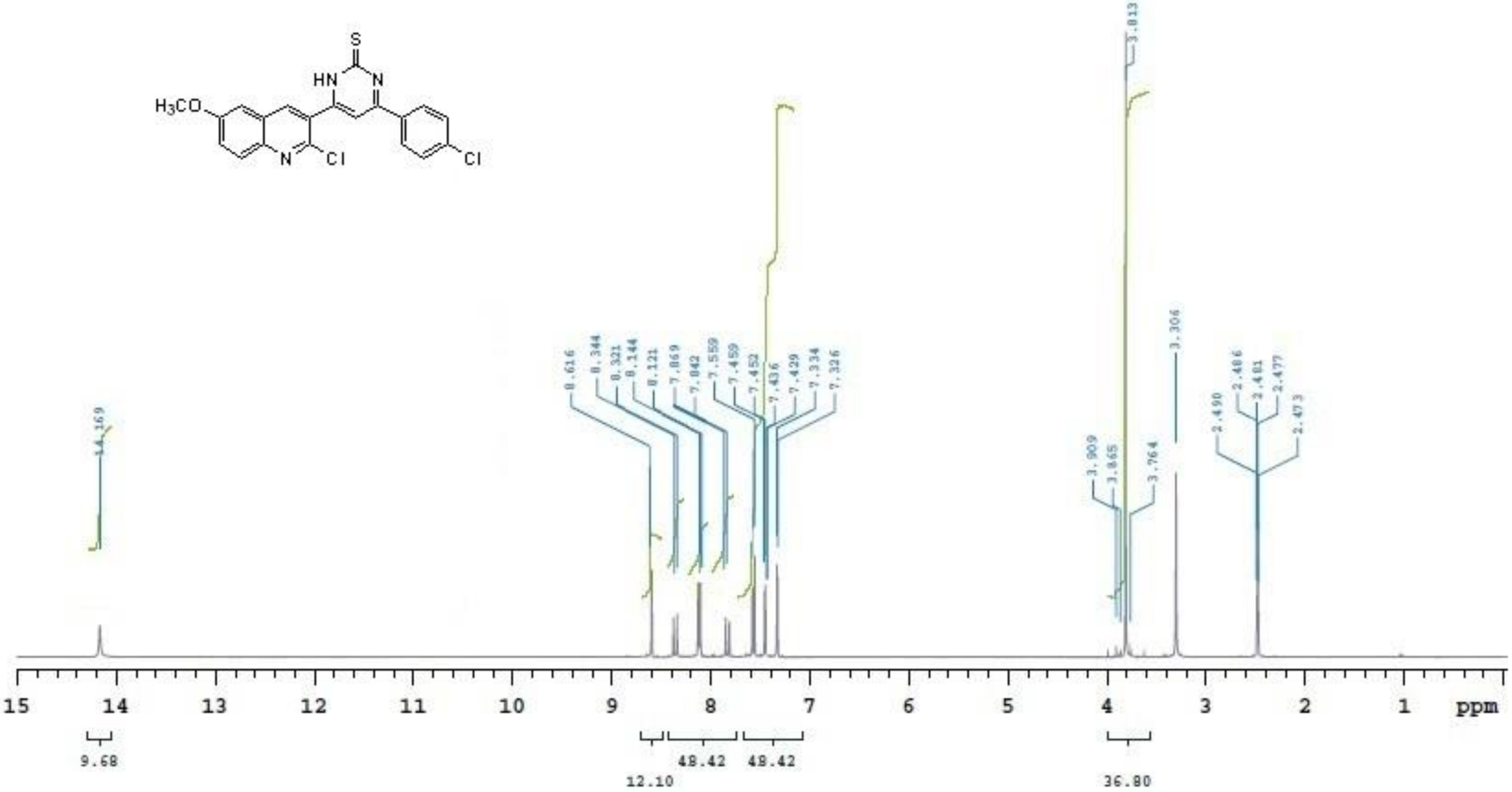

Plotname: Dr_EmanYahia-SH_PROTON_01_plot02

Data file /home/data/NMRlab2018/Jul/Dr_EmanYahia-SH_20180718_01/Dr_EmanYahia-SH_PROTON_01

Plot date 2018-07-18

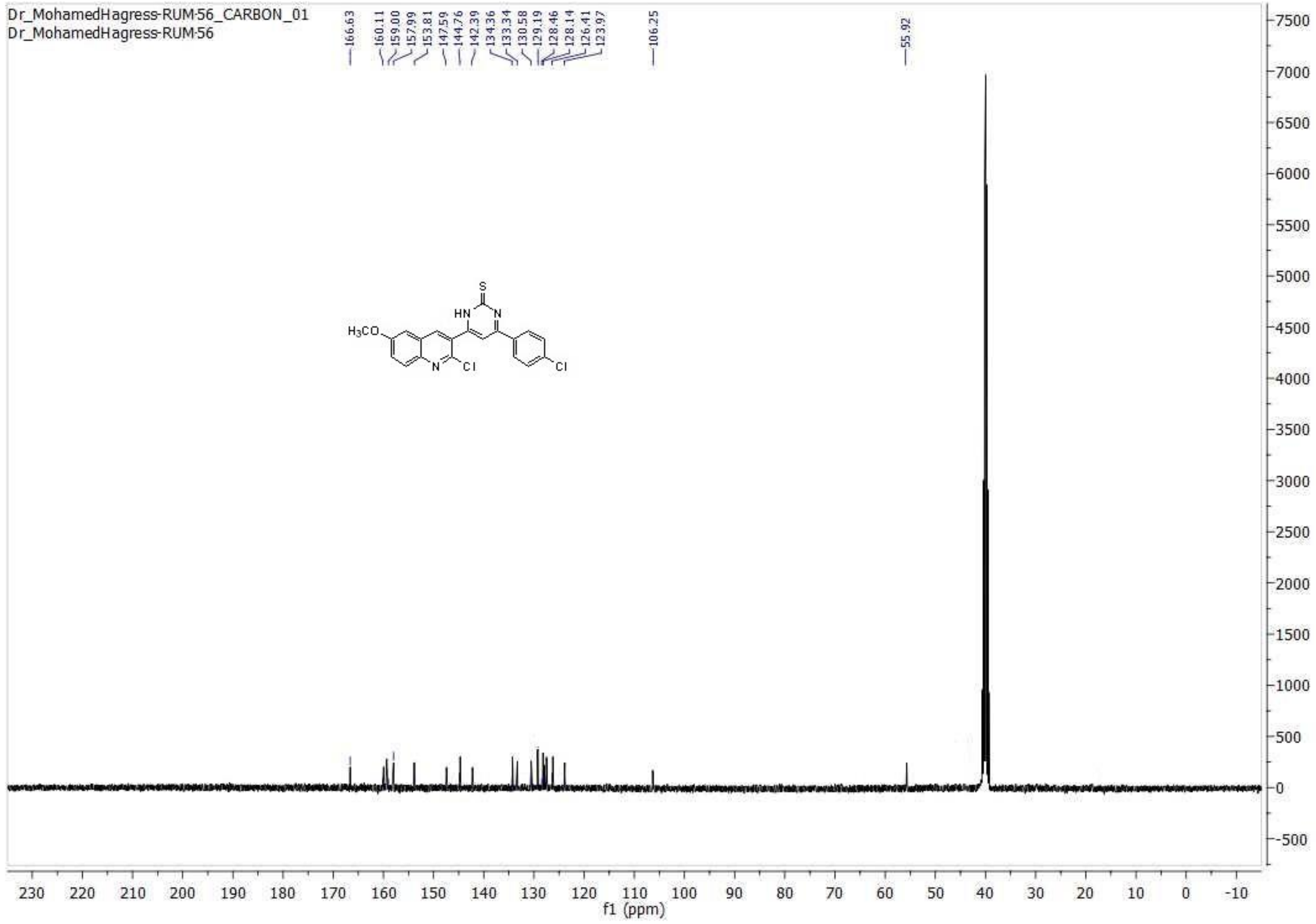

Dr_MohamedHagress-RUM-56_CARBON_01
Dr_MohamedHagress-RUM-56
166.63
160.11
159.00
157.99
153.81
147.59
144.76
142.39
134.36
133.34
130.58
129.19
128.46
128.14
126.41
123.97
106.25
55.92
f1 (ppm)

Dr_EmanYahia-ME-1d

Dr_EmanYahia-ME-1d

Sample Name	Dr_EmanYahia-ME-1d	Pulse sequence	PROTON	Temperature	25	Laboratory	MODCL
Date collected	2018-03-19	Solvent	DMSO	Spectrometer	nmr400-mercury400	NMR User	sameeh_Albadawy

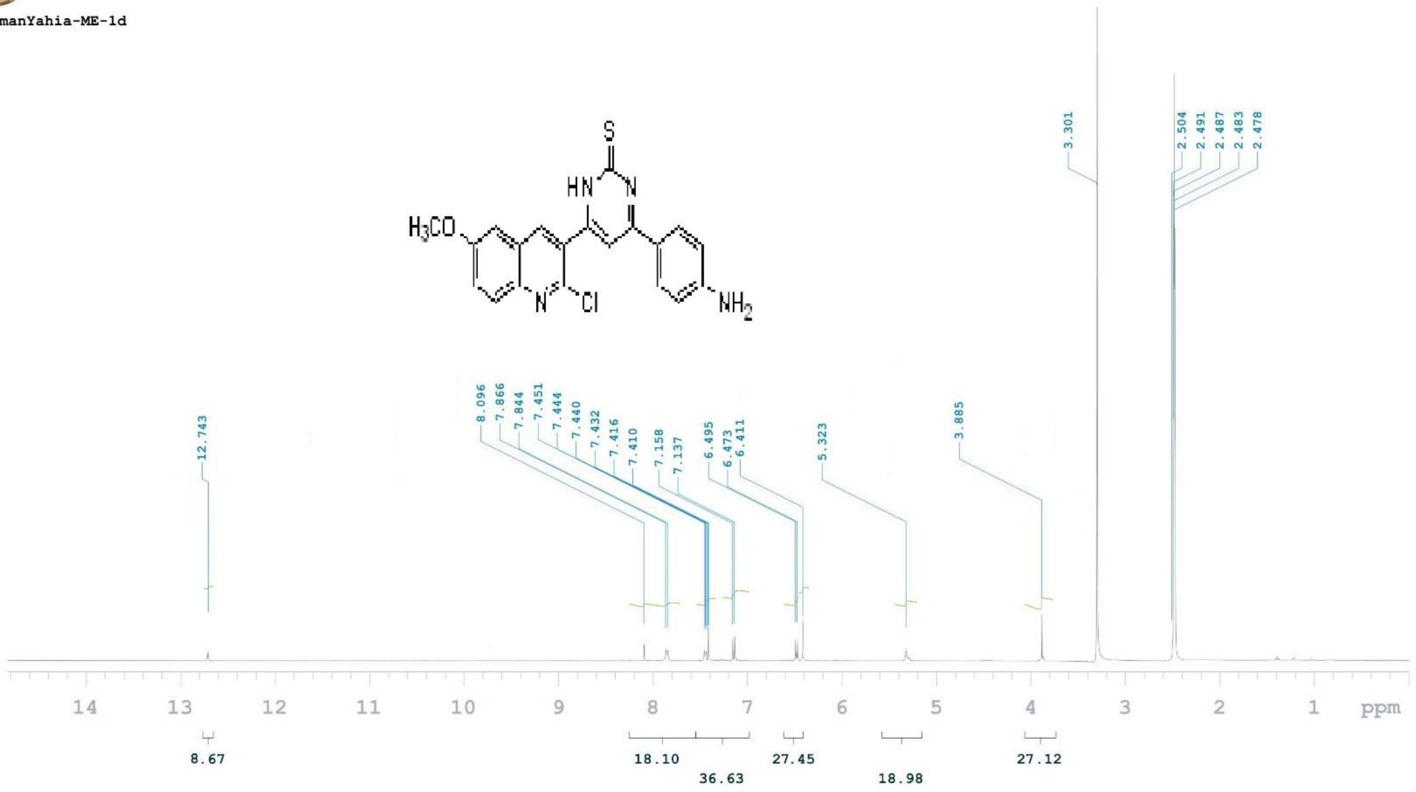

Plotname: Dr_EmanYahia-ME-1d_PROTON_01_plot03

Data file /home/data/NMRlab2018/Mar/Dr_EmanYahia-ME-1d_20180319_01/Dr_EmanYahia-ME-1d_PROTON_01

Plot date 2018-03-19

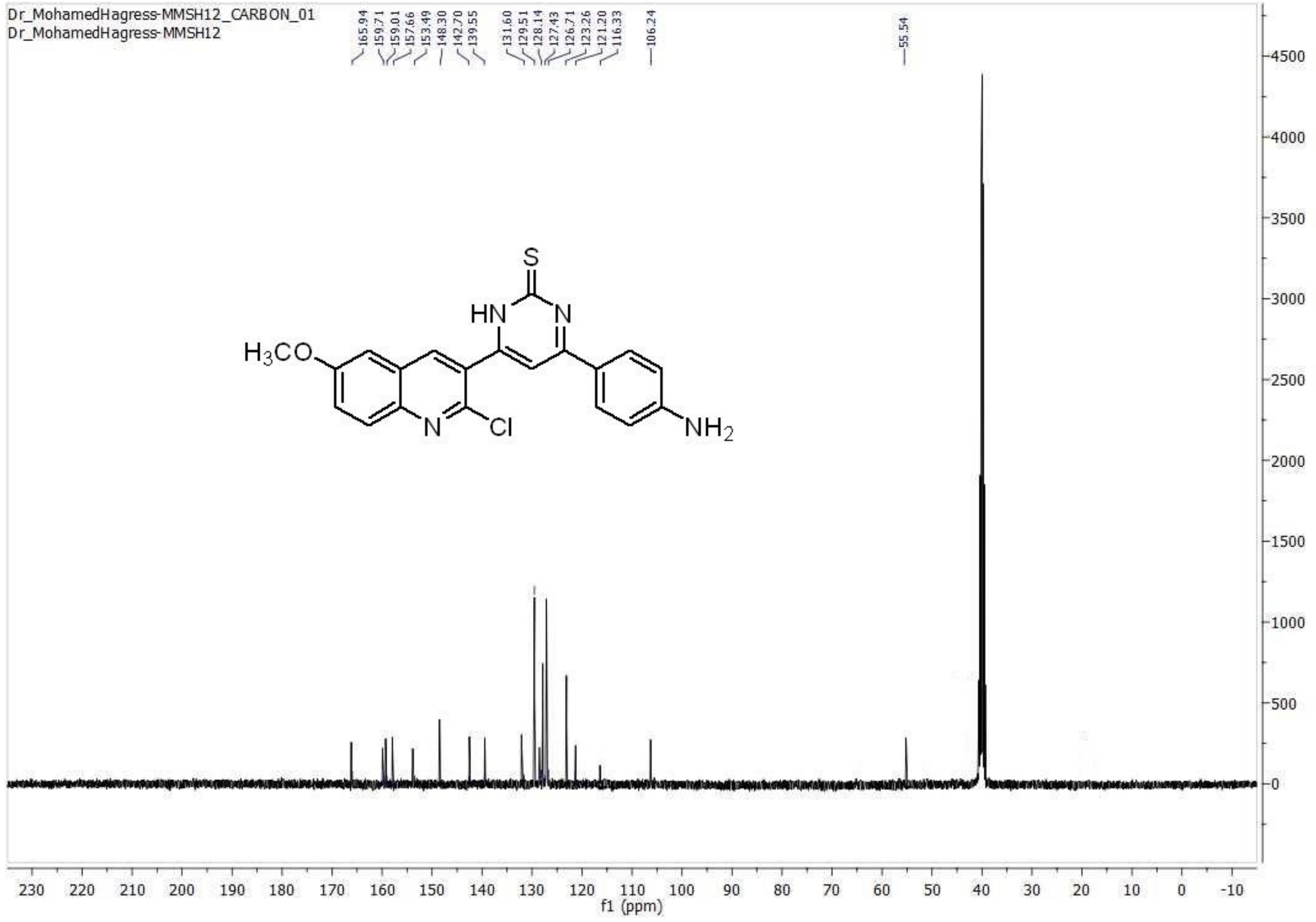

Dr_MohamedHagress-MMSH12_CARBON_01
Dr_MohamedHagress-MMSH12
165.94
159.71
159.01
157.66
153.49
148.30
142.70
139.55
131.60
129.51
128.14
127.43
126.71
123.26
121.20
116.33
106.24
55.54
S
HN
N
H_3CO
N
Cl
NH_2
f1 (ppm)

Mohamed Samy-SHK8-DMSO-H1

Archive directory: /export/home/vnmr1/vnmrsys/data
Sample directory: DD5mm_test_12Mar2019-21:34:40
File: PROTON

Pulse Sequence: s2pul

Solvent: DMSO
Temp. 30.0 C / 303.1 K
Mercury-300BB "NMR300"

Relax. delay 1.000 sec
Pulse 45.0 degrees
Acq. time 4.853 sec
Width 6600.7 Hz
8 repetitions
OBSERVE H1, 300.0687865 MHz
DATA PROCESSING
FT size 65536
Total time 5 min, 16 sec
Date: Feb 5 2019

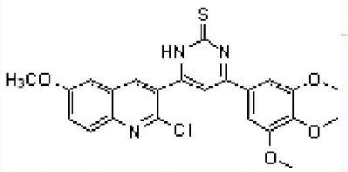

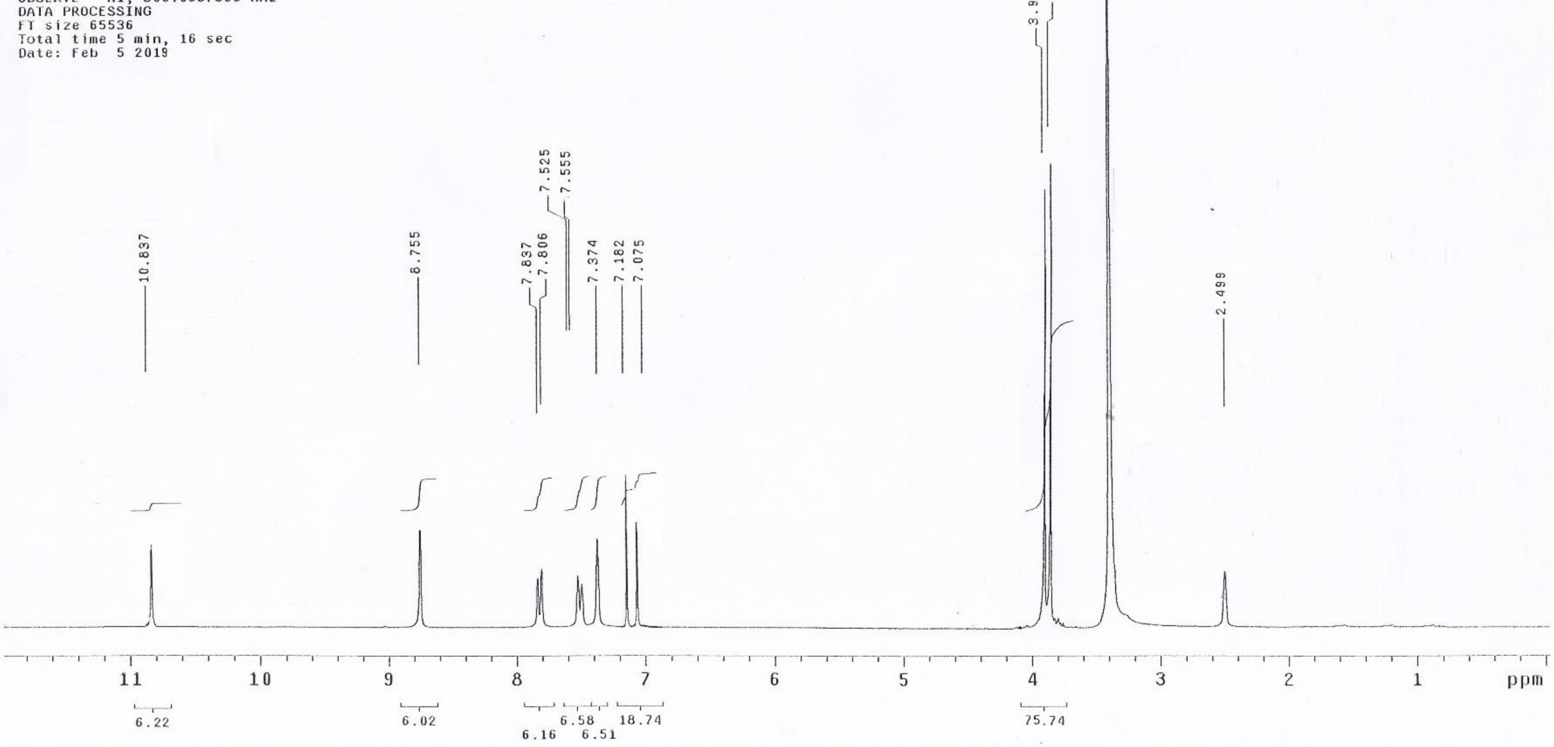

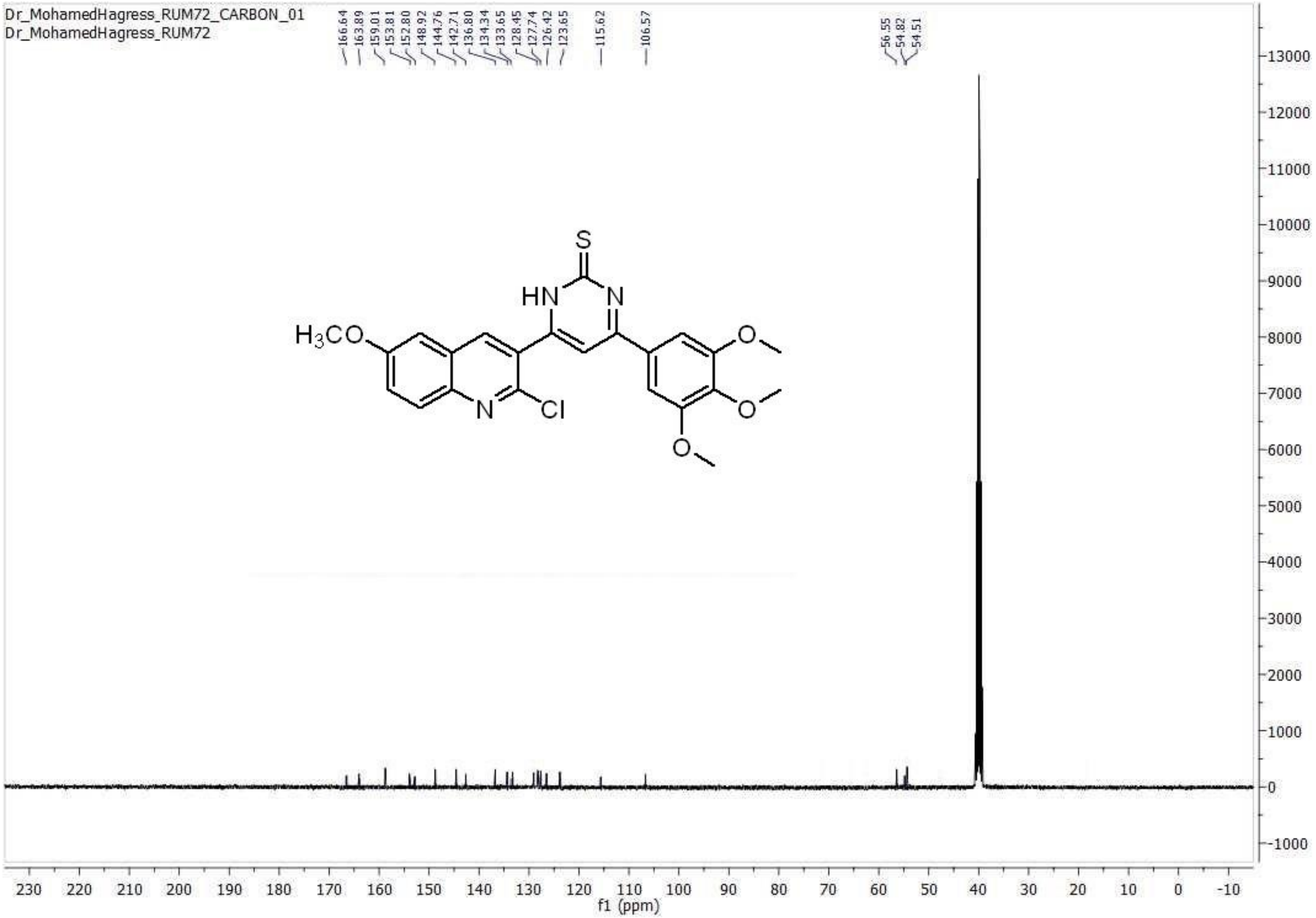

Dr_MohamedHagress_RUM72_CARBON_01
Dr_MohamedHagress_RUM72
166.64
163.89
159.01
153.81
152.80
148.92
144.76
142.71
136.80
134.34
133.65
128.45
127.74
126.42
123.65
115.62
106.57
56.55
54.82
54.51
S
HN
N
H3CO
N
Cl
O
O
O
f1 (ppm)

Dr_EmanYahia-ME3

Sample Name **Dr_EmanYahia-ME3**	Pulse sequence **PROTON**	Temperature **25**	Laboratory **MODCL**
Date collected **2018-07-18**	Solvent **DMSO**	Spectrometer **nmr400-mercury400**	NMR User **sameeh_Albadawy**

Dr_EmanYahia-ME3

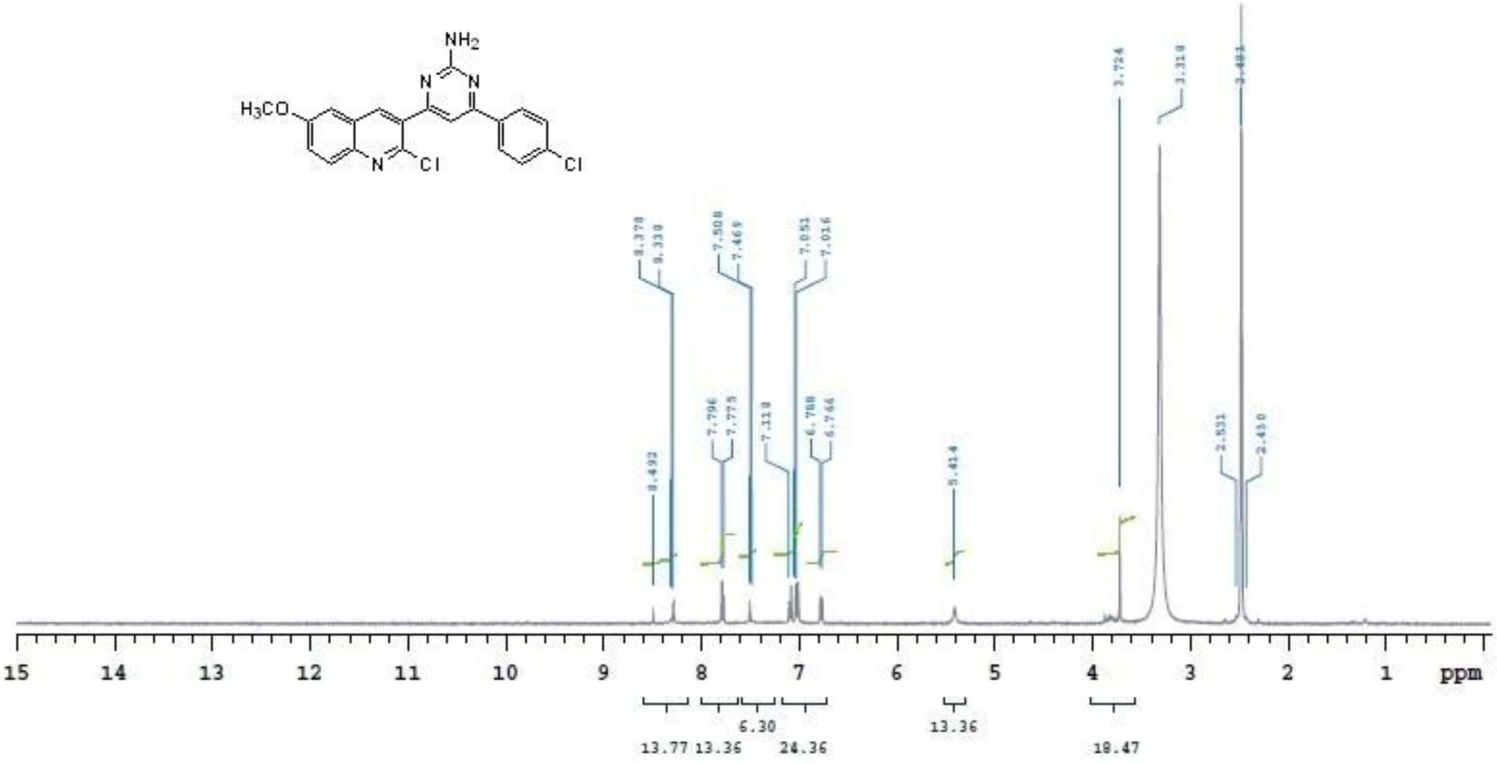

Plotname: Dr_EmanYahia-ME3_PROTON_01_plot04

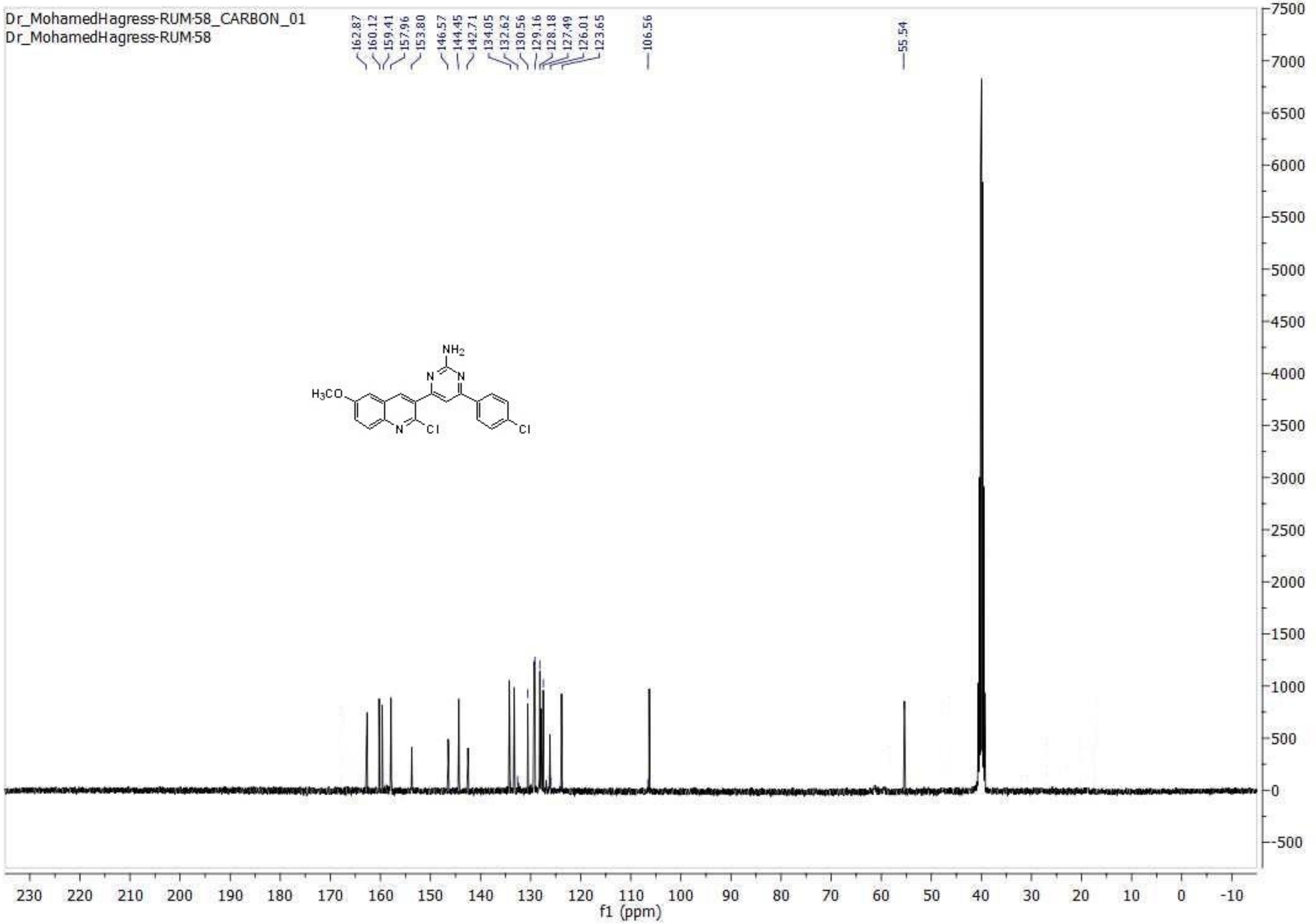
Dr_MohamedHagress-RUM-58_CARBON_01
Dr_MohamedHagress-RUM-58
162.87
160.12
159.41
157.96
153.80
146.57
144.45
142.71
134.05
132.62
130.56
129.16
128.18
127.49
126.01
123.65
106.56
55.54
NH2
N
N
H3CO
N
Cl
Cl
f1 (ppm)

Dr_EmanYahia-ME5

Sample Name Dr_EmanYahia-ME5	Pulse sequence PROTON	Temperature 25	Laboratory MODCL
Date collected 2018-07-18	Solvent DMSO	Spectrometer nmr400-mercury400	NMR User sameeh_Albadawy

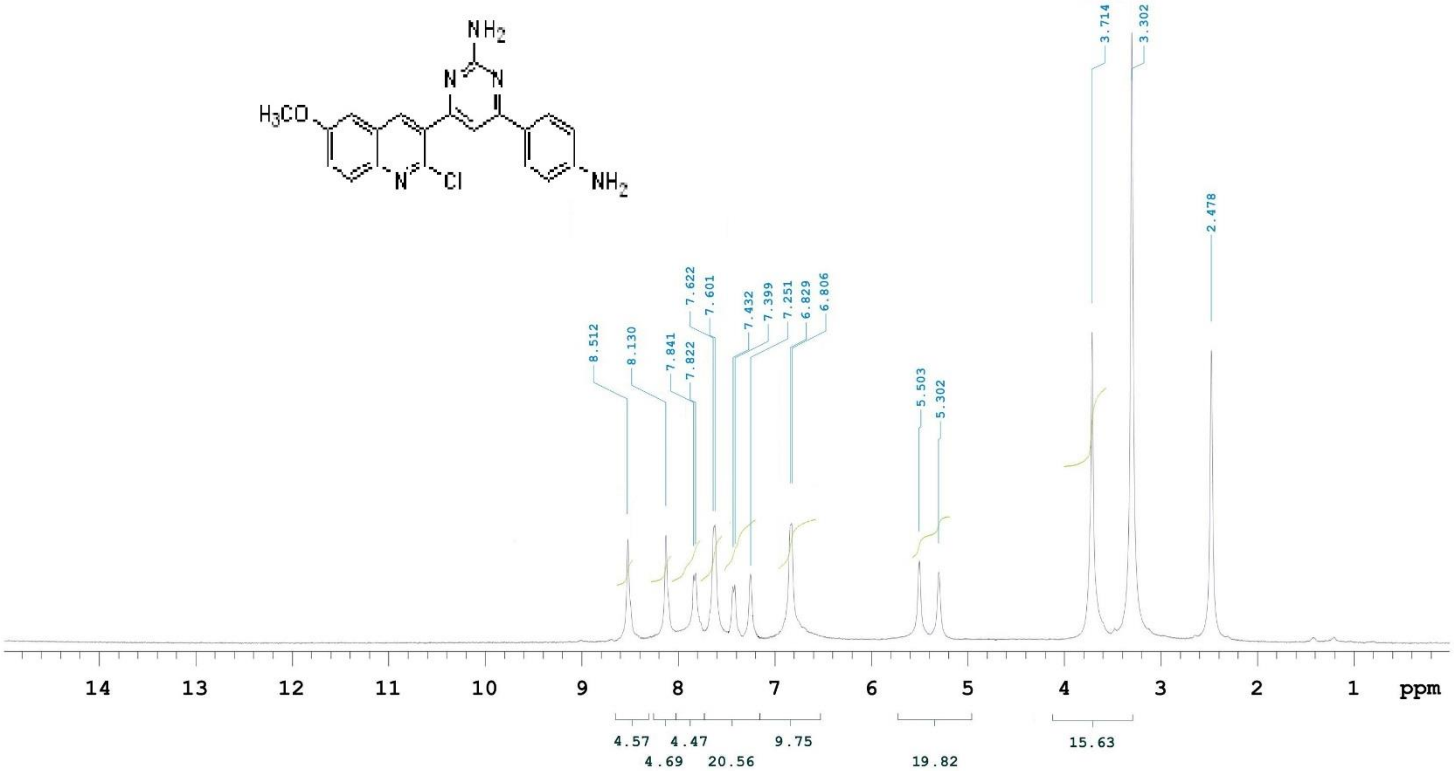

Plotname: Dr_EmanYahia-ME5_PROTON_01_plot02

Data file /home/data/NMRlab2018/Jul/Dr_EmanYahia-ME5_20180718_01/Dr_EmanYahia-ME5_PROTON_01

Plot date 2018-07-18

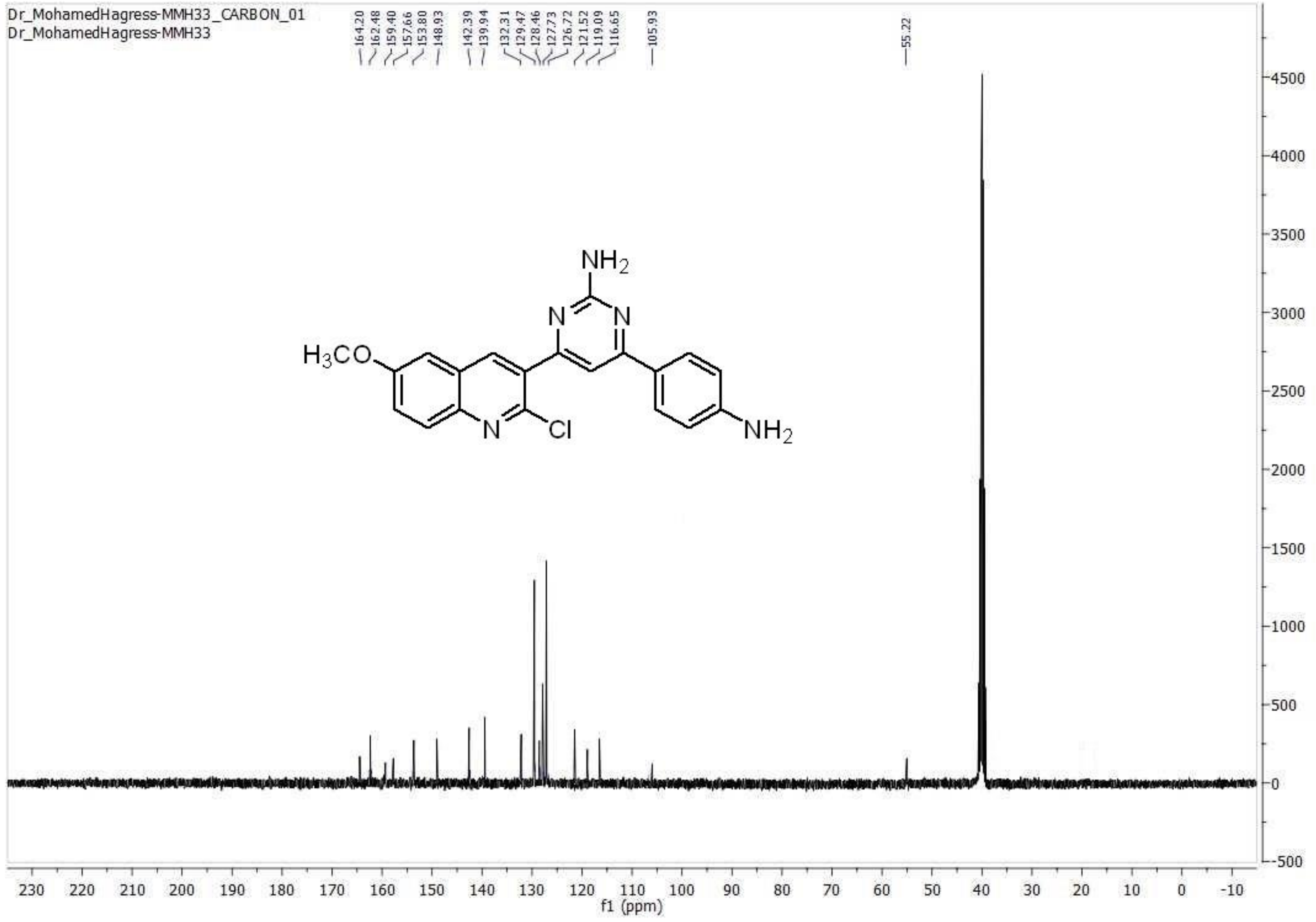

Dr_MohamedHagress-MMH33_CARBON_01
Dr_MohamedHagress-MMH33
164.20
162.48
159.40
157.66
153.80
148.93
142.39
139.94
132.31
129.47
128.46
127.73
126.72
121.52
119.09
116.65
105.93
55.22
NH2
N
N
H3CO
N
Cl
NH2
4500
4000
3500
3000
2500
2000
1500
1000
500
0
-500
230 220 210 200 190 180 170 160 150 140 130 120 110 100 90 80 70 60 50 40 30 20 10 0 -10
f1 (ppm)

Mohamed Samy-CA4-DMSO-H1

Archive directory: /export/home/vnmr1/vnmrsys/data
Sample directory: DD5mm_test_12Mar2019-21:34:40
File: PROTON

Pulse Sequence: s2pul

Solvent: DMSO
Temp. 30.0 C / 303.1 K
Mercury-300BB "NMR300"

Relax. delay 1.000 sec
Pulse 45.0 degrees
Acq. time 4.853 sec
Width 6600.7 Hz
12 repetitions
OBSERVE H1, 300.0687865
DATA PROCESSING
FT size 65536
Total time 5 min, 16 sec
Date: Feb 5 2019

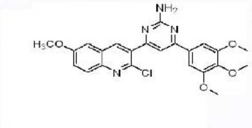

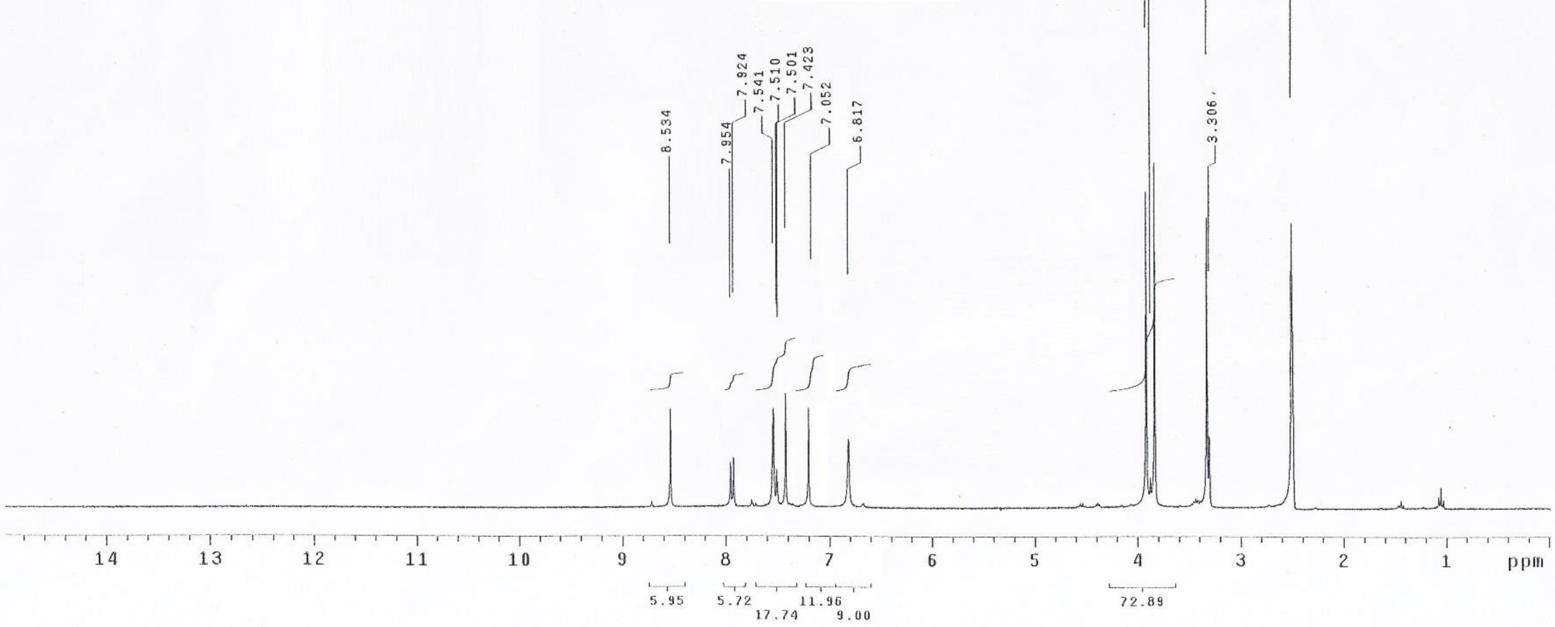

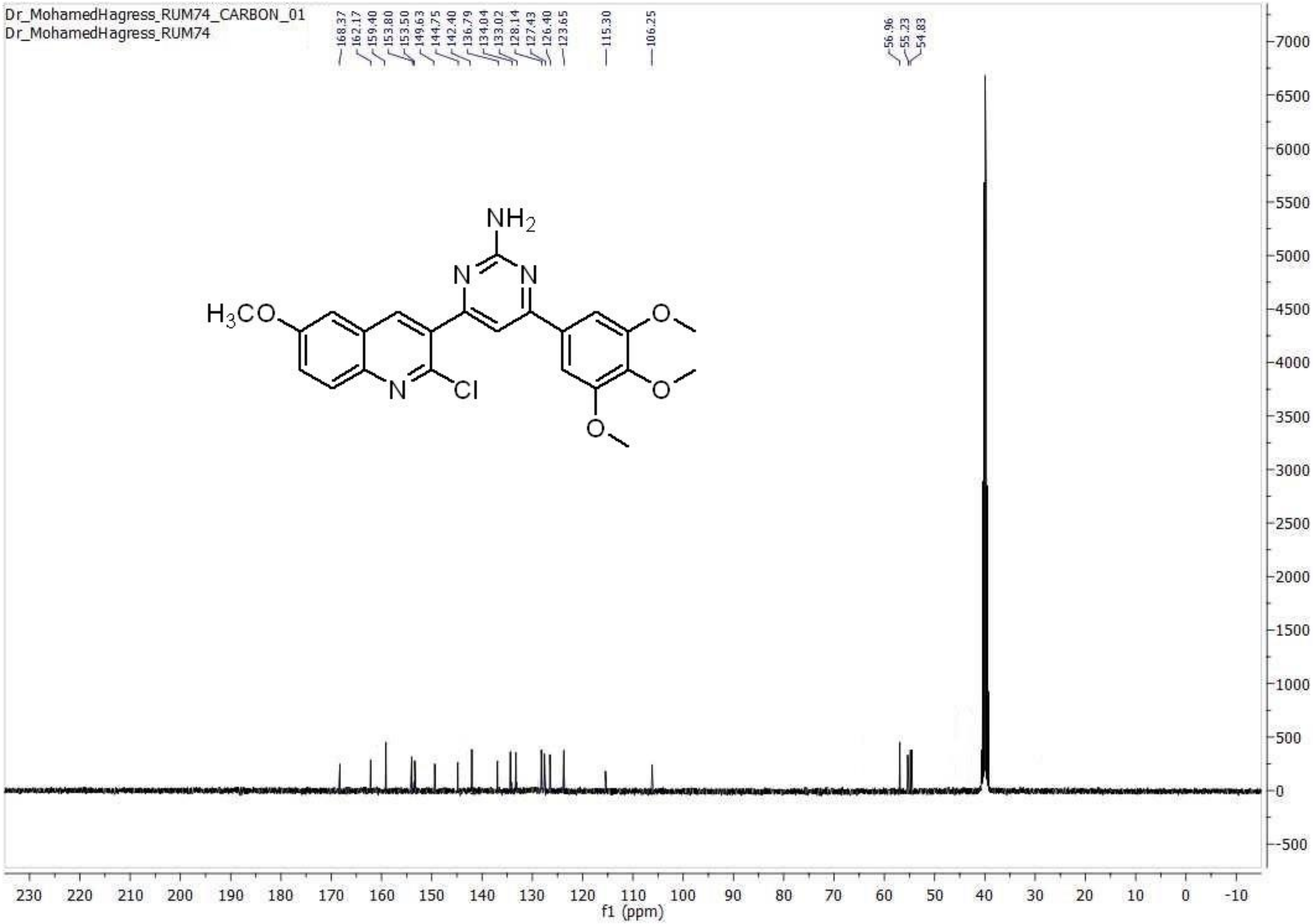
Dr_MohamedHagress_RUM74_CARBON_01
Dr_MohamedHagress_RUM74
168.37
162.17
159.40
153.80
153.50
149.63
144.75
142.40
136.79
134.04
133.02
128.14
127.43
126.40
123.65
115.30
106.25
56.96
55.23
54.83
NH2
N
N
H3CO
N
Cl
O
O
O
f1 (ppm)

Dr_EmanYahia-ME4

Sample Name **Dr_EmanYahia-ME4**	Pulse sequence **PROTON**	Temperature **26**	Laboratory **MODCL**
Date collected **2018-07-18**	Solvent **DMSO**	Spectrometer **nmr400-mercury400**	NMR User **sameeh_Albadawy**

Dr_EmanYahia-ME4

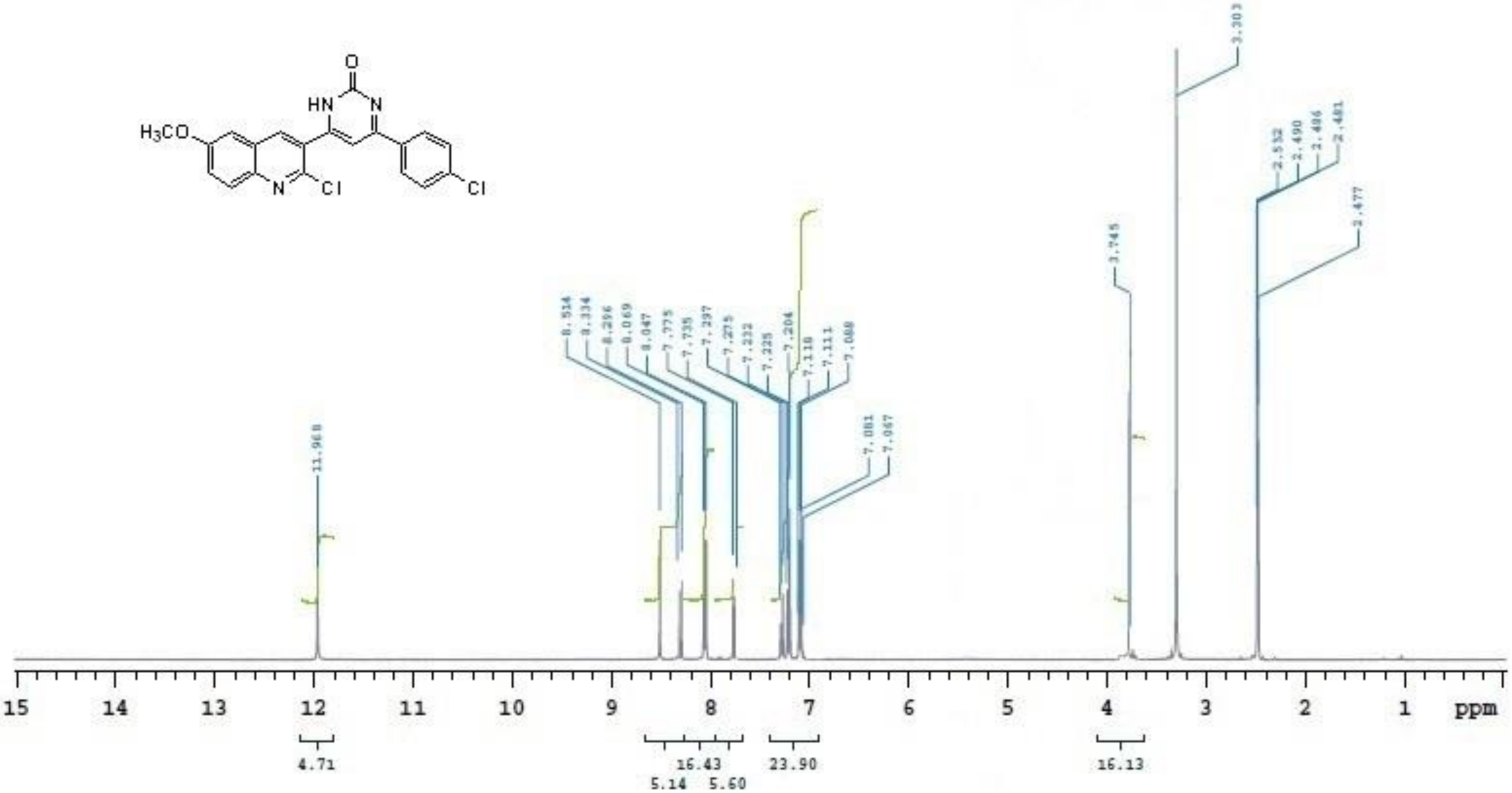

Plotname: Dr_EmanYahia-ME4_PROTON_01_plot02

Data file /home/data/NMRlab2018/Jul/Dr_EmanYahia-ME4_20180718_01/Dr_EmanYahia-ME4_PROTON_01

Plot date 2018-07-18

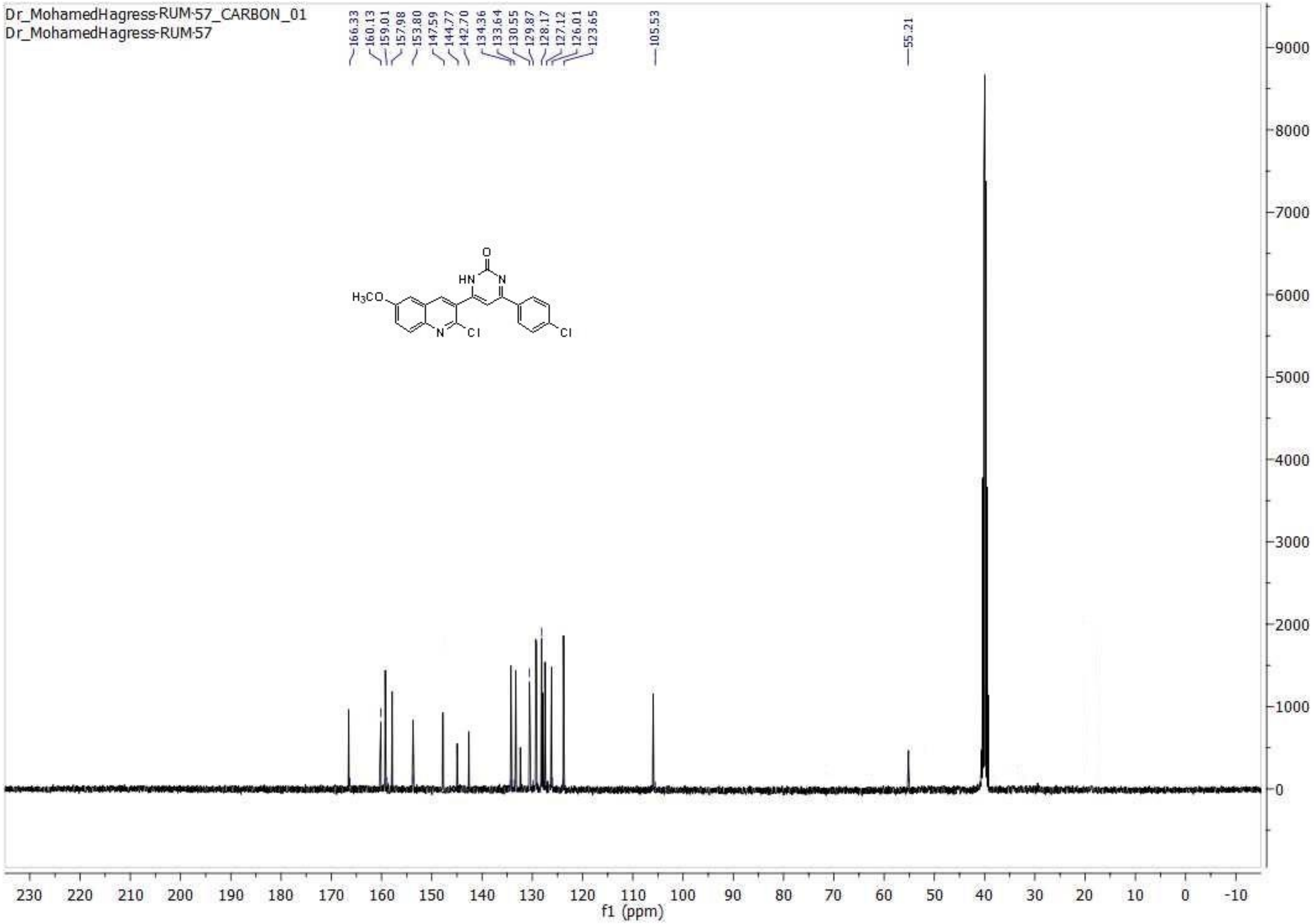

Dr_MohamedHagress-RUM-57_CARBON_01
Dr_MohamedHagress-RUM-57
166.33
160.13
159.01
157.98
153.80
147.59
144.77
142.70
134.36
133.64
130.55
129.87
128.17
127.12
126.01
123.65
105.53
55.21
H_3CO
HN
N
O
N
Cl
Cl
f1 (ppm)

Dr_EmanYahia-ME-1e

Dr_EmanYahia-ME-1e

Sample Name	Dr_EmanYahia-ME-1e	Pulse sequence	PROTON	Temperature	25	Laboratory	MODCL
Date collected	2018-03-19	Solvent	DMSO	Spectrometer	nmr400-mercury400	NMR User	sameeh_Albadawy

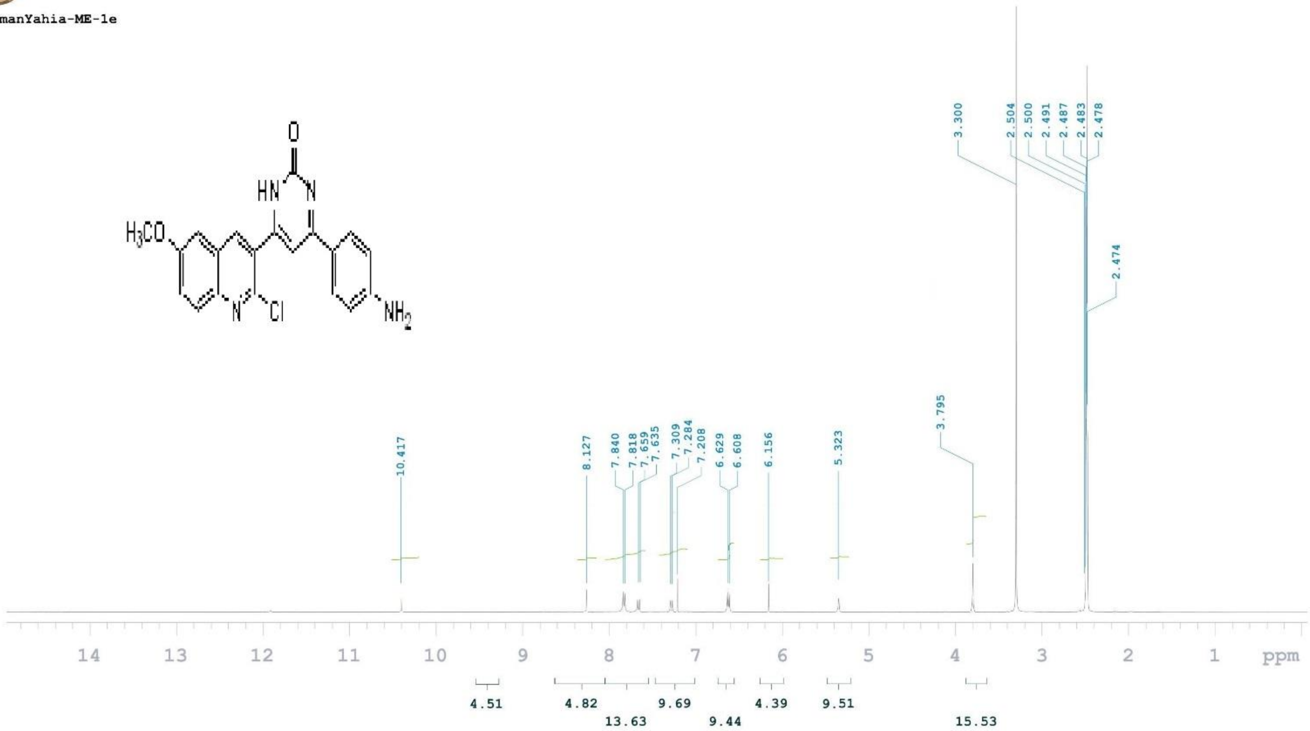

Plotname: Dr_EmanYahia-ME-1e_PROTON_01_plot02

Data file /home/data/NMRlab2018/Mar/Dr_EmanYahia-ME-1e_20180319_01/Dr_EmanYahia-ME-1e_PROTON_01

Plot date 2018-03-19

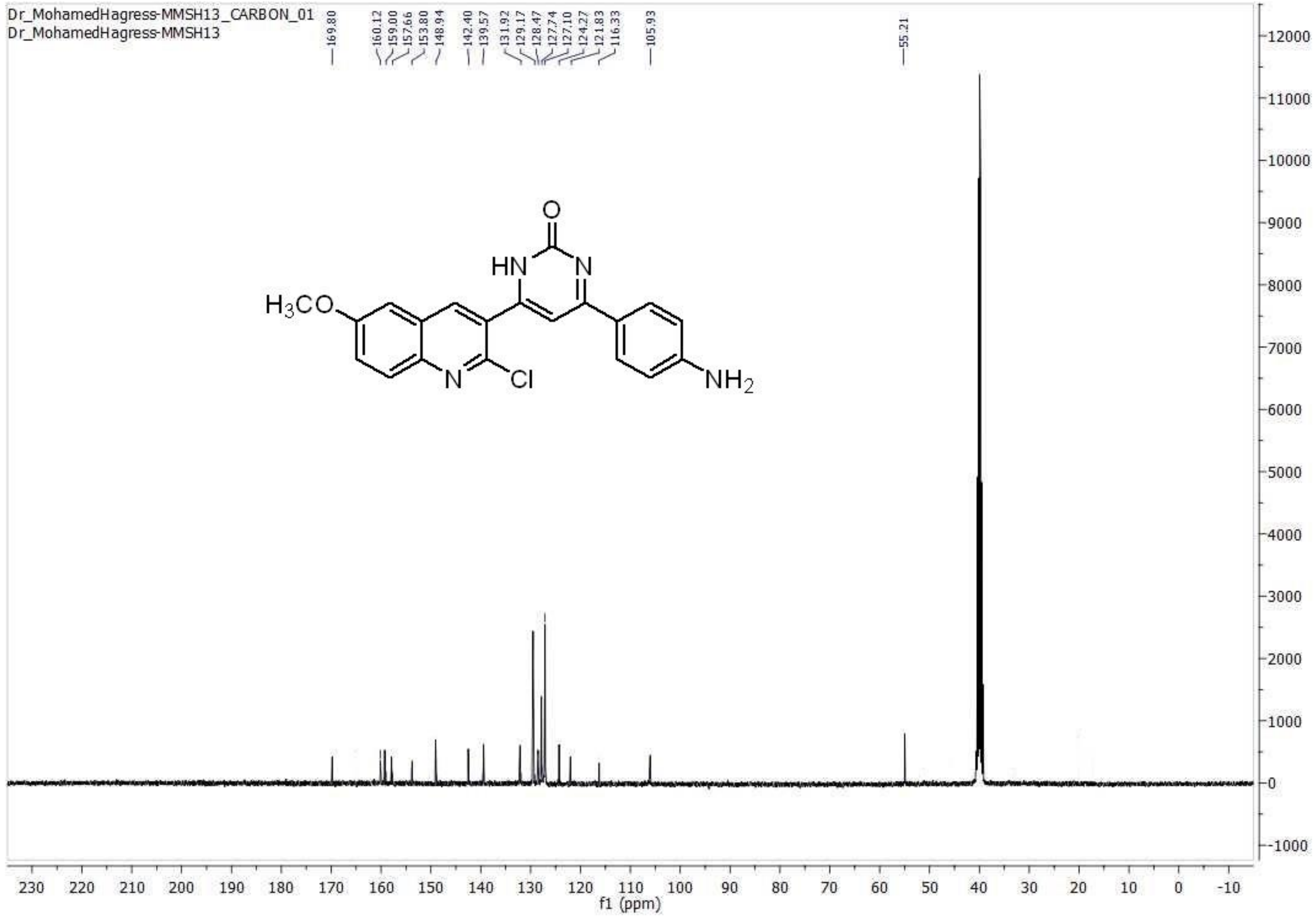
Dr_MohamedHagress-MMSH13_CARBON_01
Dr_MohamedHagress-MMSH13
169.80
160.12
159.00
157.66
153.80
148.94
142.40
139.57
131.92
129.17
128.47
127.74
127.10
124.27
121.83
116.33
105.93
55.21
H3CO
HN
N
O
N
Cl
NH2
f1 (ppm)

Mohamed Samy-CA5-DMSO-H

Archive directory: /export/home/vnmr1/vnmrsys/data
Sample directory: DD5mm_test_12Mar2019-21:34:40
File: PROTON

Pulse Sequence: s2pul

Solvent: DMSO
Temp. 30.0 C / 303.1 K
Mercury-300BB "NMR300"

Relax. delay 1.000 sec
Pulse 45.0 degrees
Acq. time 4.853 sec
Width 6600.7 Hz
12 repetitions
OBSERVE H1, 300.0687865 MHz
DATA PROCESSING
FT size 65536
Total time 5 min, 16 sec
Date: Jan 20 2019

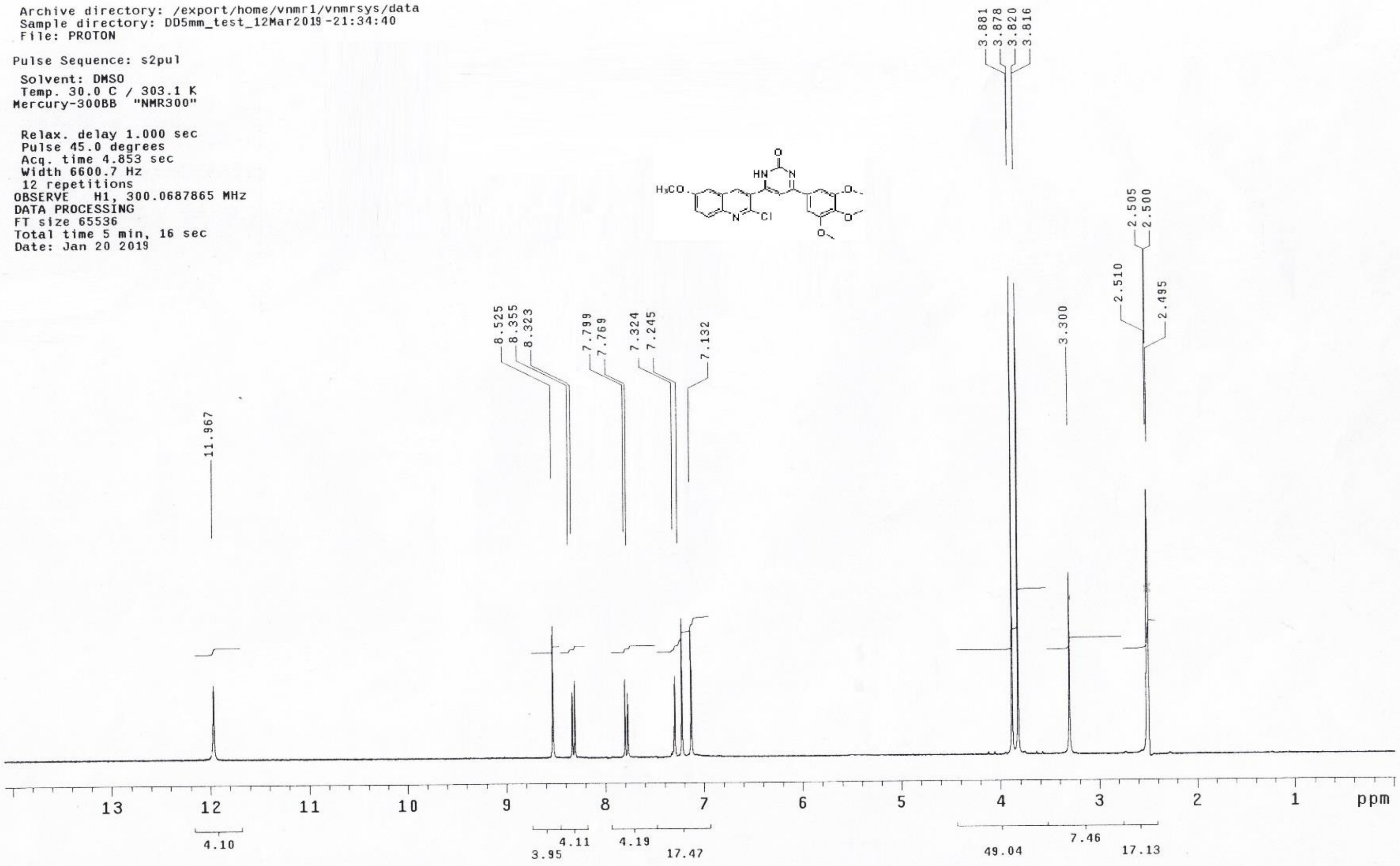

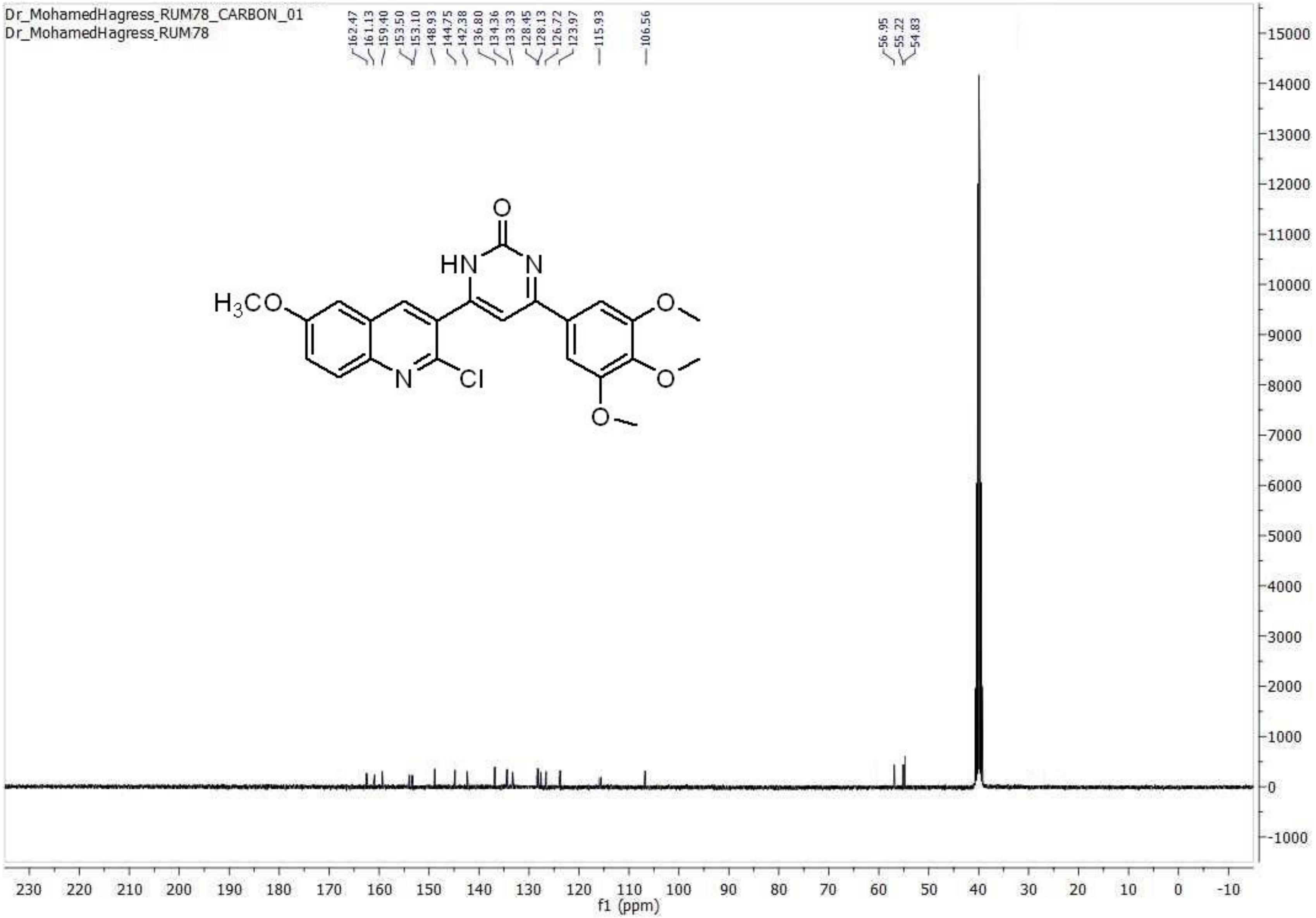
Dr_MohamedHagress_RUM78_CARBON_01
Dr_MohamedHagress_RUM78
162.47
161.13
159.40
153.50
153.10
148.93
144.75
142.38
136.80
134.36
133.33
128.45
128.13
126.72
123.97
115.93
106.56
56.95
55.22
54.83
HN
N
O
H_3CO
N
Cl
O
O
O
f1 (ppm)
